# Design, synthesis, and biological evaluation of piperazine derivatives involved in the 5-HT_1A_R/BDNF/PKA pathway

**DOI:** 10.1080/14756366.2023.2286183

**Published:** 2023-12-11

**Authors:** Hao Zhou, Mengjiao Li, Hui Liu, Zheng Liu, Xuekun Wang, Shiben Wang

**Affiliations:** a School of Pharmaceutical Sciences, Liaocheng University, Liaocheng, Shandong, China; b College of Pharmacy, Yanbian University, Yanji, Jilin, China; c School of Medicine, Foshan University, Foshan, Guangdong, China

**Keywords:** Piperazine, antidepressant, western blot, 5-HT_1A_R, molecular docking studies

## Abstract

In this study, four series of piperazine derivatives were designed, synthesised and subjected to biological test, and compound **6a** with potential antidepressant activity was obtained. An affinity assay of compound **6a** with 5-hydroxytryptamine (serotonin, 5-HT)_1A_ receptor (5-HT_1A_R) was undertaken, and the effects on the 5-HT level in the brains of mice were also tested. The results showed that compound **6a** had the best affinity with 5-HT_1A_R (*K*_i_ = 1.28 nM) and significantly increased the 5-HT level. The expression levels of 5-HT_1A_R, BDNF, and PKA in the hippocampus were analysed by western blot and immunohistochemistry analyses. The results showed that the expression of 5-HT_1A_R, BDNF, and PKA in the model group was reduced compared to that of the control group, and compound **6a** could reverse this phenomenon. Molecular docking was performed to investigate the interactions of the studied compound **6a** with 5-HT_1A_R on the molecular level.

## Graphical Abstract

**Figure:**
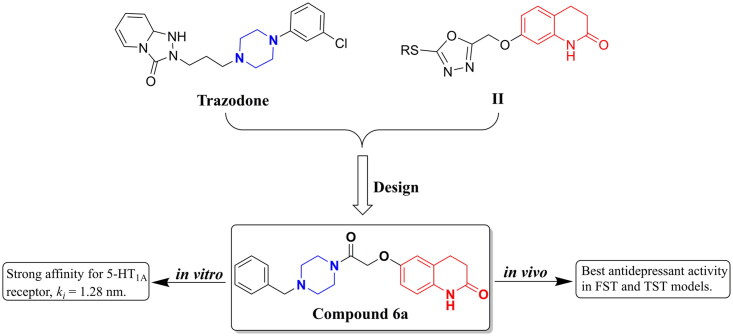

## Introduction

Depression is a common mental illness, a major contributor to disability and death by suicide, and can cause a significant socio-economic burden^1^. The aetiology of depression is unclear, and it is generally accepted that depression is a multifactorial disorder caused by the interaction of social, psychological, and biological aspects. The World Health Organisation (WHO) predicts that depression as a disorder will be ranked number one worldwide by 2030^2^. Currently, antidepressants are a major treatment for depression, and although many advances have been made in the field of drug development, there are limited antidepressants available for the clinical treatment of depression and most cause a variety of adverse effects^3^. Therefore, the development of additional potential antidepressant molecules has become a major research hotspot in the field of pharmaceutical research.

**Figure 1.**
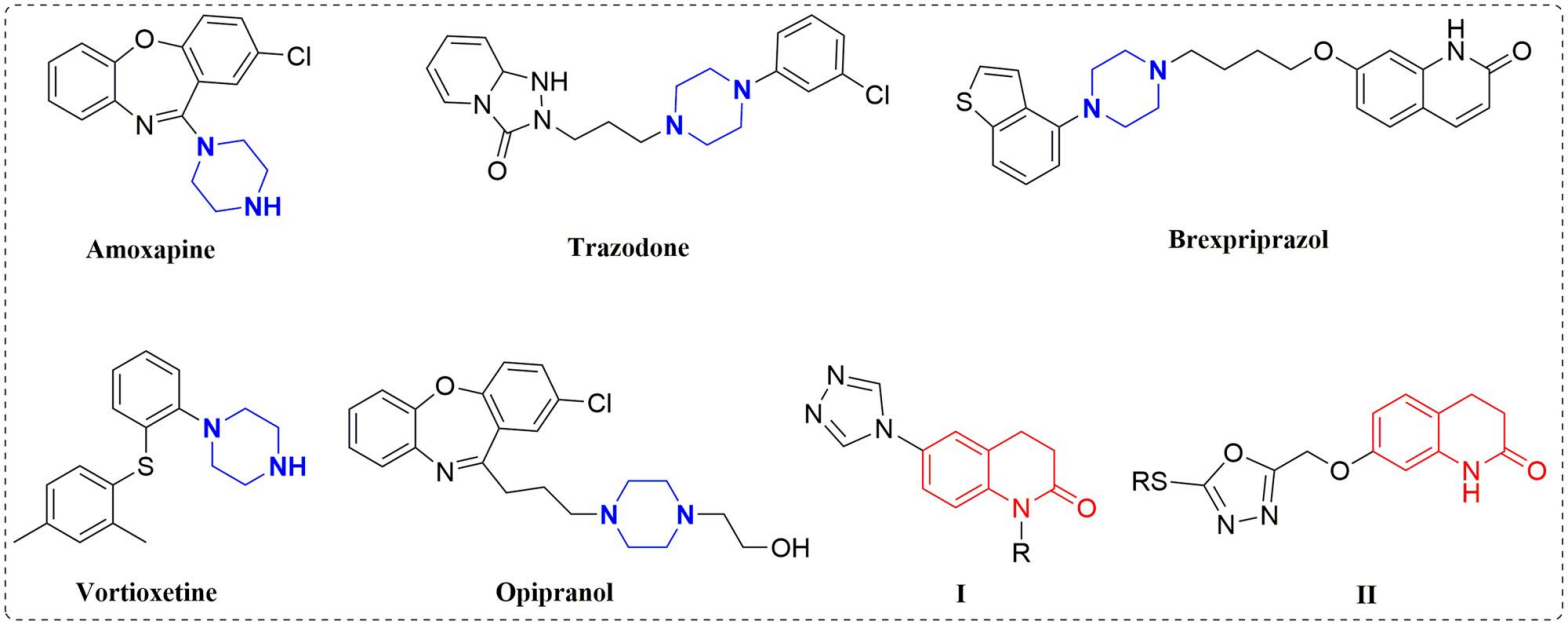
The chemical structures of piperazine or 3,4-dihydroquinolin-2(1*H*)-one derivatives used for antidepressants.

Piperazine scaffolds are important moieties to consider in drug design and are widely used in drug discovery. They link to other structural fragments to obtain new molecules with a wide range of biological activities, such as antimicrobial^4^, antitubercular^5^, antidepressant^6^, anticancer^7^, antileishmanial^8^, and antihela^9^ activities. Many clinical antidepressants or antidepressant molecules have piperazine structural fragments, such as amoxapine, trazodone, opipramol, vortioxetine, and brexpriprazole (Figure 1). 3,4-Dihydroquinolin-2(1*H*)-one is also an important structural fragment that has been widely used in drug design and has shown various biological activities^10–12^. In the field of antidepressant drug development, there have been relatively few reports of antidepressant molecules designed with 3,4-dihydroquinolin-2(1*H*)-one structures. A series of 3,4-dihydroquinolin-2(1*H*)-one derivatives (**I**) was designed and synthesised by Deng et al., and some derivatives showed antidepressant activity^13^. In addition, a series of 3,4-dihydroquinolin-2(1*H*)-one derivatives (**II**) was designed and synthesised by our group, and most of the compounds showed good antidepressant activity. This series of compounds was found to have a good affinity for 5-hydroxytryptamine (5-HT)_1A_ receptor (5-HT_1A_R)^14^ (Figure 1).

**Figure 2.**
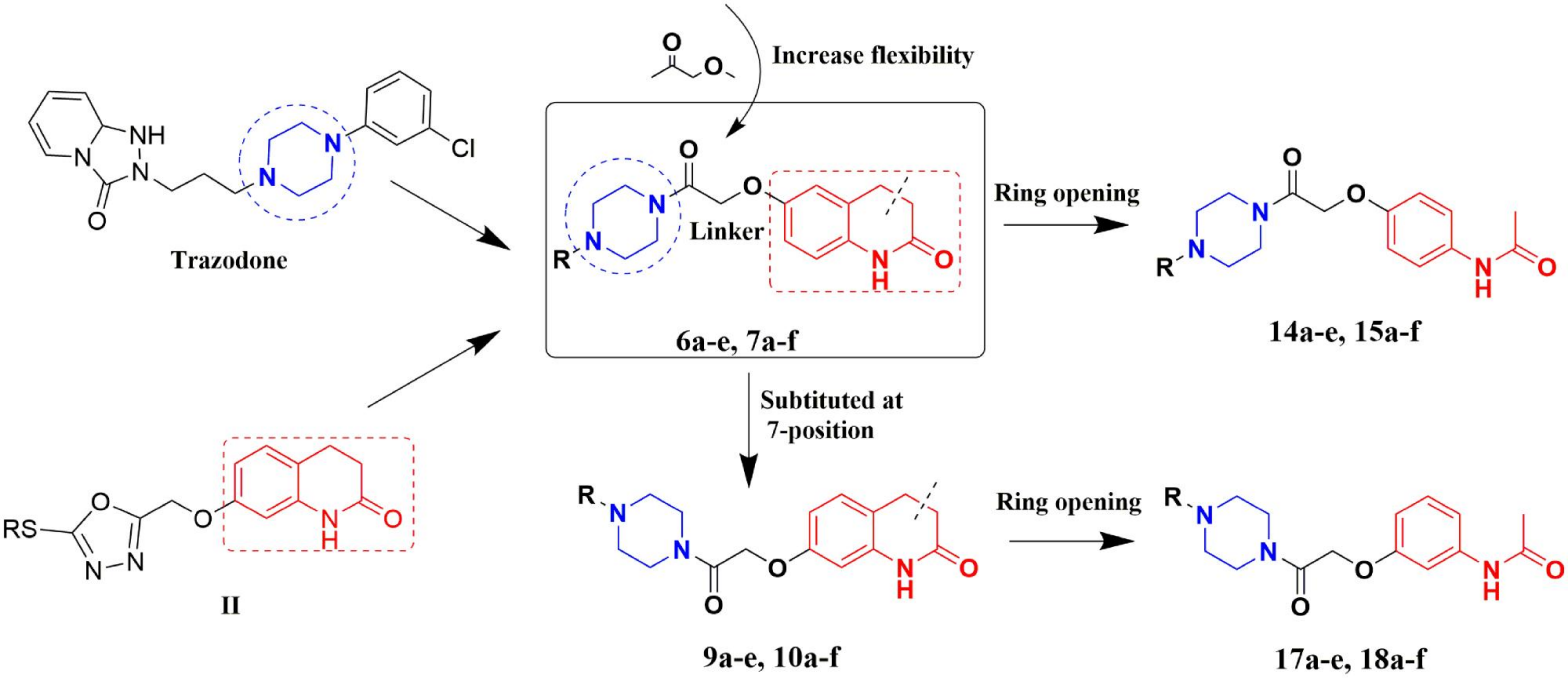
Rationale design of piperazine and 3,4-dihydroquinolin-2(1*H*)-one derivatives.

Here, a series of target compounds, **6a**–**e**, **7a**–**f**, **9a**–**e**, and **10a**–**f**, was synthesised by combining 3,4-dihydroquinolin-2(1*H*)-one and piperazine structural fragments, and the effect of their structures on antidepressant activity was investigated. To verify the effect of the 3,4-dihydroquinolin-2(1*H*)-one structural fragment on antidepressant activity, the C-C double bonds at the three and four positions were cut. A series of target compounds, **14a**–**e**, **15a**–**f**, **17a**–**e**, and **18a**–**f**, was designed and synthesised by increasing the flexibility of the molecules, and the effects of the ring openings on the antidepressant activity were explored (Figure 2).

In pharmacological studies, the forced swimming test (FST) and tail suspension test (TST) methods were used to test the *in vivo* antidepressant activity of the target compound. The open field test (OFT) method was used to investigate whether the target compound affected spontaneous behaviour in mice. A 5-HT_1A_R binding assay was performed on the potential compounds obtained by FST screening to investigate their possible mechanisms of action. The 5-HT concentration in the brains of mice was tested using enzyme-linked immunosorbent assay (ELISA) kits to verify the effects of the potential compounds on the 5-HT level in the brains of mice. A chronic unpredictable mild stress (CUMS) model was established and the effects of the potential compounds on the 5-HT_1A_R/BDNF/PKA signalling pathway were analysed by western blot and immunohistochemical analyses. The molecular formulae of all of the target compounds were constructed using Sketch Molecules in the Small Molecules module of the Discovery Studio (DS) 2021 software. The physicochemical and pharmacokinetic parameters were predicted for all of the target compounds (Table 2 and Figure 14, in Supplementary File), and their structures were confirmed by ^1^H-NMR,^13^C-NMR, and high-resolution mass spectra (HRMS) techniques.

## Results and discussion

### Chemistry

**Scheme 1.**
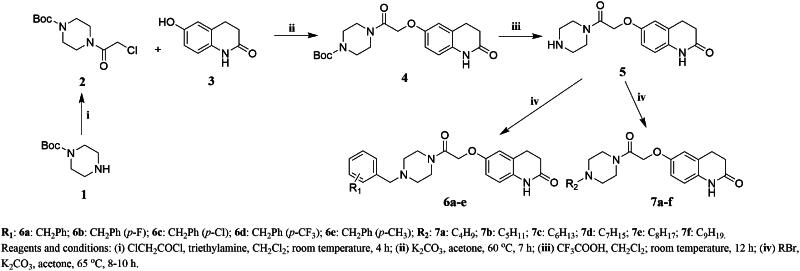
Synthesis of the target compounds **6a-e** and **7a-f**.

**Scheme 2.**
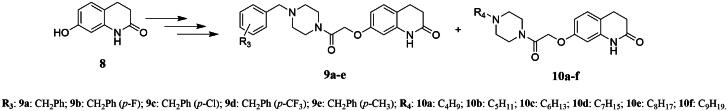
Synthesis of the target compounds **9a-e** and **10a-f**.

**Scheme 3.**
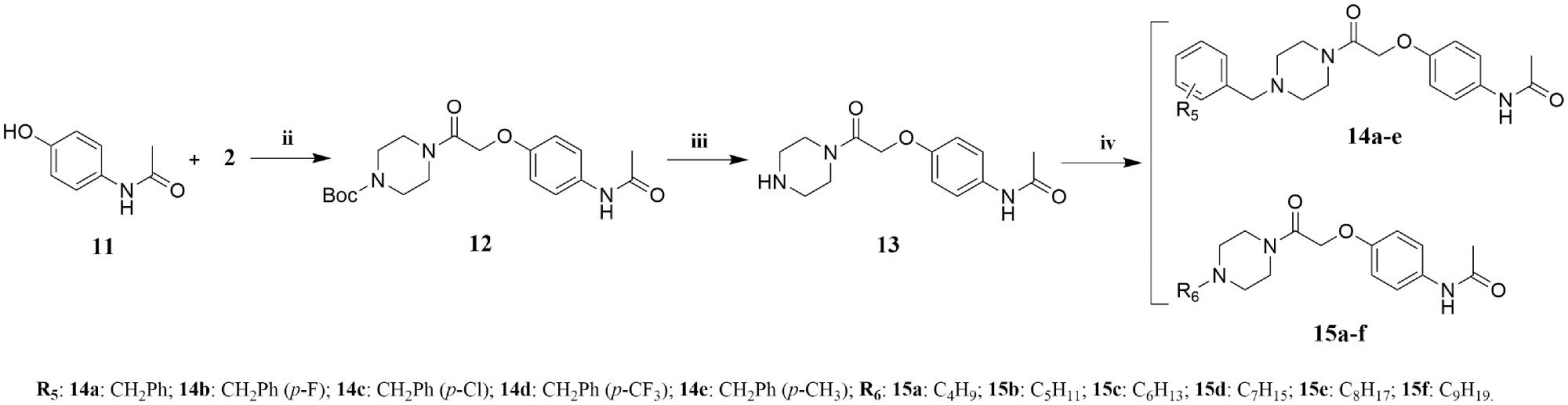
Synthesis of the target compounds **14a-e** and **15a-f**.

**Scheme 4.**
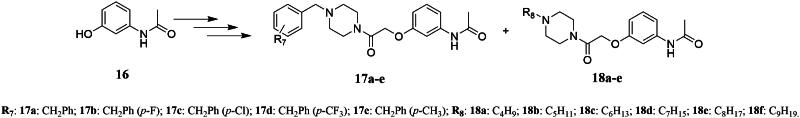
Synthesis of the target compounds **17a-e** and **18a-f**.

The synthetic routes for the target compounds **6a–e**, **7a–f**, **9a–e**, **10a–f**, **14a–e**, **15a–f**, **17a–e**, and **18a–f** are shown in Schemes 1–4. 1-Boc-piperazine was reacted with chloroacetyl chloride in an ice bath to obtain Intermediate **2**. The reaction temperature in this step was controlled to prevent an increase in by-product formation. Intermediate **2** was reacted with 3,4-dihydro-6-hydroxy-2(1*H*)-quinolinone (**3**) under alkaline conditions to give Intermediate **4**, which was obtained with a high yield. Intermediate **5** was synthesised by the removal of the Boc group using trifluoroacetic acid, which is a mild reaction with a high yield, to give an oily liquid after treatment. The reaction of Intermediate **5** with various substituted benzyl bromides and brominated alkanes under alkaline conditions resulted in the target compounds **6a–e** and **7a–f**. The target compounds **9a–e**, **10a–f**, **14a–e**, **15a–f**, **17a–e**, and **18a–f** and their intermediates were synthesised in a similar way to those of Intermediate **4** and **5** and the target compounds **6a–e** and **7a–f**. The structures of all of the target compounds were confirmed by ^1^H-NMR,^13^C-NMR, and HRMS techniques.

### Biological evaluations

#### FST

**Figure 3.**
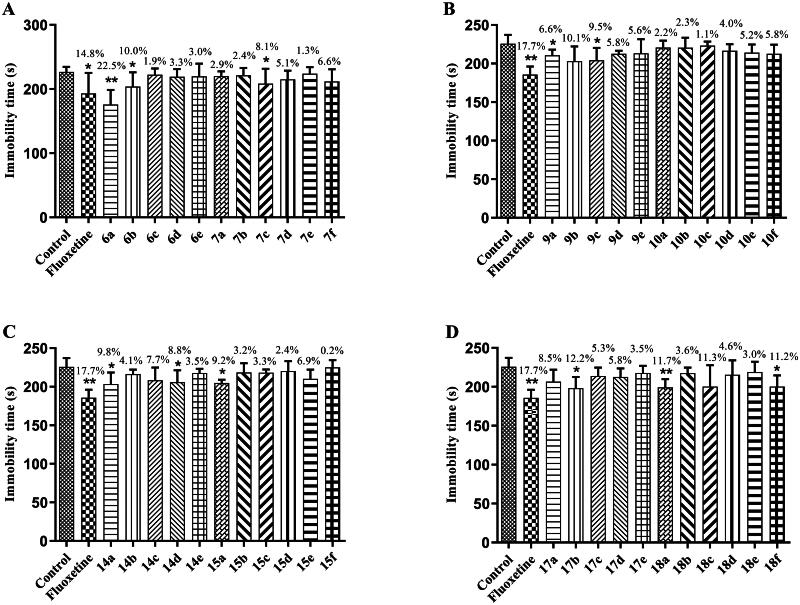
Antidepressant activities of target compounds and fluoxetine in FST at 40 mg/kg. Values represent the mean ± SEM (*n* = 8). *p < 0.05, **p < 0.01 *vs* the control group.

The antidepressant activity of the target compounds was screened by the FST model. Three mouse groups were established: control, positive control (fluoxetine), and compound groups. The positive control and compound groups were administered 40 mg/kg of the relevant substance, and the control group was administered an equal volume of solvent. The antidepressant activity of all groups was measured after 0.5 h of intraperitoneal administration. The experimental results are displayed in Figure 3, which shows that some of the target compounds had antidepressant activity, with compounds **6b**, **7c**, **9a**, **9c**, **14a**, **14d**, **15a**, **17b**, and **18f** showing antidepressant activity *vs* the control group (*p < 0.05), shortening the immobility time in the mice. Compound **6a** (**p < 0.01) and **18a** (**p < 0.01) groups *vs* the control group showed significantly reduced immobility time in mice, similar to fluoxetine. Therefore, among all of the target compounds, **6a** and **18a** were considered to show potential as antidepressants.

**Figure 4.**
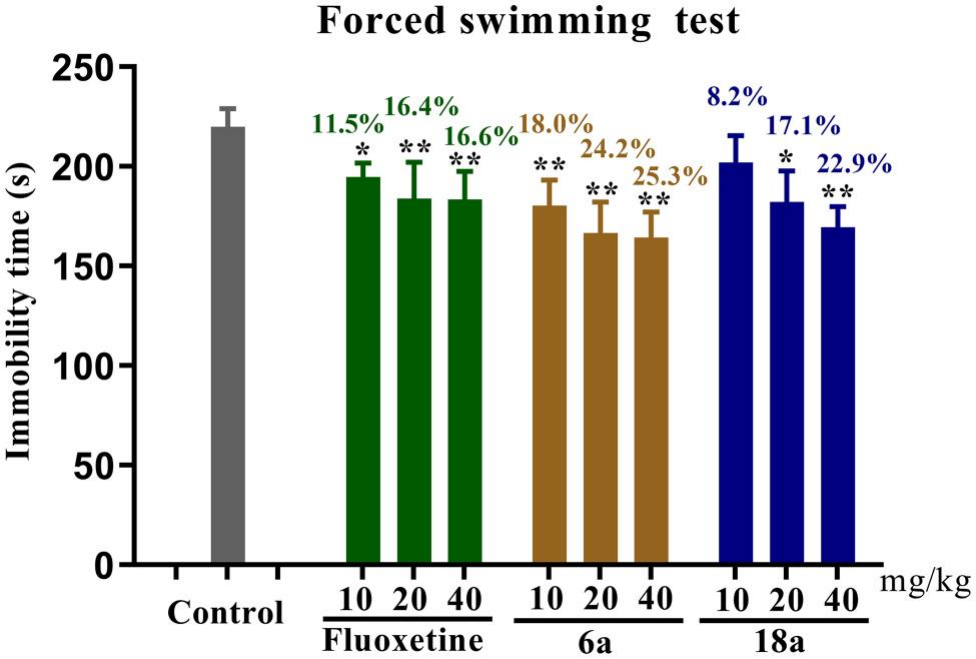
Antidepressant activities of compounds **6a** and **18a** as well as those of fluoxetine in FST at different doses (40, 20, and 10 mg/kg). Values represent the mean ± SEM (*n* = 8). *p < 0.05 and **p < 0.01 *vs* the control group.

In order to verify the dose-dependence of the antidepressant activity of the compounds, the potential compounds **6a** and **18a** were tested using the FST model for their antidepressant activity at different doses (10, 20, and 40 mg/kg). The experimental results (Figure 4) showed that as the dose increased, the immobility time of compounds **6a** and **18a** as well as that of fluoxetine significantly decreased in mice, with the best antidepressant activity at 40 mg/kg (**p < 0.01). Compounds **6a** and **18a** shortened the immobility time by 25.3% and 22.9%, respectively, while fluoxetine shortened the immobility time by 16.6%.

#### TST

**Figure 5.**
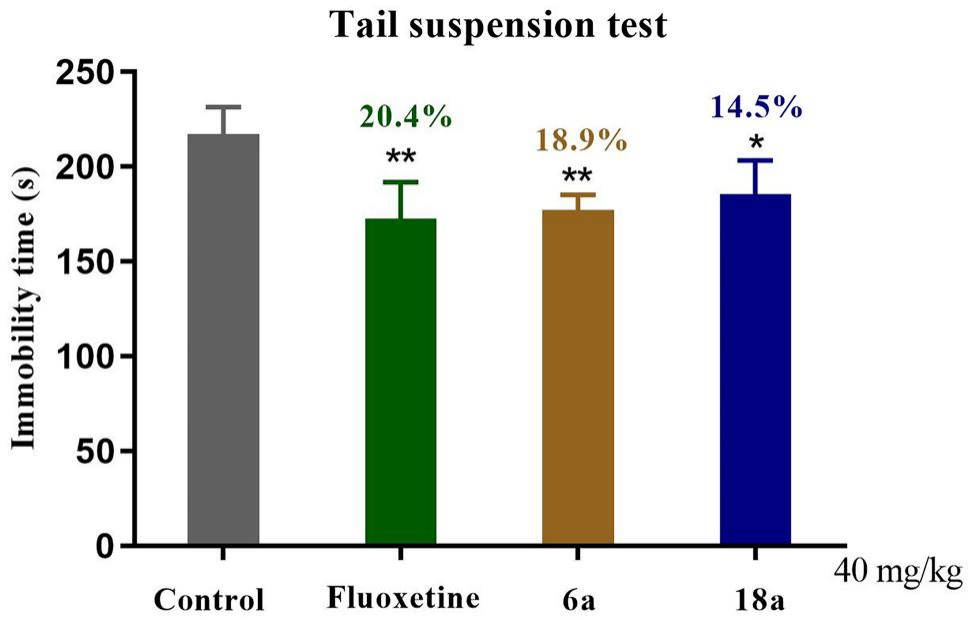
Antidepressant activity of compounds **6a** and **18a** as well as that of fluoxetine in the TST at 40 mg/kg. Values represent the mean ± SEM (*n* = 8). *p < 0.05, **p < 0.01 *vs* control group.

The TST model, like the FST model, is one of the most important models for the screening of antidepressant drugs. In order to confirm whether compounds **6a** and **18a** were potential antidepressants, the TST method was used to further validate their antidepressant activity. As shown in Figure 5, for compound **6a** versus control group (**p < 0.01), the immobility time in the mice was reduced by 18.9%, which was similar to that for the fluoxetine group. For compound **18a** versus control group (*p < 0.05), the immobility time was reduced by 14.5%, with weaker antidepressant activity than the fluoxetine group.

#### OFT

**Figure 6.**
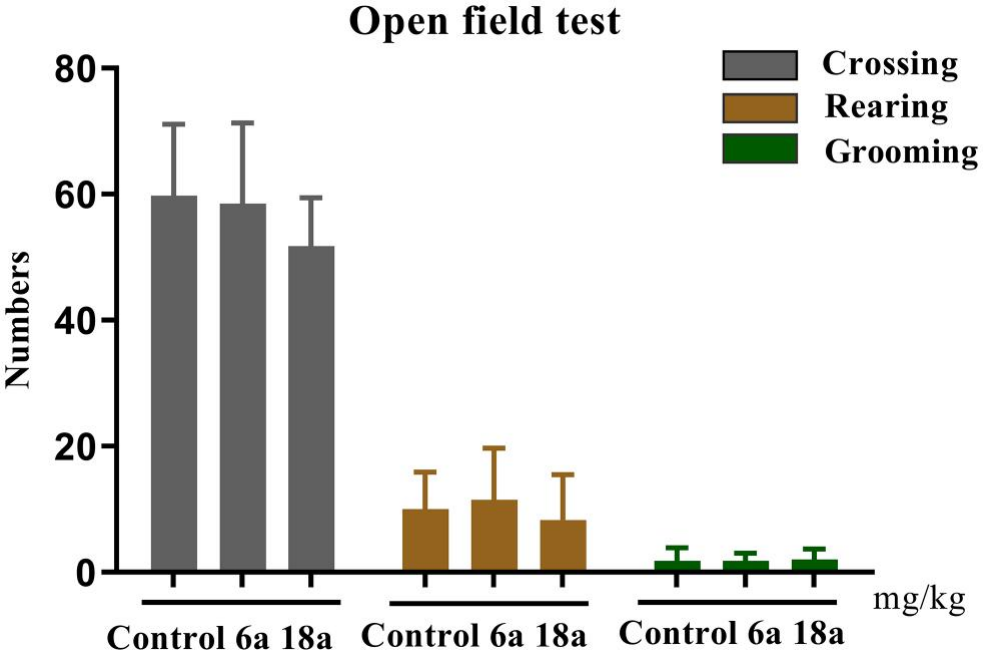
Exploratory activity (counts) in the open field test. Compounds **6a** and **18a** (40 mg/kg) were administered 1 h before the test. Locomotion: number of line crossings; Rearing: number of times seen standing on hind legs; Grooming: number of modifications. The values represent the mean ± SEM (*n* = 8).

The OFT is a popular behavioural test for the study of animal psychiatry, as it is used to observe spontaneous locomotor activity as well as for the exploration of novel environments, nervousness, mania, anxiety, and depression in experimental animals. In this study, the effects of compounds **6a** and **18a** on spontaneous activity in mice were evaluated using the crossing, rearing, and grooming indicators. The results shown in Figure 6 indicate that there was no difference between the compound **6a** and **18a** groups *vs* control group (*p* > 0.05) in the three indicators tested.

##### 5-HT level

**Figure 7.**
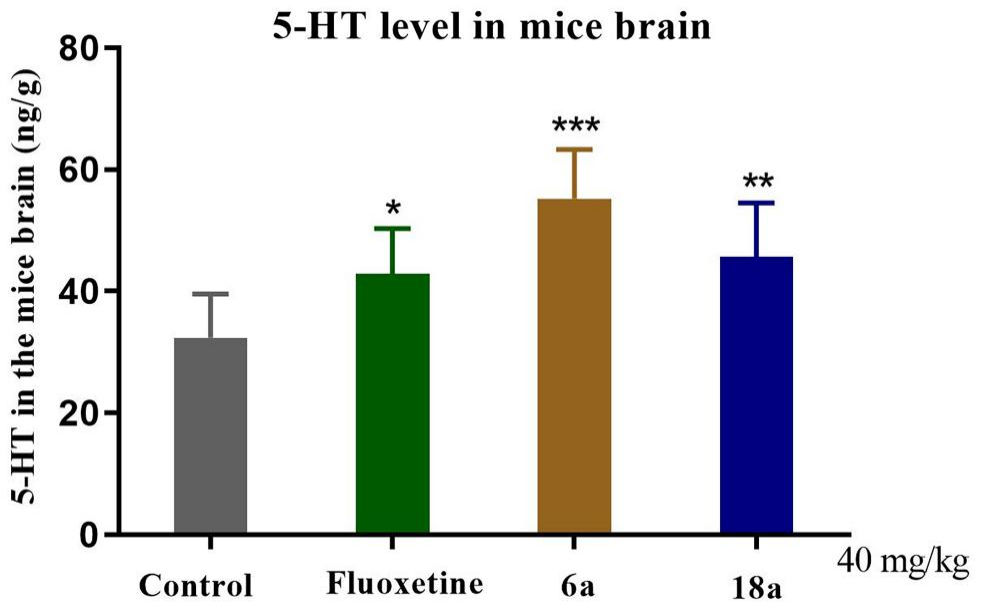
Effect of compounds **6a** and **18a** as well as that of fluoxetine on the brain 5-HT level in mice (40 mg/kg). Compounds **6a** and **18a** as well as fluoxetine were analysed 1 h after oral administration (concentration of 5-HT in the brains of mice). Values represent the mean ± SEM (*n* = 8). *p < 0.05, **p < 0.01, ***p < 0.001 *vs* control group.

5-HT is an important monoamine neurotransmitter in the CNS that can reduce the 5-HT level in the brain and is closely related to the pathogenesis of depression^15^. Most clinical antidepressants have antidepressant effects by enhancing the neurotransmission function of 5-HT and increasing the level of 5-HT in the synapses. Figure 7 shows that in the compound **6a** (***p < 0.001) and **18a** (**p < 0.01) groups versus control group, the 5-HT level in the brains of the mice was significantly increased. In the fluoxetine group *vs* control group (**p < 0.01), fluoxetine significantly increased the 5-HT level in the brains of the mice (**p < 0.01). The results of the pharmacological experiments indicated that compounds **6a** and **18a** had a modulating effect on hippocampal 5-HT levels in mice.

##### 5-HT_1A_R binding assay

5-HT_1A_R plays an important role in the pathogenesis of depression, and the drug molecules exert antidepressant activity by activating 5-HT_1A_R in the postsynaptic membrane, mediating cellular physiological effects to induce biological effects^16^. Compounds **6a** and **18a** were selected for affinity assays with 5-HT_1A_R. The *ki* values of the compounds with 5-HT_1A_R were determined and the possible mode of action of the potential compounds was inferred based on the strength of the affinity (Table 1). Compounds **6a** and **18a** showed strong affinity for 5-HT_1A_R, with *ki* values of 1.28 and 1.66 nm, respectively, which were similar to those of the neurotransmitter serotonin. Based on the results of the pharmacological experiments, it was speculated that the potential compounds may exert antidepressant effects by acting on 5-HT_1A_R.

**Table 1.** 5-HT_1A_R binding of selected compounds and reference serotonin.

Compounds	5-HT_1A_ (K_i_ [nM] ± SEM)^a^
**6a**	1.28 ± 0.12
**18a**	1.66 ± 0.18
**Serotonin**	1.64 ± 0.11

^a^K_i_ values were obtained from eight concentrations of the compound, each provided in duplicate.

##### CUMS model assay

**Figure 8.**
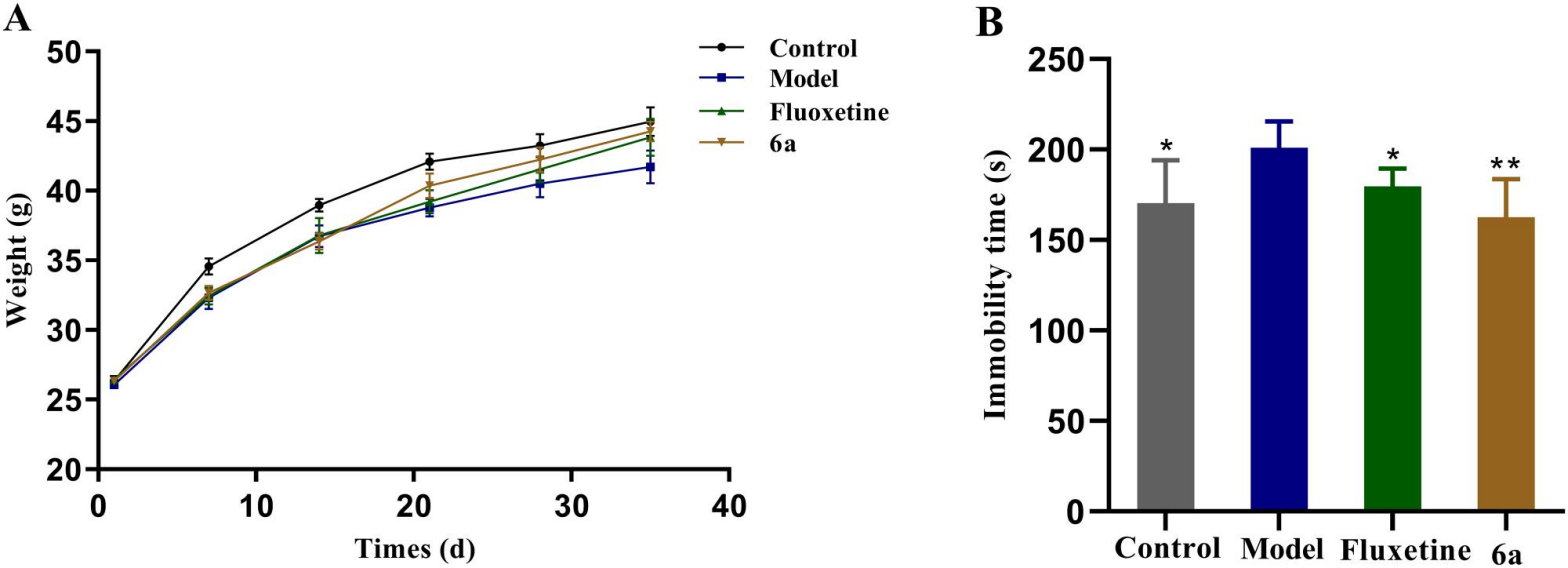
Effect of fluoxetine and compound **6a** on mouse body weights (A) and immobility times (FST, B) in the CUMS model. The values represent the mean ± SEM (*n* = 10). *p < 0.05, **p < 0.01 *vs* control group.

The results of the FST, TST, 5-HT level test, and 5-HT_1A_R affinity test showed that compound **6a** had better antidepressant activity than compound **18a**, thus, the more active compound **6a** was chosen for an in-depth pharmacological activity study. A depression model was prepared using the CUMS method, in which mice exhibited weight loss and reduced voluntary activity. The mice were divided into control, model, fluoxetine, and **6a** groups, and the results of body weight changes in each group on days 7, 14, 21, 28, and 35 are shown in Figure 8(A). From the first day, there was no difference in the body weights of the mice in each group. From the seventh day, the body weights of the mice in the model, fluoxetine, and **6a** groups decreased significantly compared to that of the control group. From the 28^th^ day, the difference in body weight of the mice in the fluoxetine and **6a** groups decreased compared to that of the control group. Compared to the model, the body weights of the mice in the fluoxetine and **6a** groups decreased. After CUMS modelling, FST experiments were performed on all groups of mice, and the experimental results are shown in Figure 8(B). In the control and fluoxetine groups versus model group (*p < 0.05), the mouse swimming immobility time was shortened. In the compound **6a** group *vs* model group (**p < 0.01), the mouse swimming immobility time was significantly shortened.

##### Effects of 6a on hippocampal tissue morphology in mice

**Figure 9.**
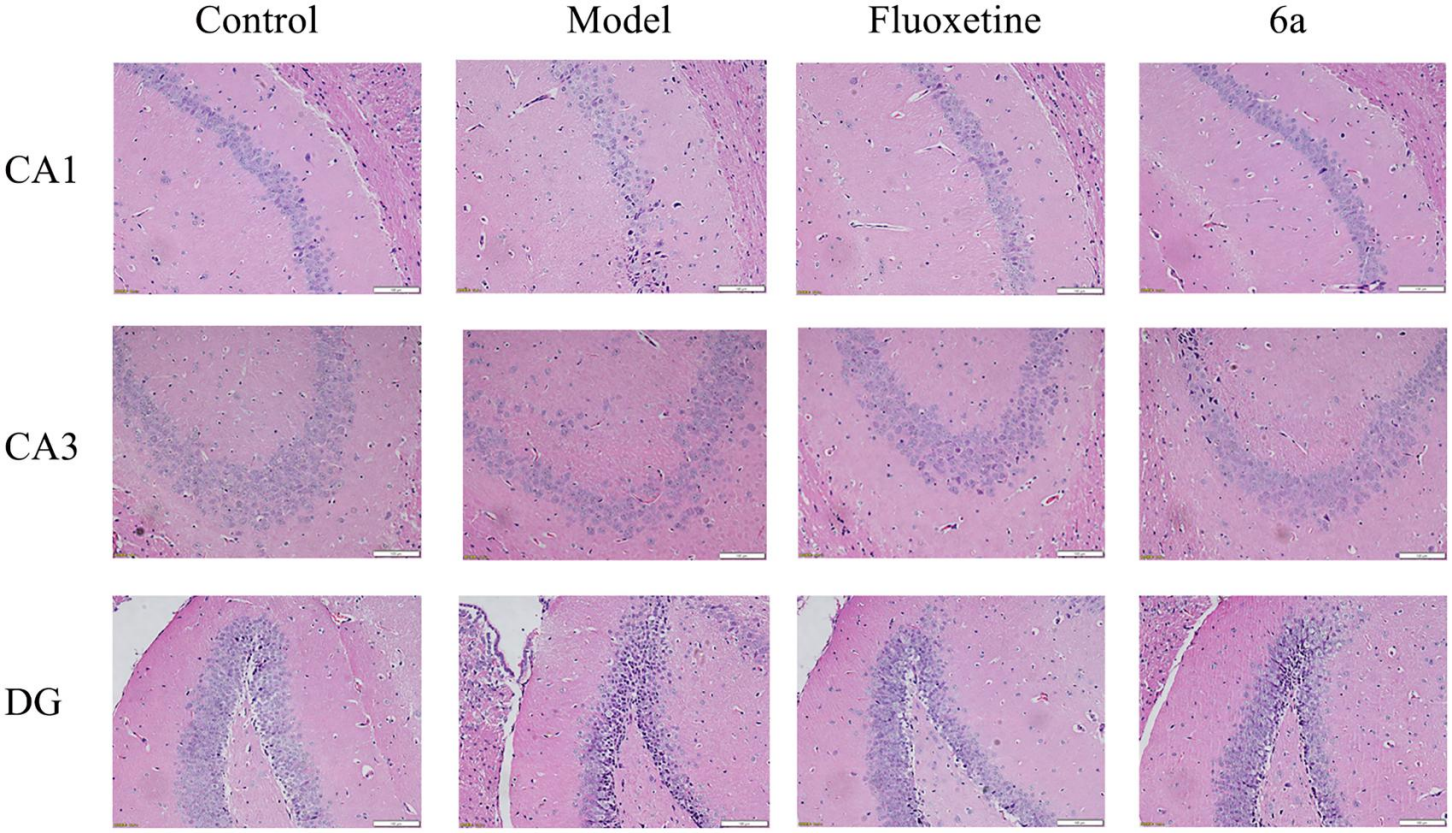
Histomorphological appearance of the CA1, CA3, and DG regions in the mouse hippocampi. H&E staining, original magnification ×200, *scale bar* 100 μm. Control and Model (saline, 10 ml/kg), fluoxetine (20 mg/kg), and compound **6a** (40 mg/kg).

Pathological histological sections of mouse hippocampi in the CUMS model (Figure 9) showed that the cone cells in the control group were neatly arranged and most of the cells had large, round nuclei with obvious and numerous nucleoli. In the model group *vs* control group, the neuronal cells in the mouse hippocampi showed some damage, such as solid and deeply stained cone cells in the CA1 and CA3 regions and more discrete granule cells in the DG regions. In the fluoxetine and **6a** group *vs* model group, the neuronal cells were also well-arranged, and only a few neuronal cells were damaged. The results of this study indicated that the fluoxetine and **6a** groups had a protective effect on the hippocampal tissue morphology of mice in the CUMS depression model and could improve neuronal cell damage.

##### Effects of compound 6a on the protein expression in mouse brain tissue

It has been reported in animal experiments that the total amount of 5-HT_1A_Rs in the hippocampus of a depression model group is less than that of a normal group^17^. PKA and BDNF levels are closely related to the development of depression. It has been shown that depression decreases the expression of PKA, BDNF, and 5-HT_1A_R proteins in the hippocampus of mice, which can lead to neuronal death. The downregulation of the 5-HT_1A_R-PKA-BDNF signalling pathway is one of the pathogenic mechanisms of depression^18^^,^^19^.

**Figure 10.**
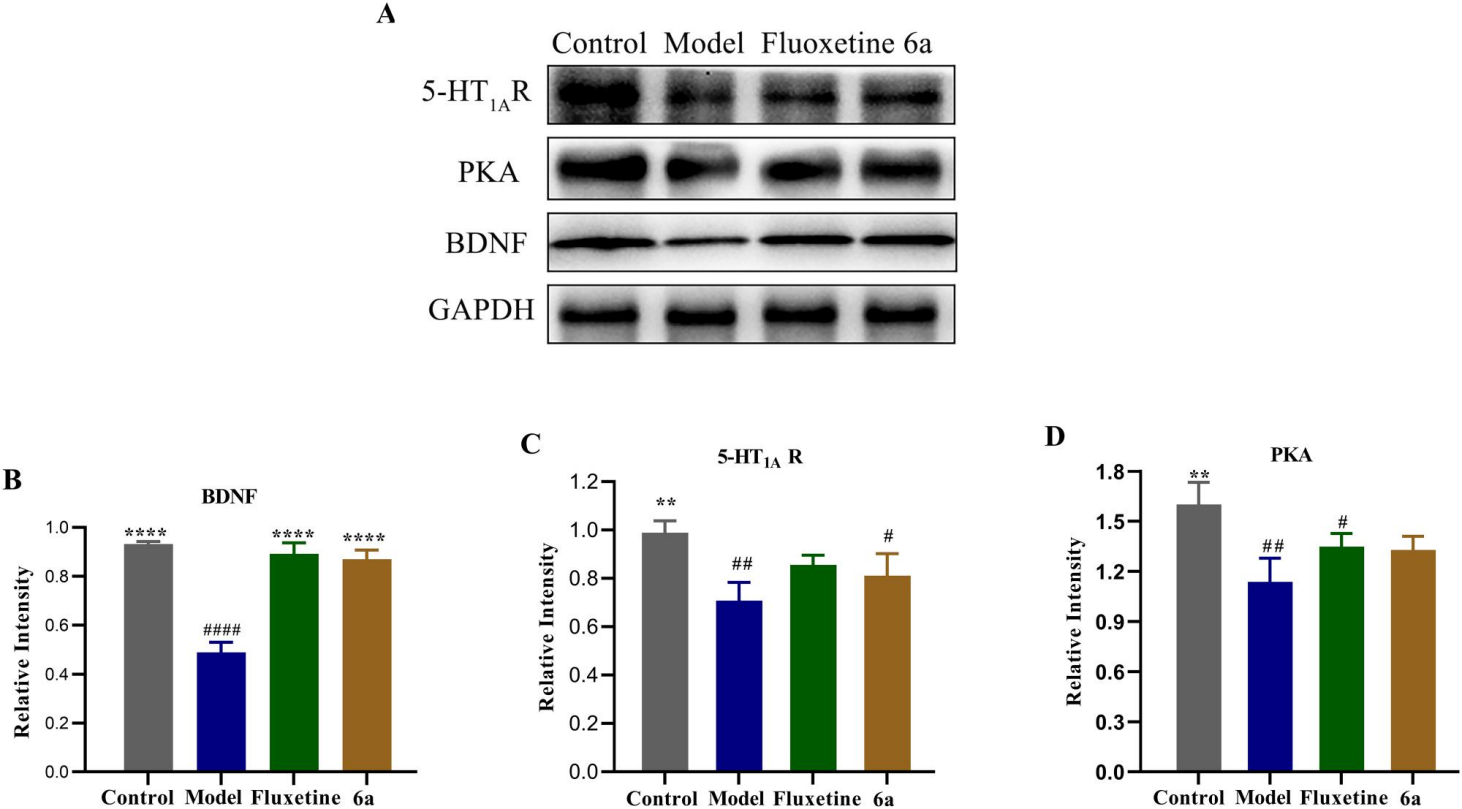
Effects of compound **6a** and fluoxetine on the expression of 5-HT_1A_R, PKA, and BDNF in the brains of mice. (A) Levels of 5-HT_1A_R, PKA, and BDNF were determined by western blot. (B/C/D) Relative levels of 5-HT_1A_R, PKA, and BDNF were determined. Data are expressed as the mean ± SD (*n* = 3). ^#^*p* < 0.1, ^##^ *p* < 0.01, ^####^ *p* < 0.0001 *vs* control; **p < 0.01, ****p < 0.0001 *vs* model.

Changes in the expression of 5-HT_1A_R, PKA, and BDNF in the brain tissue of control, model, fluoxetine, and compound **6a** mice were analysed by western blot in the CUMS depression model. Figure 10 shows that in the model *vs* control group, the protein expression of 5-HT_1A_R (^##^*p* < 0.01), PKA (^##^*p* < 0.01), and BDNF (^####^*p* < 0.0001) was reduced. In the control *vs* model group, the expression of 5-HT_1A_R (**p < 0.01), PKA (**p < 0.01), and BDNF (****p < 0.0001) proteins was increased. In compound **6a** and fluoxetine *vs* model groups, the protein expression of 5-HT_1A_R, PKA, and BDNF (****p < 0.0001) was increased. In summary, the possible mechanism by which compound **6a** improves depression-like behaviour and exerts antidepressant effects in mice is related to the upregulation of 5-HT_1A_R/BDNF/PKA proteins.

**Figure 11.**
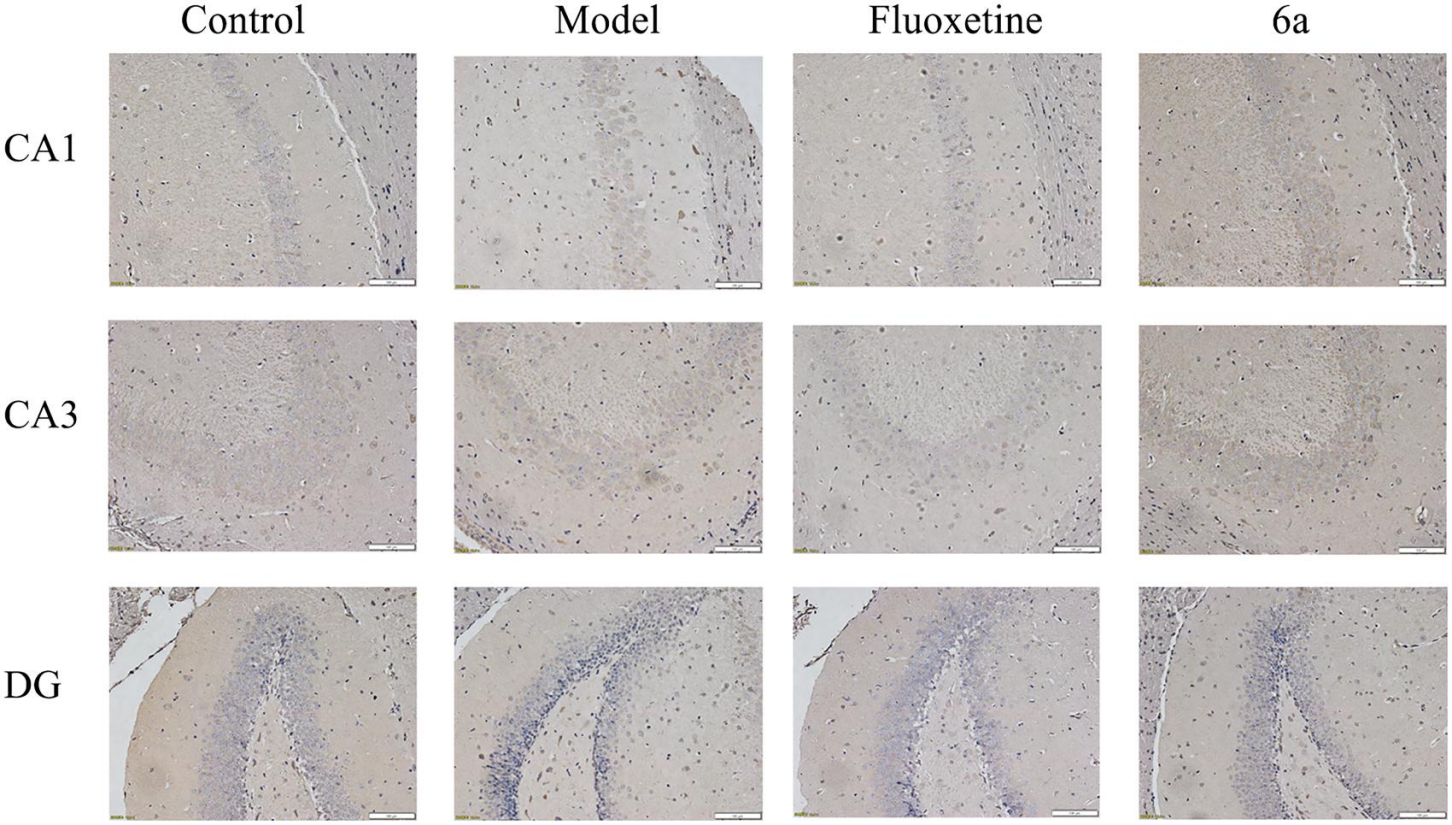
Effects of compound **6a** and fluoxetine on 5-HT_1A_R expression in the hippocampus of CUMS mice. original magnification ×200, *scale bar* 100 μm.

To further confirm the CUMS model-induced hippocampal cell loss, immunohistochemical detection was performed with neuron-specific antibodies. As shown in Figure 11, 5-HT_1A_R expression was reduced in the hippocampus of the model group compared to in that of the control group (CA1 and DG regions). Moreover, there was a significant increase in hippocampal 5-HT_1A_R expression after the intervention of both compound **6a** and fluoxetine compared to that of the model group, especially in the DG regions.

**Figure 12.**
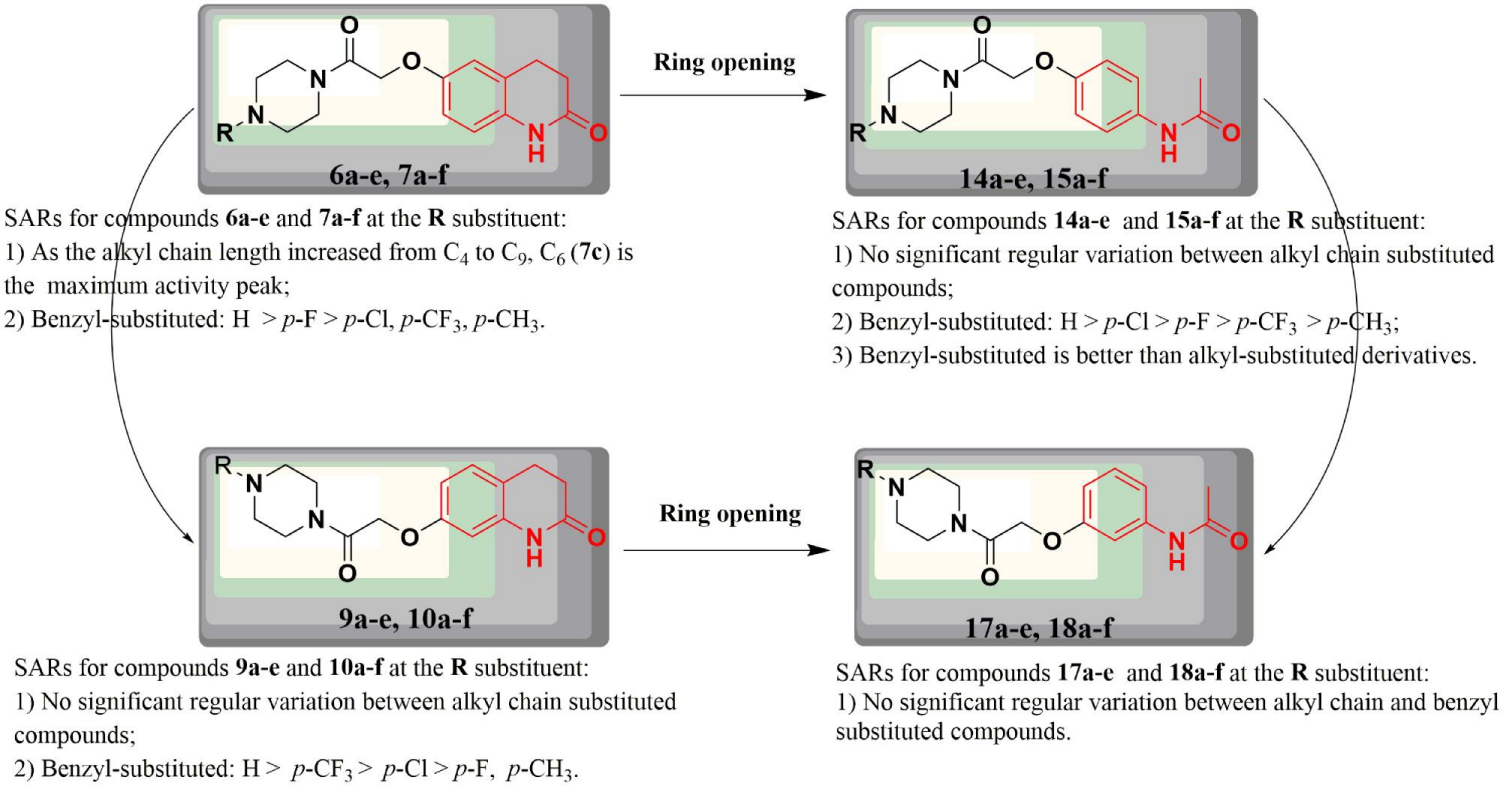
SARs for antidepressant activity of all target compounds at the R substituent.

##### Structure–activity relationships

In this study, a series of target compounds **6a**–**e** and **7a**–**f** were designed and synthesised by combining 3,4-dihydroquinolin-2(1*H*)-one and piperazine structural fragments, and the effect of their structures on antidepressant activity was investigated. In order to assess the effect of the 3,4-dihydroquinolin-2(1*H*)-one linking group at its six and seven positions on activity, the target compounds **9a**–**e** and **10a**–**f** were designed and synthesised. To verify the effect of the 3,4-dihydroquinolin-2(1*H*)-one structural fragment on the antidepressant activity, the C-C double bonds at the three and four positions were cut, and a series of target compounds **14a**–**e**, **15a**–**f**, **17a**–**e**, and **18a**–**f** were designed and synthesised. Ring-opening compounds have increased molecular flexibility compared to compounds **6a**–**e**, **7a**–**f**, **9a**–**e**, and **10a**–**f**, and relationships between ring-opening compounds were explored. In this section, the structure-activity relationships (SARs) of the target compounds were analysed based on the experimental results of the FST (Figure 12).

The pharmacological results for compounds **6a**–**e** and **7a**–**f** showed that among the benzyl-substituted derivatives, the *para* (*p*) positions of electron-withdrawing groups (F, Cl, and CF_3_) and an electron-donating (CH_3_) group on the benzene ring *p* position affected the antidepressant activity of the compound. The order of activity was as follows: H > *p*-F > *p*-Cl, *p*-CF_3_, and *p*-CH_3_, and the best antidepressant activity was exhibited by compound **6a**, where H was not substituted on the benzene ring of benzyl. Among the alkyl chain substituted compounds (C_4_-C_9_-substituted derivatives), the C_6_-substituted compounds had better antidepressant activity than the other compounds, with increasing antidepressant activity as the alkyl chain increased from C_4_ to C_6_ and decreasing antidepressant activity as the alkyl chain increased from C_6_ to C_9_. For SARs for the compounds **9a**–**e** and **10a**–**f**, the order of activity of the benzyl-substituted compounds was as follows: H > *p*-CF_3_ > *p*-Cl > *p*-F, *p*-CH_3_, while the alkyl-substituted compounds showed no regularity. For SARs for the compounds **14a**–**e** and **15a**–**f**, the order of activity of the benzyl-substituted compounds was as follows: *H* >* p*-Cl > *p*-F > *p*-CF_3_ > *p*-CH_3_, while the alkyl-substituted compounds showed no regularity. Overall, benzyl-substituted derivatives antidepressant activity were better than alkyl-substituted derivatives. For SARs for the compounds **17a**–**e** and **18a**–**f**, neither benzyl- nor alkyl-substituted compounds showed regularity.

In summary, compounds **6a**–**e** and **7a**–**f** showed no significant change in antidepressant activity compared to that of **9a**–**e** and **10a**–**f**, meaning that the different positions of the linking groups at the six and seven positions of 3,4-dihydroquinolin-2(1*H*)-one did not significantly improve the antidepressant activity. Compounds **6a**–**e** and **7a**–**f** showed better antidepressant activity compared to their ring-opened derivatives **14a**–**e** and **15a**–**f**, suggesting that the 3,4-dihydroquinolin-2(1*H*)-one structural fragment plays a key role in enhancing antidepressant activity. Compounds **9a**–**e** and **10a**–**f** showed no significant improvement in antidepressant activity compared to their ring-opened derivatives **17a**–**e** and **18a**–**f**, while the ring-opened compounds **17a**–**e** and **18a**–**f** showed better antidepressant activity than **14a**–**e** and **15a**–**f**. Comparing these series of compounds based on activity results, the benzyl-substituted compounds had slightly better antidepressant activity than the alkyl-substituted compounds. Of all the benzyl-substituted compounds, those with no substitution of H on the benzyl ring of the benzyl group had the best antidepressant activity, while those with methyl substitution of H at the *p*-position on the benzyl ring of the benzyl group had the weakest antidepressant activity. Based on the results, it is hypothesised that better antidepressant activity could be exerted for compounds where R is benzyl and the benzene ring H is not substituted, which could provide an important foundation for future research.

##### Docking study

**Figure 13.**
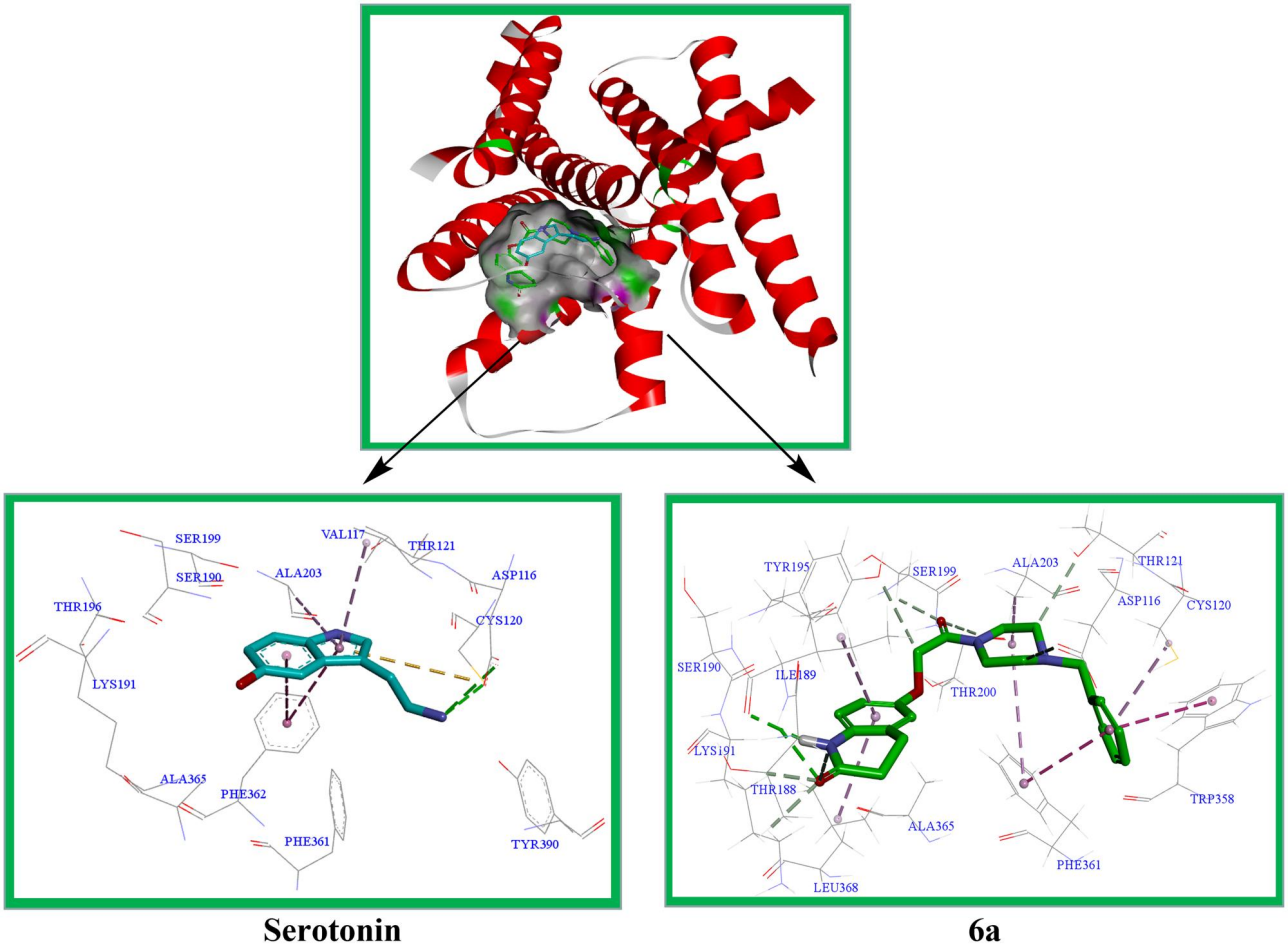
Serotonin and compound **6a** showed interactions with residues at the active site of 5-HT_1A_R, and were superimposed in the serotonin-binding pocket of 5-HT_1A_R.

5-HT_1A_R is an important target for the study of antidepressants. The serotonin-bound 5-HT_1A_R-Gi protein complex was resolved by Xu et al. (PDB Code: 7E2Y)^20^. In this study, compound **6a** was docked to 5-HT_1A_R in a molecular simulation, and the active site was the active region of the protein crystal structure where serotonin (5-HT) was located. Docking was performed using DS 2021 software, and the results are shown in Figure 13.

It has been reported that the amino acids Asp116, Val117, Cys120, Thr121, Thr188, Ile189, Lys191, Thr196, Ser190, Ser199, Ala203, Trp358, Phe361, Phe362, Ala365, and Tyr390 around the serotonin active site are the main amino acids that form the interaction force with the ligand^21^. The docking results showed that serotonin could form hydrogen bonding interactions with Asp116 and Cys120, π-π stacking with Phe362 and Tyr210, and π-alkyl interactions with Val117 and Ala203. The docking results of compound **6a** showed that it could form hydrogen bonding interactions with Ile189; non-classical hydrogen bonding with Asp116, Cys120, Thr121, Thr188, Tyr195, Ser199, and Thr200; π-π stacking with Phe361 and Trp358; and π-alkyl interactions with Cys120, Ala203, and Leu368. The results of the docking experiment showed that compound **6a** formed more interactions with the active site than serotonin.

## Conclusions

In this study, 6-[2–(4-benzylpiperazin-1-yl)-2-oxoethoxy]-3,4-dihydroquinolin-2(1*H*)-one (**6a**) was found to be the most active compound by screening. In the FST and TST models, compound **6a** significantly reduced the immobility time in mice compared to the control group. OFT experiments showed that compound **6a** did not affect spontaneous behaviour in mice. The results of the 5-HT level test showed that compound **6a** significantly increased the 5-HT level in the brains of mice. Compound **6a** has a better affinity for 5-HT_1A_R than serotonin. In the CUMS model, western blot and immunohistochemical analyses showed that compound **6a** significantly increased the expression levels of 5-HT_1A_R, BDNF, and PKA in the hippocampus compared to the model group. This suggested that the antidepressant effect of compound **6a** may be related to the regulation of the 5-HT_1A_R/BDNF/PKA signalling pathway. In the *in silico* study, most of the target compounds had good physicochemical and pharmacokinetic properties, and compound **6a** showed significant interactions with residues present at the active site of 5-HT_1A_R.

## Experimental section

### Chemistry

Structural characterisation of the target compounds was performed by melting point (Mp), ^1^H-NMR, ^13^C-NMR, and HRMS analyses. The solvent used for ^1^H-NMR and ^13^C-NMR tests was DMSO*_d6_* or CDCl_3_, and the letters s, d, t, q, and m represent singlet, doublet, triplet, quartette, and multiplet (degree of peak splitting), respectively. The Mp of the target compounds was measured using an X-4 binocular microscope (Shanghai, China). The ^1^H-NMR and ^13^C-NMR of the target compounds were measured using a Bruker AV 500 NMR spectrometer (Bruker, Switzerland). The HRMS of the compounds was measured using a high-resolution mass spectrometer (Bruker Daltonik, Germany). The raw materials, solvents, and reagents used in the synthesis of the compounds were mainly purchased from Sigma–Aldrich, Aladdin, Shenyang Chemical Reagent Factory, and China Reagent Network.

#### Synthesis of Intermediate 2

A clean and dry 100-ml round-bottom flask was prepared. Then, 1-boc-piperazine (10.0 mmol) and triethylamine (20.0 mmol) were weighed and added to the flask. A suitable-sized stirring magnet was placed in the flask, and 30 ml of CH_2_Cl_2_ was added as the solvent for the reaction. The flask was then placed in a low-temperature reaction apparatus and the temperature was lowered to −10 °C with stirring. Chloroacetyl chloride (15.0 mmol) and 3 ml of CH_2_Cl_2_ were added to a suitable volume in a constant pressure funnel and added slowly dropwise to the reaction flask. After dropwise addition, the temperature was raised to room temperature and the reaction was continued for 4 h. The reaction was followed by TLC (CH_2_Cl_2_: CH_3_OH = 50:1) and when the reaction was completed, the CH_2_Cl_2_ was removed by a vacuum decompression device, leaving a grey solid. Next, 30 ml of water was added to the reaction flask and stirred for 15 min at room temperature. This was then filtered with a filtration device to obtain the crude product Intermediate 2, which could be used directly in the next reaction step without purification.

#### Synthesis of Intermediate 4

A clean and dry 100-ml round-bottom flask was prepared. Then, 3,4-dihydro-6-hydroxy-2(1*H*)-quinolinone (10.0 mmol) and K_2_CO_3_ (12.0 mmol) were weighed and added to the flask. A suitable-sized stirring magnet was placed in the flask and 50 ml of acetone was added as the solvent for the reaction. The round-bottom flask was placed in a heated reaction apparatus, the temperature was raised to 60 °C with stirring, and the reaction was carried out for 0.5 h. Intermediate **2** (10.0 mmol) was added to the reaction flask and the reaction was continued for 7 h. The crude product Intermediate **4** was obtained by removing the acetone under reduced pressure, washing the solid with water, and filtering.

#### Synthesis of intermediate 5

A clean and dry 100-ml round-bottom flask was prepared, Intermediate **4** (10.0 mmol) and trifluoroacetic acid (1 ml) were added to the flask, a suitable-sized stirring magnet was placed in the flask, and 50 ml of CH_2_Cl_2_ was added as the solvent for the reaction. The mixture was stirred and reacted at room temperature for 12 h. The crude product Intermediate **5** was obtained by removing the CH_2_Cl_2_ under reduced pressure without purification.

#### Synthesis of target compounds 6a–e and 7a–f

A clean and dry 100-ml round-bottom flask was prepared, Intermediate **5** (10.0 mmol) and K_2_CO_3_ (11.0 mmol) were weighed and added to the flask, a suitable-sized stirring magnet was placed in the flask, and 50 ml of acetone was added as the solvent for the reaction. The round-bottom flask was placed in a heated reaction apparatus, the temperature was raised to 65 °C with stirring, and the reaction was carried out for 0.5 h. Different substituted bromine substituents (10.0 mmol) were added to the reaction flask and the reaction continued for 8–10 h. The target compounds **6a–e** and **7a–f** were obtained by removing the acetone under reduced pressure, washing the solid with water, filtering, and undergoing column chromatography (CH_2_Cl_2:_ CH_3_OH = 120: 1).

##### 6–(2-(4-Benzylpiperazin-1-yl)-2-oxoethoxy)-3,4-dihydroquinolin-2(1H)-one (6a)

Yield: 67%. Mp: 172–173 °C. ^1^H-NMR (500 MHz, CDCl_3_, ppm) *δ*: 8.63 (*s*, 1H, CONH), 6.71–7.35 (*m*, 8H, Ar-H), 4.64 (*s*, 2H, -OCH_2_), 3.54–3.65 (*m*, 4H, Piperazine-H), 3.48 (*s*, 2H, -NCH_2_), 2.91–2.94 (*t*, *J* = 7.75 Hz, 2H, NHCOCH_2_), 2.59–2.62 (*t*, *J* = 7.50 Hz, 2H, NHCOCH_2_CH_2_), 2.46 (*s*, 4H, Piperazine-H). ^13^C NMR (125 MHz, CDCl_3_) *δ*: 171.43, 166.35, 153.79, 131.68, 129.18, 128.41, 127.46, 125.18, 116.27, 114.65, 113.42, 68.00, 62.74, 53.03, 52.58, 45.18, 41.99, 30.53, 25.66. ESI-HRMS calcd for C_22_H_26_N_3_O_3_^+^ ([M + H]^+^): 380.1969; found: 380.1980.

##### 6–(2-(4–(4-Fluorobenzyl)piperazin-1-yl)-2-oxoethoxy)-3,4-dihydroquinolin-2(1H)-one (6b)

Yield: 57%. Mp: 184–185 °C. ^1^H-NMR (500 MHz, CDCl_3_, ppm) *δ*: 8.63 (*s*, 1H, CONH), 6.71–7.26 (*m*, 7H, Ar-H), 4.64 (*s*, 2H, -OCH_2_), 3.57–3.63 (*m*, 4H, Piperazine-H), 3.48 (*s*, 2H, -NCH_2_), 2.91–2.94 (*t*, *J* = 7.50 Hz, 2H, NHCOCH_2_), 2.59–2.62 (*t*, *J* = 7.75 Hz, 2H, NHCOCH_2_CH_2_), 2.43 (*s*, 4H, Piperazine-H). ^13^C NMR (125 MHz, CDCl_3_) *δ*: 171.45, 166.37, 153.80, 131.70, 130.59, 125.19, 116.27, 115.30, 115.13, 114.65, 113.42, 68.01, 61.94, 52.99, 52.55, 45.24, 42.05, 30.53, 25.67. ESI-HRMS calcd for C_22_H_25_FN_3_O_3_^+^ ([M + H]^+^): 398.1874; found: 398.1882.

##### 6–(2-(4–(4-Chlorobenzyl)piperazin-1-yl)-2-oxoethoxy)-3,4-dihydroquinolin-2(1H)-one (6c)

Yield: 49%. Mp: 192–193 °C. ^1^H-NMR (500 MHz, CDCl_3_, ppm) *δ*: 8.73 (*s*, 1H, CONH), 6.74–7.30 (*m*, 7H, Ar-H), 4.65 (*s*, 2H, -OCH_2_), 3.58–3.64 (*m*, 4H, Piperazine-H), 3.48 (*s*, 2H, -NCH_2_), 2.91–2.94 (*t*, *J* = 7.50 Hz, 2H, NHCOCH_2_), 2.59–2.62 (*t*, *J* = 7.50 Hz, 2H, NHCOCH_2_CH_2_), 2.43 (*s*, 4H, Piperazine-H). ^13^C NMR (125 MHz, CDCl_3_) *δ*: 171.49, 166.38, 153.78, 131.71, 130.35, 128.54, 125.18, 116.30, 114.63, 113.42, 68.01, 61.96, 53.02, 52.58, 45.21, 42.03, 30.53, 25.66. ESI-HRMS calcd for C_22_H_25_ClN_3_O_3_^+^ ([M + H]^+^): 414.1579; found: 414.1584.

##### 6–(2-Oxo-2–(4-(4-(trifluoromethyl)benzyl)piperazin-1-yl)ethoxy)-3,4-dihydroquinolin-2(1H)-one (6d)

Yield: 62%. Mp: 172–173 °C. ^1^H-NMR (500 MHz, CDCl_3_, ppm) *δ*: 8.56 (*s*, 1H, CONH), 6.73–7.59 (*m*, 7H, Ar-H), 4.65 (*s*, 2H, -OCH_2_), 3.57–3.65 (*m*, 4H, Piperazine-H), 3.48 (*s*, 2H, -NCH_2_), 2.91–2.94 (*t*, *J* = 7.50 Hz, 2H, NHCOCH_2_), 2.59–2.62 (*t*, *J* = 7.50 Hz, 2H, NHCOCH_2_CH_2_), 2.44–2.45 (*m*, 4H, Piperazine-H). ^13^C NMR (125 MHz, CDCl_3_) *δ*: 171.41, 166.41, 153.80, 131.70, 129.50, 129.16, 125.35, 125.33, 125.22, 116.27, 114.66, 113.42, 68.03, 62.20, 53.16, 52.72, 45.26, 42.06, 30.53, 25.67. ESI-HRMS calcd for C_23_H_25_F_3_N_3_O_3_^+^ ([M + H]^+^): 448.1843; found: 448.1848.

##### 6–(2-(4–(4-Methylbenzyl)piperazin-1-yl)-2-oxoethoxy)-3,4-dihydroquinolin-2(1H)-one (6e)

Yield: 55%. Mp: 110–111 °C. ^1^H-NMR (500 MHz, CDCl_3_, ppm) *δ*: 8.61 (*s*, 1H, CONH), 6.73–7.21 (*m*, 7H, Ar-H), 4.64 (*s*, 2H, -OCH_2_), 3.59–3.65 (*m*, 4H, Piperazine-H), 3.48 (*s*, 2H, -NCH_2_), 2.91–2.94 (*t*, *J* = 7.50 Hz, 2H, NHCOCH_2_), 2.58–2.61 (*t*, *J* = 7.50 Hz, 2H, NHCOCH_2_CH_2_), 2.46 (*s*, 4H, Piperazine-H), 2.34 (*s*, 3H, -CH_3_). ^13^C NMR (125 MHz, CDCl_3_) *δ*: 171.48, 166.37, 153.80, 137.22, 131.68, 129.50, 129.23, 129.10, 125.20, 116.29, 114.67, 113.43, 67.97, 62.46, 52.97, 52.49, 45.12, 41.94, 30.52, 25.65, 21.12. ESI-HRMS calcd for C_23_H_28_N_3_O_3_^+^ ([M + H]^+^): 394.2125; found: 394.2137

##### 6–(2-(4-Butylpiperazin-1-yl)-2-oxoethoxy)-3,4-dihydroquinolin-2(1H)-one (7a)

Yield: 38%. Mp: 78–79 °C. ^1^H-NMR (500 MHz, CDCl_3_, ppm) *δ*: 8.75 (*s*, 1H, CONH), 6.77–6.78 (*m*, 3H, Ar-H), 4.65 (*s*, 2H, -OCH_2_), 3.58–3.65 (*m*, 4H, Piperazine-H), 2.91–2.94 (*t*, *J* = 7.50 Hz, 2H, NHCOCH_2_), 2.59–2.62 (*t*, *J* = 7.50 Hz, 2H, NHCOCH_2_CH_2_), 2.43–2.46 (*m*, 4H, Piperazine-H), 1.31–2.37 (*m*, 6H, (-CH_2_-)_3_), 0.90–0.93 (*t*, 3H, *J* = 7.50 Hz, -CH_3_). ^13^C NMR (125 MHz, CDCl_3_) *δ*: 171.56, 166.36, 153.84, 131.71, 125.20, 116.32, 114.68, 113.44, 67.97, 58.25, 53.37, 52.75, 45.20, 42.02, 30.53, 28.80, 25.66, 20.63, 14.00. ESI-HRMS calcd for C_19_H_28_N_3_O_3_^+^ ([M + H]^+^): 346.2125; found: 334.1215.

##### 6–(2-Oxo-2–(4-pentylpiperazin-1-yl)ethoxy)-3,4-dihydroquinolin-2(1H)-one (7b)

Yield: 47%. Mp: 104–105 °C. ^1^H-NMR (500 MHz, CDCl_3_, ppm) *δ*: 8.77 (s, 1H, CONH), 6.74–6.78 (m, 3H, Ar-H), 4.65 (s, 2H, -OCH_2_), 3.57–3.66 (*m*, 4H, Piperazine-H), 2.91–2.94 (*t*, *J* = 7.50 Hz, 2H, NHCOCH_2_), 2.59–2.62 (*t*, *J* = 7.50 Hz, 2H, NHCOCH_2_CH_2_), 2.41–2.46 (*m*, 4H, Piperazine-H), 1.26–2.35 (*m*, 8H, (-CH_2_-)_4_), 0.88–0.91 (*t*, 3H, *J* = 7.00 Hz, -CH_3_). ^13^C NMR (125 MHz, CDCl_3_) *δ*: 171.54, 166.32, 153.83, 131.70, 125.17, 116.30, 114.67, 113.43, 67.97, 58.55, 53.38, 52.76, 45.24, 42.05, 30.53, 29.64, 26.41, 25.65, 22.57, 14.02. ESI-HRMS calcd for C_20_H_30_N_3_O_3_^+^ ([M + H]^+^): 360.2282; found: 360.2289.

##### 6–(2-(4-Hexylpiperazin-1-yl)-2-oxoethoxy)-3,4-dihydroquinolin-2(1H)-one (7c)

Yield: 66%. Mp: 87–88 °C. ^1^H-NMR (500 MHz, CDCl_3_, ppm) *δ*: 8.84 (s, 1H, CONH), 6.75–6.78 (*m*, 3H, Ar-H), 4.65 (*s*, 2H, -OCH_2_), 3.57–3.65 (*m*, 4H, Piperazine-H), 2.91–2.94 (*t*, *J* = 7.50 Hz, 2H, NHCOCH_2_), 2.59–2.62 (t, *J* = 7.50 Hz, 2H, NHCOCH_2_CH_2_), 2.41–2.45 (*m*, 4H, Piperazine-H), 1.29–2.35 (*m*, 10H, (-CH_2_-)_5_), 0.87–0.90 (*t*, 3H, *J* = 6.75 Hz, -CH_3_). ^13^C NMR (125 MHz, CDCl_3_) *δ*: 171.56, 166.32, 153.83, 131.71, 125.16, 116.31, 114.66, 113.43, 67.96, 58.58, 53.39, 52.77, 45.26, 42.08, 31.73, 30.52, 27.14, 26.71, 25.65, 22.59, 14.03. ESI-HRMS calcd for C_21_H_32_N_3_O_3_^+^ ([M + H]^+^): 374.2438; found: 374.2447.

##### 6–(2-(4-Heptylpiperazin-1-yl)-2-oxoethoxy)-3,4-dihydroquinolin-2(1H)-one (7d)

Yield: 54%. Mp: 80–81 °C. ^1^H-NMR (500 MHz, CDCl_3_, ppm) *δ*: 8.74 (*s*, 1H, CONH), 6.74–6.78 (*m*, 3H, Ar-H), 4.65 (*s*, 2H, -OCH_2_), 3.58–3.65 (*m*, 4H, Piperazine-H), 2.91–2.94 (*t*, *J* = 7.50 Hz, 2H, NHCOCH_2_), 2.59–2.62 (*t*, *J* = 7.50 Hz, 2H, NHCOCH_2_CH_2_), 2.43–2.46 (*m*, 4H, Piperazine-H), 1.29–2.36 (*m*, 12H, (-CH_2_-)_6_), 0.86–0.89 (t, 3H, *J* = 7.00 Hz, -CH_3_). ^13^C NMR (125 MHz, CDCl_3_) *δ*: 171.53, 166.34, 153.83, 131.71, 125.19, 116.31, 114.68, 113.44, 67.97, 58.58, 53.37, 52.75, 45.20, 42.01, 31.82, 30.53, 29.48, 29.24, 27.46, 26.68, 25.66, 22.65, 14.10. ESI-HRMS calcd for C_22_H_34_N_3_O_3_^+^ ([M + H]^+^): 388.2595; found: 388.2603.

##### 6–(2-(4-Octylpiperazin-1-yl)-2-oxoethoxy)-3,4-dihydroquinolin-2(1H)-one (7e)

Yield: 51%. Mp: 80–81 °C. ^1^H-NMR (500 MHz, CDCl_3_, ppm) *δ*: 8.77 (*s*, 1H, CONH), 6.74–6.78 (*m*, 3H, Ar-H), 4.65 (*s*, 2H, -OCH_2_), 3.58–3.65 (*m*, 4H, Piperazine-H), 2.91–2.94 (*t*, *J* = 7.50 Hz, 2H, NHCOCH_2_), 2.59–2.62 (*t*, *J* = 7.50 Hz, 2H, NHCOCH_2_CH_2_), 2.43–2.45 (*m*, 4H, Piperazine-H), 1.27–2.36 (*m*, 14H, (-CH_2_-)_7_), 0.87–0.89 (*t*, 3H, *J* = 6.75 Hz, -CH_3_). ^13^C NMR (125 MHz, CDCl_3_) *δ*: 171.54, 166.33, 153.83, 131.72, 125.18, 116.31, 114.68, 113.44, 67.98, 58.58, 53.38, 52.76, 45.22, 42.03, 31.78, 30.54, 29.19, 27.42, 26.70, 25.67, 22.61, 14.08. ESI-HRMS calcd for C_23_H_36_N_3_O_3_^+^ ([M + H]^+^): 402.2751; found: 402.2757.

##### 6–(2-(4-Nonylpiperazin-1-yl)-2-oxoethoxy)-3,4-dihydroquinolin-2(1H)-one (7f)

Yield: 62%. Mp: 81–82 °C. ^1^H-NMR (500 MHz, CDCl_3_, ppm) *δ*: 8.68 (*s*, 1H, CONH), 6.74–6.78 (*m*, 3H, Ar-H), 4.65 (*s*, 2H, -OCH_2_), 3.59–3.65 (*m*, 4H, Piperazine-H), 2.91–2.94 (*t*, *J* = 7.50 Hz, 2H, NHCOCH_2_), 2.59–2.62 (*t*, *J* = 7.50 Hz, 2H, NHCOCH_2_CH_2_), 2.43–2.46 (*m*, 4H, Piperazine-H), 1.26–2.36 (*m*, 16H, (-CH_2_-)_8_), 0.86–0.89 (*t*, 3H, *J* = 7.00 Hz, -CH_3_). ^13^C NMR (125 MHz, CDCl_3_) *δ*: 171.49, 166.32, 153.84, 131.70, 125.19, 116.29, 114.68, 113.44, 67.99, 58.58, 53.45, 53.38, 52.76, 45.21, 42.02, 31.87, 30.54, 29.53, 29.27, 27.45, 26.69, 25.67, 22.67, 14.11. ESI-HRMS calcd for C_24_H_38_N_3_O_3_^+^ ([M + H]^+^): 416.2908; found: 416.2917.

#### Synthesis of target compounds 9a–e, 10a–f, 14a–e, 15a–f, 17a–e, and 18a–f

The target compounds **9a–e**, **10a–f**, **14a–e**, **15a–f**, **17a–e**, and **18a–f** and their intermediates were synthesised in a similar manner to the target compounds **6a–e** and **7a–f** and their intermediates **4** and **5**.

##### 7–(2-(4-Benzylpiperazin-1-yl)-2-oxoethoxy)-3,4-dihydroquinolin-2(1H)-one (9a)

Yield: 39%. Mp: 148–149 °C. ^1^H-NMR (500 MHz, CDCl_3_, ppm) *δ*: 8.34 (*s*, 1H, CONH), 6.34–7.25 (*m*, 8H, Ar-H), 4.58 (*s*, 2H, -OCH_2_), 3.48–3.57 (*m*, 4H, Piperazine-H), 3.44 (*s*, 2H, -NCH_2_), 2.80–2.83 (*t*, *J* = 7.50 Hz, 2H, NHCOCH_2_), 2.52–2.55 (*t*, *J* = 7.50 Hz, 2H, NHCOCH_2_CH_2_), 2.37–2.38 (*m*, 4H, Piperazine-H). ^13^C NMR (125 MHz, CDCl_3_) *δ*: 170.62, 165.21, 156.47, 137.40, 128.08, 127.78, 127.35, 126.33, 115.83, 107.66, 101.44, 66.73, 61.78, 52.07, 51.62, 44.30, 41.12, 29.97, 23.61. ESI-HRMS calcd for C_22_H_26_N_3_O_3_^+^ ([M + H]^+^): 380.1969; found: 380.1973.

##### 7–(2-(4–(4-Fluorobenzyl)piperazin-1-yl)-2-oxoethoxy)-3,4-dihydroquinolin-2(1H)-one (9b)

Yield: 47%. Mp: 163–164 °C. ^1^H-NMR (500 MHz, CDCl_3_, ppm) *δ*: 8.45 (*s*, 1H, CONH), 6.36–7.21 (*m*, 7H, Ar-H), 4.58 (*s*, 2H, -OCH_2_), 3.48–3.57 (*m*, 4H, Piperazine-H), 3.40 (*s*, 2H, -NCH_2_), 2.80–2.83 (*t*, *J* = 7.50 Hz, 2H, NHCOCH_2_), 2.51–2.54 (*t*, *J* = 7.50 Hz, 2H, NHCOCH_2_CH_2_), 2.34–2.35 (*m*, 4H, Piperazine-H). ^13^C NMR (125 MHz, CDCl_3_) *δ*: 171.72, 166.26, 163.10, 161.15, 157.47, 138.44, 133.26, 130.55, 130.49, 128.79, 116.86, 115.26, 115.09, 108.68, 102.46, 67.75, 61.96, 53.02, 52.57, 45.32, 42.14, 30.98, 24.62. ESI-HRMS calcd for C_22_H_25_FN_3_O_3_^+^ ([M + H]^+^): 398.1874; found: 398.1879.

##### 7–(2-(4–(4-Chlorobenzyl)piperazin-1-yl)-2-oxoethoxy)-3,4-dihydroquinolin-2(1H)-one (9c)

Yield: 51%. Mp: 169–170 °C. ^1^H-NMR (500 MHz, CDCl_3_, ppm) *δ*: 8.60 (*s*, 1H, CONH), 6.43–7.31 (*m*, 7H, Ar-H), 4.65 (*s*, 2H, -OCH_2_), 3.55–3.63 (*m*, 4H, Piperazine-H), 3.47 (*s*, 2H, -NCH_2_), 2.87–2.90 (*t*, *J* = 7.50 Hz, 2H, NHCOCH_2_), 2.58–2.61 (*t*, *J* = 7.50 Hz, 2H, NHCOCH_2_CH_2_), 2.38–2.42 (*m*, 4H, Piperazine-H). ^13^C NMR (125 MHz, CDCl_3_) *δ*: 171.75, 166.28, 157.45, 138.45, 136.14, 133.05, 130.30, 128.78, 128.52, 116.86, 108.68, 102.46, 67.75, 61.99, 53.05, 52.61, 45.31, 42.13, 30.98, 24.62. ESI-HRMS calcd for C_22_H_25_ClN_3_O_3_^+^ ([M + H]^+^): 414.1579; found: 414.1585.

##### 7–(2-Oxo-2–(4-(4-(trifluoromethyl)benzyl)piperazin-1-yl)ethoxy)-3,4-dihydroquinolin-2(1H)-one (9d)

Yield: 54%. Mp: 163–164 °C. ^1^H-NMR (500 MHz, CDCl_3_, ppm) *δ*: 8.28 (*s*, 1H, CONH), 6.35–7.51 (*m*, 7H, Ar-H), 4.59 (*s*, 2H, -OCH_2_), 3.51–3.57 (*m*, 4H, Piperazine-H), 3.49 (*s*, 2H, -NCH_2_), 2.80–2.83 (*t*, *J* = 7.50 Hz, 2H, NHCOCH_2_), 2.52–2.55 (*t*, *J* = 7.50 Hz, 2H, NHCOCH_2_CH_2_), 2.37–2.38 (*m*, 4H, Piperazine-H). ^13^C NMR (125 MHz, CDCl_3_) *δ*: 171.62, 166.29, 157.45, 141.90, 138.41, 129.78, 129.52, 129.12, 128.82, 125.34, 125.31, 123.09, 116.90, 108.68, 102.41, 67.78, 62.19, 53.15, 52.71, 45.31, 42.11, 30.97, 24.61. ESI-HRMS calcd for C_23_H_25_F_3_N_3_O_3_^+^ ([M + H]^+^): 448.1843; found: 448.1849.

##### 7–(2-(4–(4-Methylbenzyl)piperazin-1-yl)-2-oxoethoxy)-3,4-dihydroquinolin-2(1H)-one (9e)

Yield: 57%. Mp: 188–189 °C. ^1^H-NMR (500 MHz, CDCl_3_, ppm) *δ*: 8.41 (*s*, 1H, CONH), 6.41–7.19 (*m*, 7H, Ar-H), 4.65 (*s*, 2H, -OCH_2_), 3.54–3.63 (*m*, 4H, Piperazine-H), 3.48 (*s*, 2H, -NCH_2_), 2.87–2.90 (*t*, *J* = 7.50 Hz, 2H, NHCOCH_2_), 2.59–2.62 (*t*, *J* = 7.50 Hz, 2H, NHCOCH_2_CH_2_), 2.41–2.44 (*m*, 4H, Piperazine-H), 2.34 (*s*, 1H, -CH_3_). ^13^C NMR (125 MHz, CDCl_3_) *δ*: 171.66, 166.22, 157.48, 138.40, 136.99, 134.38, 129.09, 129.04, 128.79, 116.84, 108.69, 102.44, 67.72, 62.54, 53.06, 52.58, 50.78, 45.32, 42.15, 30.99, 24.62, 21.11. ESI-HRMS calcd for C_23_H_28_N_3_O_3_^+^ ([M + H]^+^): 394.2125; found: 394.2129.

##### 7–(2-(4-Butylpiperazin-1-yl)-2-oxoethoxy)-3,4-dihydroquinolin-2(1H)-one (10a)

Yield: 62%. Mp: 122–123 °C. ^1^H-NMR (500 MHz, CDCl_3_, ppm) *δ*: 8.33 (*s*, 1H, CONH), 6.36–6.98 (*m*, 3H, Ar-H), 4.59 (*s*, 2H, -OCH_2_), 3.50–3.57 (*m*, 4H, Piperazine-H), 2.80–2.83 (*t*, *J* = 7.50 Hz, 2H, NHCOCH_2_), 2.52–2.55 (*t*, *J* = 7.50 Hz, 2H, NHCOCH_2_CH_2_), 2.34–2.38 (*m*, 4H, Piperazine-H), 1.23–2.29 (*m*, 6H, (-CH_2_-)_3_), 0.83–0.86 (t, 3H, *J* = 7.50 Hz, -CH_3_). ^13^C NMR (125 MHz, CDCl_3_) *δ*: 170.64, 165.19, 156.47, 137.40, 127.79, 115.84, 107.69, 101.42, 66.71, 57.22, 52.36, 51.74, 49.73, 44.25, 41.08, 29.96, 27.83, 23.60, 19.61, 12.97. ESI-HRMS calcd for C_19_H_28_N_3_O_3_^+^ ([M + H]^+^): 346.2125; found: 346.2139.

##### 7–(2-Oxo-2–(4-pentylpiperazin-1-yl)ethoxy)-3,4-dihydroquinolin-2(1H)-one (10b)

Yield: 64%. Mp: 116–117 °C. ^1^H-NMR (500 MHz, CDCl_3_, ppm) *δ*: 8.35 (*s*, 1H, CONH), 6.36–6.98 (*m*, 3H, Ar-H), 4.59 (*s*, 2H, -OCH_2_), 3.51–3.57 (*m*, 4H, Piperazine-H), 2.80–2.83 (*t*, *J* = 7.50 Hz, 2H, NHCOCH_2_), 2.52–2.55 (*t*, *J* = 7.50 Hz, 2H, NHCOCH_2_CH_2_), 2.35–2.37 (*m*, 4H, Piperazine-H), 1.19–2.28 (*m*, 8H, (-CH_2_-)_4_), 0.81–0.84 (*t*, 3H, *J* = 7.50 Hz, -CH_3_). ^13^C NMR (125 MHz, CDCl_3_) *δ*: 170.64, 165.18, 156.47, 137.41, 127.77, 115.83, 107.67, 101.45, 66.71, 57.52, 52.36, 51.75, 44.27, 41.09, 29.97, 28.63, 25.41, 23.60, 21.56, 13.01. ESI-HRMS calcd for C_20_H_30_N_3_O_3_^+^ ([M + H]^+^): 360.2282; found: 360.2294.

##### 7–(2-(4-Hexylpiperazin-1-yl)-2-oxoethoxy)-3,4-dihydroquinolin-2(1H)-one (10c)

Yield: 69%. Mp: 118–119 °C. ^1^H-NMR (500 MHz, CDCl_3_, ppm) *δ*: 8.57 (*s*, 1H, CONH), 6.44–7.05 (*m*, 3H, Ar-H), 4.67 (*s*, 2H, -OCH_2_), 3.57–3.65 (*m*, 4H, Piperazine-H), 2.87–2.90 (*t*, *J* = 7.50 Hz, 2H, NHCOCH_2_), 2.59–2.62 (*t*, *J* = 7.50 Hz, 2H, NHCOCH_2_CH_2_), 2.42–2.45 (*m*, 4H, Piperazine-H), 1.29–2.35 (*m*, 10H, (-CH_2_-)_5_), 0.87–0.90 (*t*, 3H, *J* = 7.50 Hz, -CH_3_). ^13^C NMR (125 MHz, CDCl_3_) *δ*: 171.75, 166.21, 157.48, 138.44, 128.77, 116.84, 108.69, 102.46, 67.71, 58.55, 53.36, 52.75, 45.26, 42.09, 31.73, 30.97, 27.13, 26.68, 24.61, 22.58, 14.03. ESI-HRMS calcd for C_21_H_32_N_3_O_3_^+^ ([M + H]^+^): 374.2438; found: 374.2443.

##### 7–(2-(4-Heptylpiperazin-1-yl)-2-oxoethoxy)-3,4-dihydroquinolin-2(1H)-one (10d)

Yield: 55%. Mp: 108–109 °C. ^1^H-NMR (500 MHz, CDCl_3_, ppm) *δ*: 8.11 (*s*, 1H, CONH), 6.41–7.06 (*m*, 3H, Ar-H), 4.66 (*s*, 2H, -OCH_2_), 3.57–3.65 (*m*, 4H, Piperazine-H), 2.88–2.91 (*t*, *J* = 7.50 Hz, 2H, NHCOCH_2_), 2.59–2.62 (*t*, *J* = 7.50 Hz, 2H, NHCOCH_2_CH_2_), 2.43–2.45 (*m*, 4H, Piperazine-H), 1.28–2.35 (*m*, 12H, (-CH_2_-)_6_), 0.87–0.90 (*t*, 3H, *J* = 7.50 Hz, -CH_3_). ^13^C NMR (125 MHz, CDCl_3_) *δ*: 171.47, 166.16, 157.49, 138.36, 128.84, 116.87, 108.69, 102.39, 67.74, 58.55, 53.37, 52.76, 45.25, 42.08, 31.78, 30.98, 29.18, 27.41, 26.72, 24.63, 22.60, 14.07. ESI-HRMS calcd for C_22_H_34_N_3_O_3_^+^ ([M + H]^+^): 388.2595; found: 388.2598.

##### 7–(2-(4-Octylpiperazin-1-yl)-2-oxoethoxy)-3,4-dihydroquinolin-2(1H)-one (10e)

Yield: 76%. Mp: 104–105 °C. ^1^H-NMR (500 MHz, CDCl_3_, ppm) *δ*: 8.04 (*s*, 1H, CONH), 6.35–6.98 (*m*, 3H, Ar-H), 4.59 (*s*, 2H, -OCH_2_), 3.50–3.57 (*m*, 4H, Piperazine-H), 2.80–2.83 (*t*, *J* = 7.50 Hz, 2H, NHCOCH_2_), 2.52–2.55 (*t*, *J* = 7.50 Hz, 2H, NHCOCH_2_CH_2_), 2.34–2.37 (*m*, 4H, Piperazine-H), 1.21–2.27 (*m*, 14H, (-CH_2_-)_7_), 0.79–0.82 (*t*, 3H, *J* = 7.50 Hz, -CH_3_). ^13^C NMR (125 MHz, CDCl_3_) *δ*: 166.16, 157.49, 138.41, 128.79, 116.84, 108.69, 102.42, 67.72, 58.57, 53.39, 52.77, 45.29, 42.11, 31.82, 30.98, 29.49, 29.23, 27.47, 26.76, 24.62, 22.65, 14.09. ESI-HRMS calcd for C_23_H_36_N_3_O_3_^+^ ([M + H]^+^): 402.2751; found: 402.2756.

##### 7–(2-(4-Nonylpiperazin-1-yl)-2-oxoethoxy)-3,4-dihydroquinolin-2(1H)-one (10f)

Yield: 60%. Mp: 106–107 °C. ^1^H-NMR (500 MHz, CDCl_3_, ppm) *δ*: 8.17 (*s*, 1H, CONH), 6.35–6.98 (*m*, 3H, Ar-H), 4.59 (*s*, 2H, -OCH_2_), 3.50–3.58 (*m*, 4H, Piperazine-H), 2.80–2.83 (*t*, *J* = 7.50 Hz, 2H, NHCOCH_2_), 2.52–2.55 (*t*, *J* = 7.50 Hz, 2H, NHCOCH_2_CH_2_), 2.35–2.38 (*m*, 4H, Piperazine-H), 1.19–2.28 (*m*, 16H, (-CH_2_-)_8_), 0.79–0.82 (*t*, 3H, *J* = 7.50 Hz, -CH_3_). ^13^C NMR (125 MHz, CDCl_3_) *δ*: 170.53, 165.16, 156.47, 137.36, 127.81, 115.85, 107.68, 101.40, 66.72, 57.54, 52.36, 51.74, 44.24, 41.06, 30.85, 29.97, 28.51, 28.25, 26.44, 25.70, 23.61, 21.65, 13.08. ESI-HRMS calcd for C_24_H_38_N_3_O_3_^+^ ([M + H]^+^): 416.2908; found: 416.2913.

##### N-(4–(2-(4-benzylpiperazin-1-yl)-2-oxoethoxy)phenyl)acetamide (14a)

Yield: 42%. Mp: 124–125 °C. ^1^H-NMR (500 MHz, CDCl_3_, ppm) *δ*: 7.79 (*s*, 1H, CONH), 6.65–7.33 (*m*, 9H, Ar-H), 4.66 (*s*, 2H, -OCH_2_), 3.65 (*s*, 2H, -NCH_2_), 3.54–3.57 (*m*, 4H, Piperazine-H), 2.45–2.47 (*m*, 4H, Piperazine-H), 2.13 (*s*, 3H, -CH_3_). ^13^C NMR (125 MHz, CDCl_3_) *δ*: 168.61, 166.36, 158.26, 139.44, 129.81, 129.20, 128.40, 127.45, 112.96, 110.54, 106.12, 67.35, 62.68, 52.99, 52.50, 45.15, 42.00, 24.60. ESI-HRMS calcd for C_21_H_26_N_3_O_3_^+^ ([M + H]^+^): 368.1969; found: 368.1973.

##### N-(4–(2-(4–(4-fluorobenzyl)piperazin-1-yl)-2-oxoethoxy)phenyl)acetamide (14b)

Yield: 57%. Mp: 174–175 °C. ^1^H-NMR (500 MHz, CDCl_3_, ppm) *δ*: 7.63 (*s*, 1H, CONH), 6.84–7.40 (*m*, 8H, Ar-H), 4.64 (*s*, 2H, -OCH_2_), 3.55–3.63 (*m*, 4H, Piperazine-H), 3.46 (*s*, 2H, -NCH_2_), 2.39–2.42 (*m*, 4H, Piperazine-H), 2.13 (*s*, 3H, -CH_3_). ^13^C NMR (125 MHz, CDCl_3_) *δ*: 168.43, 166.45, 163.09, 161.14, 154.48, 133.25, 132.11, 130.58, 130.52, 128.77, 128.71, 121.84, 115.42, 115.26, 115.09, 114.93, 67.86, 61.95, 52.99, 52.54, 45.31, 42.13, 24.31. ESI-HRMS calcd for C_21_H_25_FN_3_O_3_^+^ ([M + H]^+^): 386.1874; found: 386.1882.

##### N-(4–(2-(4–(4-chlorobenzyl)piperazin-1-yl)-2-oxoethoxy)phenyl)acetamide (14c)

Yield: 76%. Mp: 179–180 °C. ^1^H-NMR (500 MHz, CDCl_3_, ppm) *δ*: 7.54 (*s*, 1H, CONH), 6.85–7.39 (*m*, 8H, Ar-H), 4.65 (*s*, 2H, -OCH_2_), 3.55–3.62 (*m*, 4H, Piperazine-H), 3.46 (*s*, 2H, -NCH_2_), 2.39–2.42 (*m*, 4H, Piperazine-H), 2.13 (*s*, 3H, -CH_3_). ^13^C NMR (125 MHz, CDCl_3_) *δ*: 168.38, 166.44, 154.50, 133.03, 132.07, 130.33, 128.52, 128.27, 121.85, 114.95, 67.89, 61.98, 53.03, 52.58, 45.31, 42.12, 24.33. ESI-HRMS calcd for C_21_H_25_ClN_3_O_3_^+^ ([M + H]^+^): 402.1579; found: 402.1586.

##### N-(4–(2-oxo-2–(4-(4-(trifluoromethyl)benzyl)piperazin-1-yl)ethoxy)phenyl)acetamide (14d)

Yield: 35%. Mp: 187–188 °C. ^1^H-NMR (500 MHz, CDCl_3_, ppm) *δ*: 7.65 (*s*, 1H, CONH), 6.85–7.59 (*m*, 8H, Ar-H), 4.66 (*s*, 2H, -OCH_2_), 3.58–3.63 (*m*, 4H, Piperazine-H), 3.56 (*s*, 2H, -NCH_2_), 2.39–2.43 (*m*, 4H, Piperazine-H), 2.13 (*s*, 3H, -CH_3_). ^13^C NMR (125 MHz, CDCl_3_) *δ*: 168.44, 166.48, 154.46, 141.91, 132.14, 129.15, 125.34, 125.31, 123.10, 121.87, 114.92, 67.88, 62.16, 53.12, 52.66, 45.29, 42.10, 24.30. ESI-HRMS calcd for C_22_H_25_FN_3_O_3_^+^ ([M + H]^+^): 436.1843; found: 436.1851.

##### N-(4–(2-(4–(4-methylbenzyl)piperazin-1-yl)-2-oxoethoxy)phenyl)acetamide (14e)

Yield: 53%. Mp: 180–181 °C. ^1^H-NMR (500 MHz, CDCl_3_, ppm) *δ*: 7.53 (*s*, 1H, CONH), 6.85–7.39 (*m*, 8H, Ar-H), 4.64 (*s*, 2H, -OCH_2_), 3.54–3.62 (*m*, 4H, Piperazine-H), 3.46 (*s*, 2H, -NCH_2_), 2.35–2.43 (*m*, 4H, Piperazine-H), 2.34 (*s*, 3H, -CH_3_), 2.13 (*s*, 3H, -COCH_3_). ^13^C NMR (125 MHz, CDCl_3_) *δ*: 168.34, 166.40, 154.54, 136.98, 132.05, 129.24, 129.10, 129.05, 127.12, 121.84, 114.97, 67.88, 65.24, 62.54, 53.07, 52.58, 45.33, 42.15, 24.34, 21.12. ESI-HRMS calcd for C_22_H_28_N_3_O_3_^+^ ([M + H]^+^): 382.2125; found:382.2137.

##### N-(4–(2-(4-butylpiperazin-1-yl)-2-oxoethoxy)phenyl)acetamide (15a)

Yield: 66%. Mp: 126–127 °C. ^1^H-NMR (500 MHz, CDCl_3_, ppm) *δ*: 7.82 (*s*, 1H, CONH), 6.77–7.32 (dd, 4H, Ar-H), 4.58 (s, 2H, -OCH_2_), 3.49–3.56 (*m*, 4H, Piperazine-H), 2.33–2.37 (*m*, 4H, Piperazine-H), 2.25–2.28 (*t*, *J* = 7.50 Hz, 2H, -N-CH_2_), 2.05 (*s*, 2H, COCH_3_), 1.23–1.41 (*m*, 4H, (-CH_2_-)_2_), 0.83–0.86 (*t*, 3H, *J* = 7.50 Hz, -CH_3_). ^13^C NMR (125 MHz, CDCl_3_) *δ*: 167.51, 165.38, 153.42, 131.21, 120.82, 113.88, 66.74, 57.19, 52.32, 51.74, 44.26, 41.09, 27.86, 23.24, 19.60, 12.98. ESI-HRMS calcd for C_18_H_28_N_3_O_3_^+^ ([M + H]^+^): 334.2125; found: 334.2136.

##### N-(4–(2-oxo-2–(4-pentylpiperazin-1-yl)ethoxy)phenyl)acetamide (15b)

Yield: 68%. Mp:116–117 °C. ^1^H-NMR (500 MHz, CDCl_3_, ppm) *δ*: 7.56 (*s*, 1H, CONH), 6.79–7.33 (dd, 4H, Ar-H), 4.59 (*s*, 2H, -OCH_2_), 3.50–3.57 (*m*, 4H, Piperazine-H), 2.34–2.37 (*m*, 4H, Piperazine-H), 2.24–2.37 (*t*, *J* = 7.50 Hz, 2H, -N-CH_2_), 2.06 (*s*, 2H, COCH_3_), 1.19–1.44 (*m*, 6H, (-CH_2_-)_3_), 0.81–0.84 (*t*, 3H, *J* = 7.50 Hz, -CH_3_). ^13^C NMR (125 MHz, CDCl_3_) *δ*: 167.37, 165.34, 153.49, 131.07, 120.82, 113.92, 66.81, 57.51, 52.33, 51.74, 44.25, 41.07, 28.61, 25.41, 23.29, 21.55, 13.01. ESI-HRMS calcd for C_19_H_30_N_3_O_3_^+^ ([M + H]^+^): 348.2282; found: 348.2286.

##### N-(4–(2-(4-hexylpiperazin-1-yl)-2-oxoethoxy)phenyl)acetamide (15c)

Yield: 49%. Mp: 131–132 °C. ^1^H-NMR (500 MHz, DMSO*_d6_*, ppm) *δ*: 9.78 (*s*, 1H, CONH), 6.83–7.46 (dd, 4H, Ar-H), 4.73 (*s*, 2H, -OCH_2_), 3.43 (*m*, 4H, Piperazine-H), 2.29–2.36 (*m*, 4H, Piperazine-H), 2.24–2.27 (*t*, *J* = 7.50 Hz, 2H, -N-CH_2_), 2.00 (*s*, 2H, COCH_3_), 1.26–1.41 (*m*, 8H, (-CH_2_-)_4_), 0.85–0.87 (*t*, 3H, *J* = 7.50 Hz, -CH_3_). ^13^C NMR (125 MHz, DMSO*_d6_*) *δ*: 168.19, 166.26, 154.21, 133.34, 120.86, 115.04, 66.71, 58.24, 53.41, 52.91, 44.74, 41.77, 31.69, 27.06, 26.63, 24.26, 22.57, 14.42. ESI-HRMS calcd for C_20_H_32_N_3_O_3_^+^ ([M + H]^+^): 362.2438; found: 362.2445.

##### N-(4–(2-(4-heptylpiperazin-1-yl)-2-oxoethoxy)phenyl)acetamide (15d)

Yield: 50%. Mp: 120–121 °C. ^1^H-NMR (500 MHz, CDCl_3_, ppm) *δ*: 7.72 (*s*, 1H, CONH), 6.85–7.40 (dd, 4H, Ar-H), 4.66 (*s*, 2H, -OCH_2_), 3.57–3.64 (*m*, 4H, Piperazine-H), 2.41–2.44 (*m*, 4H, Piperazine-H), 2.31–2.34 (*t*, *J* = 7.50 Hz, 2H, -N-CH_2_), 2.13 (*s*, 2H, COCH_3_), 1.27–1.49 (*m*, 10H, (-CH_2_-)_5_), 0.87–0.89 (*t*, 3H, *J* = 7.50 Hz, -CH_3_). ^13^C NMR (125 MHz, CDCl_3_) *δ*: 168.43, 166.38, 154.49, 132.15, 121.84, 114.93, 67.81, 58.55, 53.35, 52.76, 45.27, 42.10, 31.78, 29.19, 27.41, 26.75, 24.30, 22.61, 14.09. ESI-HRMS calcd for C_21_H_34_N_3_O_3_^+^ ([M + H]^+^): 376.2595; found: 376.2599.

##### N-(4–(2-(4-octylpiperazin-1-yl)-2-oxoethoxy)phenyl)acetamide (15e)

Yield: 44%. Mp: 118–119 °C. ^1^H-NMR (500 MHz, CDCl_3_, ppm) *δ*: 7.72 (*s*, 1H, CONH), 6.85–7.40 (dd, 4H, Ar-H), 4.66 (*s*, 2H, -OCH_2_), 3.60–3.66 (*m*, 4H, Piperazine-H), 2.44–2.46 (*m*, 4H, Piperazine-H), 2.34–2.37 (*t*, *J* = 7.50 Hz, 2H, -N-CH_2_), 2.13 (*s*, 2H, COCH_3_), 1.29–1.31 (*m*, 12H, (-CH_2_-)_6_), 0.87–0.89 (*t*, 3H, *J* = 7.50 Hz, -CH_3_). ^13^C NMR (125 MHz, CDCl_3_) *δ*: 168.48, 166.39, 154.48, 132.15, 121.87, 114.93, 67.80, 58.53, 53.29, 52.71, 45.11, 41.93, 31.82, 29.47, 29.23, 27.43, 26.60, 24.29, 22.66, 14.11. ESI-HRMS calcd for C_22_H_36_N_3_O_3_^+^ ([M + H]^+^): 390.2751; found: 390.2762.

##### N-(4–(2-(4-nonylpiperazin-1-yl)-2-oxoethoxy)phenyl)acetamide (15f)

Yield: 53%. Mp: 122–123 °C. ^1^H-NMR (500 MHz, CDCl_3_, ppm) *δ*: 7.68 (*s*, 1H, CONH), 6.85–7.40 (dd, 4H, Ar-H), 4.66 (*s*, 2H, -OCH_2_), 3.60–3.66 (*m*, 4H, Piperazine-H), 2.44–2.46 (*m*, 4H, Piperazine-H), 2.34–2.37 (*t*, *J* = 7.50 Hz, 2H, -N-CH_2_), 2.13 (*s*, 2H, COCH_3_), 1.26–1.31 (*m*, 14H, (-CH_2_-)_7_), 0.86–0.89 (*t*, 3H, *J* = 7.50 Hz, -CH_3_). ^13^C NMR (125 MHz, CDCl_3_) *δ*: 168.44, 166.38, 154.48, 132.13, 121.87, 114.92, 67.81, 58.52, 53.29, 52.71, 45.13, 41.94, 31.87, 29.52, 29.51, 29.26, 27.42, 26.60, 24.31, 22.67, 14.12. ESI-HRMS calcd for C_23_H_38_N_3_O_3_^+^ ([M + H]^+^): 404.2908; found: 404.2912.

##### N-(3–(2-(4-benzylpiperazin-1-yl)-2-oxoethoxy)phenyl)acetamide (17a)

Yield: 60%. Mp: 125–126 °C. ^1^H-NMR (500 MHz, CDCl_3_, ppm) *δ*: 7.74 (*s*, 1H, CONH), 6.57–7.19 (*m*, 9H, Ar-H), 4.58 (*s*, 2H, -OCH_2_), 3.46–3.55 (*m*, 4H, Piperazine-H), 3.40 (*s*, 2H, -NCH_2_), 2.32–2.37 (*m*, 4H, Piperazine-H), 2.05 (*s*, 3H, -CH_3_). ^13^C NMR (125 MHz, CDCl_3_) *δ*: 168.48, 166.34, 158.34, 139.39, 136.96, 134.41, 129.81, 129.09, 129.03, 112.97, 110.59, 106.20, 77.29, 77.04, 76.79, 67.46, 62.51, 53.06, 52.53, 45.32, 42.16, 24.59, 21.10. ESI-HRMS calcd for C_21_H_26_N_3_O_3_^+^ ([M + H]^+^): 368.1969; found: 368.1980.

##### N-(3–(2-(4–(4-fluorobenzyl)piperazin-1-yl)-2-oxoethoxy)phenyl)acetamide (17b)

Yield: 63%. Mp: 152–153 °C. ^1^H-NMR (500 MHz, CDCl_3_, ppm) *δ*: 7.61 (*s*, 1H, CONH), 6.58–7.22 (*m*, 8H, Ar-H), 4.59 (*s*, 2H, -OCH_2_), 3.50–3.57 (*m*, 4H, Piperazine-H), 3.40 (*s*, 2H, -NCH_2_), 2.36–2.39 (*m*, 4H, Piperazine-H), 2.06 (*s*, 3H, -CH_3_). ^13^C NMR (125 MHz, CDCl_3_) *δ*: 167.57, 165.40, 162.16, 160.21, 157.26, 138.39, 129.71, 128.83, 127.76, 127.70, 114.41, 114.30, 114.25, 114.13, 111.97, 109.61, 105.16, 66.43, 63.56, 60.82, 52.41, 51.88, 51.41, 49.75, 44.15, 40.99, 23.59. ESI-HRMS calcd for C_21_H_25_FN_3_O_3_^+^ ([M + H]^+^): 386.1874; found: 386.1885.

##### N-(3–(2-(4–(4-chlorobenzyl)piperazin-1-yl)-2-oxoethoxy)phenyl)acetamide (17c)

Yield: 67%. Mp: 138–139 °C. ^1^H-NMR (500 MHz, CDCl_3_, ppm) *δ*: 7.72 (*s*, 1H, CONH), 6.57–7.22 (*m*, 8H, Ar-H), 4.58 (*s*, 2H, -OCH_2_), 3.46–3.54 (*m*, 4H, Piperazine-H), 3.40 (*s*, 2H, -NCH_2_), 2.32–2.35 (*m*, 4H, Piperazine-H), 2.05 (*s*, 3H, -CH_3_). ^13^C NMR (125 MHz, CDCl_3_) *δ*: 168.45, 166.39, 158.32, 139.36, 136.14, 133.04, 130.32, 129.84, 128.51, 112.94, 110.60, 106.17, 67.54, 61.96, 53.03, 52.55, 45.31, 42.13, 24.62. ESI-HRMS calcd for C_21_H_25_ClN_3_O_3_^+^ ([M + H]^+^): 402.1579; found: 402.1588.

##### N-(3–(2-oxo-2–(4-(4-(trifluoromethyl)benzyl)piperazin-1-yl)ethoxy)phenyl)acetamide (17d)

Yield: 49%. Mp: 125–126 °C. ^1^H-NMR (500 MHz, CDCl_3_, ppm) *δ*: 7.60 (*s*, 1H, CONH), 6.59–7.51 (*m*, 8H, Ar-H), 4.60 (*s*, 2H, -OCH_2_), 3.49–3.56 (*m*, 4H, Piperazine-H), 3.48 (*s*, 2H, -NCH_2_), 2.34–2.38 (*m*, 4H, Piperazine-H), 2.07 (*s*, 3H, -CH_3_). ^13^C NMR (125 MHz, CDCl_3_) *δ*: 168.52, 166.41, 158.30, 139.40, 129.84, 129.16, 125.33, 125.30, 123.10, 112.95, 110.58, 106.17, 67.53, 62.14, 53.11, 52.63, 45.29, 42.11, 24.60. ESI-HRMS calcd for C_22_H_25_FN_3_O_3_^+^ ([M + H]^+^): 436.1843; found: 436.1845.

##### N-(3–(2-(4–(4-methylbenzyl)piperazin-1-yl)-2-oxoethoxy)phenyl)acetamide (17e)

Yield: 51%. Mp: 126–127 °C. ^1^H-NMR (500 MHz, CDCl_3_, ppm) *δ*: 7.61 (*s*, 1H, CONH), 6.58–7.29 (*m*, 8H, Ar-H), 5.23 (*s*, 3H, -CH_3_), 4.59 (*s*, 2H, -OCH_2_), 3.47–3.56 (*m*, 4H, Piperazine-H), 3.43 (s, 2H, -NCH_2_), 2.34–2.38 (*m*, 4H, Piperazine-H), 2.06 (*s*, 3H, -CH_3_). ^13^C NMR (125 MHz, CDCl_3_) *δ*: 168.43, 166.32, 158.35, 136.94, 134.46, 129.81, 129.08, 129.03, 112.97, 110.56, 106.20, 67.46, 62.52, 53.07, 52.54, 45.34, 42.18, 24.60, 21.10. ESI-HRMS calcd for C_22_H_28_N_3_O_3_^+^ ([M + H]^+^): 382.2125; found: 382.2137.

##### N-(3–(2-(4-butylpiperazin-1-yl)-2-oxoethoxy)phenyl)acetamide (18a)

Yield: 48%. Mp: 132–133 °C. ^1^H-NMR (500 MHz, CDCl_3_, ppm) *δ*: 7.65 (*s*, 1H, CONH), 6.59–7.18 (*m*, 4H, Ar-H), 4.60 (*s*, 2H, -OCH_2_), 3.49–3.58 (*m*, 4H, Piperazine-H), 2.34–2.38 (*m*, 4H, Piperazine-H), 2.26–2.29 (*t*, *J* = 7.50 Hz, 2H, -N-CH_2_), 2.06 (*s*, 2H, COCH_3_), 1.23–1.42 (*m*, 4H, (-CH_2_-)_2_), 0.83–0.86 (*t*, 3H, *J* = 7.50 Hz, -CH_3_). ^13^C NMR (125 MHz, CDCl_3_) *δ*: 168.65, 166.31, 158.29, 139.47, 129.78, 112.99, 110.50, 106.21, 67.31, 58.20, 53.29, 52.73, 45.24, 42.09, 28.85, 24.56, 20.62, 13.99. ESI-HRMS calcd for C_18_H_28_N_3_O_3_^+^ ([M + H]^+^): 334.2125; found: 334.2132.

##### N-(3–(2-oxo-2–(4-pentylpiperazin-1-yl)ethoxy)phenyl)acetamide (18b)

Yield: 55%. Mp: 90–91 °C. ^1^H-NMR (500 MHz, CDCl_3_, ppm) *δ*: 7.86 (*s*, 1H, CONH), 6.58–7.17 (*m*, 4H, Ar-H), 4.60 (*s*, 2H, -OCH_2_), 3.50–3.58 (*m*, 4H, Piperazine-H), 2.36–2.41 (*m*, 4H, Piperazine-H), 2.26–2.29 (*t*, *J* = 7.50 Hz, 2H, -N-CH_2_), 2.06 (*s*, 2H, COCH_3_), 1.20–1.42 (*m*, 6H, (-CH_2_-)_3_), 0.81–0.84 (*t*, 3H, *J* = 7.50 Hz, -CH_3_). ^13^C NMR (125 MHz, CDCl_3_) *δ*: 167.75, 165.40, 157.24, 138.49, 128.80, 112.02, 109.58, 105.14, 66.32, 57.45, 52.21, 51.66, 44.11, 40.98, 28.59, 25.28, 23.53, 21.53, 13.00. ESI-HRMS calcd for C_19_H_30_N_3_O_3_^+^ ([M + H]^+^): 348.2282; found: 348.2289.

##### N-(3–(2-(4-hexylpiperazin-1-yl)-2-oxoethoxy)phenyl)acetamide (18c)

Yield: 73%. Mp: 63–64 °C. ^1^H-NMR (500 MHz, CDCl_3_, ppm) *δ*: 7.72 (*s*, 1H, CONH), 6.66–7.26 (*m*, 4H, Ar-H), 4.67 (*s*, 2H, -OCH_2_), 3.58–3.65 (*m*, 4H, Piperazine-H), 2.43–2.46 (*m*, 4H, Piperazine-H), 2.33–2.36 (*t*, *J* = 7.50 Hz, 2H, -N-CH_2_), 2.14 (*s*, 2H, COCH_3_), 1.29–1.48 (*m*, 8H, (-CH_2_-)_4_), 0.87–0.90 (*t*, 3H, *J* = 7.50 Hz, -CH_3_). ^13^C NMR (125 MHz, CDCl_3_) *δ*: 168.59, 166.33, 158.29, 139.41, 129.81, 112.99, 110.59, 106.19, 67.40, 58.50, 53.25, 52.69, 45.19, 42.03, 31.71, 27.10, 26.61, 24.59, 22.58, 14.02. ESI-HRMS calcd for C_20_H_32_N_3_O_3_^+^ ([M + H]^+^): 362.2438; found: 362.2445.

##### N-(3–(2-(4-heptylpiperazin-1-yl)-2-oxoethoxy)phenyl)acetamide (18d)

Yield: 46%. Mp: 65–66 °C. ^1^H-NMR (500 MHz, CDCl_3_, ppm) *δ*: 7.75 (*s*, 1H, CONH), 6.59–7.17 (*m*, 4H, Ar-H), 4.60 (*s*, 2H, -OCH_2_), 3.51–3.58 (*m*, 4H, Piperazine-H), 2.35–2.39 (*m*, 4H, Piperazine-H), 2.25–2.28 (*t*, *J* = 7.50 Hz, 2H, -N-CH_2_), 2.07 (*s*, 2H, COCH_3_), 1.21–1.43 (*m*, 10H, (-CH_2_-)_5_), 0.80–0.83 (*t*, 3H, *J* = 7.50 Hz, -CH_3_). ^13^C NMR (125 MHz, CDCl_3_) *δ*: 167.62, 165.34, 157.29, 138.45, 128.82, 111.97, 109.58, 105.13, 66.41, 57.52, 52.28, 51.71, 44.22, 41.06, 30.77, 28.17, 26.40, 25.69, 23.56, 21.59, 13.05. ESI-HRMS calcd for C_21_H_34_N_3_O_3_^+^ ([M + H]^+^): 376.2595; found: 376.2601.

##### N-(3–(2-(4-octylpiperazin-1-yl)-2-oxoethoxy)phenyl)acetamide (18e)

Yield: 55%. Mp: 93–94 °C. ^1^H-NMR (500 MHz, CDCl_3_, ppm) *δ*: 7.48 (*s*, 1H, CONH), 6.65–7.24 (*m*, 4H, Ar-H), 4.66 (*s*, 2H, -OCH_2_), 3.57–3.64 (*m*, 4H, Piperazine-H), 2.41–2.43 (*m*, 4H, Piperazine-H), 2.31–2.34 (*t*, *J* = 7.50 Hz, 2H, -N-CH_2_), 2.12 (*s*, 2H, COCH_3_), 1.28–1.50 (*m*, 12H, (-CH_2_-)_6_), 0.86–0.89 (*t*, 3H, *J* = 7.50 Hz, -CH_3_). ^13^C NMR (125 MHz, CDCl_3_) *δ*: 168.38, 166.30, 158.31, 139.35, 129.78, 112.99, 110.54, 106.21, 67.52, 67.36, 58.54, 53.32, 52.75, 45.29, 42.12, 31.82, 29.48, 29.23, 27.46, 26.75, 24.55, 22.64, 14.08. ESI-HRMS calcd for C_22_H_36_N_3_O_3_^+^ ([M + H]^+^): 390.2751; found: 390.2756

##### N-(3–(2-(4-nonylpiperazin-1-yl)-2-oxoethoxy)phenyl)acetamide (18f)

Yield: 53%. Mp: 84–85 °C. ^1^H-NMR (500 MHz, CDCl_3_, ppm) *δ*: 8.55 (*s*, 1H, CONH), 6.56–7.25 (*m*, 4H, Ar-H), 4.68 (*s*, 2H, -OCH_2_), 3.78 (*m*, 4H, Piperazine-H), 3.03–3.12 (*m*, 4H, Piperazine-H), 2.79 (*t*, *J* = 7.50 Hz, 2H, -N-CH_2_), 2.08 (*s*, 2H, COCH_3_), 1.18–1.62 (*m*, 14H, (-CH_2_-)_7_), 0.79–0.82 (*t*, 3H, *J* = 7.50 Hz, -CH_3_). ^13^C NMR (125 MHz, CDCl_3_) *δ*: 168.38, 165.81, 156.93, 138.79, 128.94, 112.45, 109.79, 105.16, 65.78, 56.70, 52.44, 50.48, 41.44, 38.51, 30.80, 28.39, 28.15, 28.13, 25.82, 23.71, 23.34, 21.63, 13.09. ESI-HRMS calcd for C_23_H_38_N_3_O_3_^+^ ([M + H]^+^): 404.2908; found: 404.2915.

### Pharmacology

#### Animals and treatment

FST, TST, OFT, CUMS and 5-HT level experiments were performed using Kunming mice that were 3–4 weeks old, specific pathogen-free (SPF), and weighed 20 ± 1 g (obtained from Ji’nan Pengyue Laboratory Animal Breeding Co., Ltd, Ji’nan, China). Mice were allowed to drink freely only during the first 12 h of the experiment, otherwise they were kept in normal conditions. In the paper, all experimental mice were executed by cervical dislocation after CO_2_ inhalation. Fluoxetine was chosen as the reference drug for the experiment. All compounds tested were dissolved in dimethyl sulfoxide/0.5% sodium carboxymethylcellulose and were administered by intraperitoneal injection or gavage. The protocol for this study was reviewed and approved by the Special Committee on Scientific Research Ethic of Liaocheng University (Approval number: 2022091201).

#### FST

FST was conducted in accordance with previously reported methods^22–24^. Kunming mice (20 ± 1 g) were used in the FST. On the test day, mice were assigned to different groups (control, compounds and fluoxetine group, *n* = 8 for each group). The synthesised compounds and fluoxetine were administered by intraperitoneal (dose: 40 mg/kg, volume: 0.05 ml/10g body weight), and the control group was injected with the same volume of dimethyl sulfoxide. 30 min after the injection, the mice were dropped one at a time into a Plexiglas cylinder (height 50 cm, diameter 25 cm, containing water to a height of 15 cm at 23–25 °C) and observed for 6 min. After the first 2 min of vigorous struggling, the mice were immobile. A mouse was judged immobile if it floated in the water in an upright position and made only slight movements to prevent sinking. The total duration of immobility was recorded during the last 4 min of the 6-min test. The ability to significantly reduce the immobility time of the mice compared to the control group can be considered as a potential antidepressant activity.

#### TST

TST was conducted in accordance with previously reported methods^22–24^. Kunming mice were assigned to different groups (control, compounds and fluoxetine group, *n* = 8 for each group). The synthesised compounds and fluoxetine were administered by intraperitoneal (dose: 40 mg/kg, volume: 0.05 ml/10g body weight), and the control group was injected with the same volume of dimethyl sulfoxide. 30 min after the injection, the mouse is suspended within its own three-walled rectangular compartment (55 × 15 × 11.5 cm). The mice is suspended in the middle of this compartment, hang the mice 2 cm away from the tail end of the tape, making it unable to escape or grasp the nearby surface and preventing the animal from contacting the floor or escaping. The total duration of immobility was recorded during the last 4 min of the 6 min test.

#### OFT

Compounds with potential antidepressant activity were obtained by screening with the FST model. The OFT assay was used to verify whether the potential compounds affected the spontaneous behaviour of mice in unfamiliar environments, such as locomotion (number of line crossings), rearing (number of times seen standing on hind legs) and grooming (number of modifications)^25^. The experiment was divided into control and compound group (*n* = 8 for each group). Compounds **6a** and **18a** were administered by intraperitoneal (dose: 40 mg/kg, volume: 0.05 ml/10g body weight), and the control group was injected with the same volume of dimethyl sulfoxide. 30 min after the injection, the mice were placed in a 40 × 50 × 50 cm black plastic box for 6 min and the spontaneous behaviour of the mice was recorded for the next 4 min. The experiments were performed in a dark room, and the apparatus was illuminated by a 60-W bulb giving a yellowish light, positioned 1 m above the centre of the apparatus.

#### 5-HT level determination

The 5-HT content was measured according to previously reported method^26^. Enzyme-linked immunosorbent assay (ELISA) was performed to determine the 5-HT levels in hippocampal tissues of each group of mice. Mice were executed by cervical dislocation after CO_2_ inhalation. Hippocampal tissue from mice brain was extracted, weighed, and the entire hippocampal tissue was homogenised in PBS (pH = 7.4), which was prepared as a 10% brain tissue homogenate. Centrifuged at 4 °C for 20 min at 3000 r/min (8.5 cm radius) and the supernatant was obtained. The ELISA kit (No: MM-0443M1, MM-0443M2; Jiangsu Meimian industrial Co., Ltd) instructions were strictly followed. Place all reagents in the kit at room temperature 30 min in advance.

Set standard wells, testing sample wells, add standard 50 μl to standard well. Set blank wells (blank comparison wells don’t add sample and HRP-Conjugate reagent, other each step operation is same), testing sample well. Add sample dilution 40 μl to testing sample well, then add testing sample 10 μl (sample final dilution is 5-fold), add sample to wells, do not touch the well wall as far as possible. Add HRP-Conjugate reagent 100 μl to each well, except blank well. After closing plate with closure plate membrane, incubate for 60 min at 37 °C. Twentyfold wash solution diluted 20-fold with distilled water and reserve. Uncover closure plate membrane, discard liquid, dry by swing, add washing buffer to every well, still for 30 s then drain, repeat 5 times, dry by pat. Add chromogen solution A50 ul and chromogen solution B50 ul to each well, evade the light preservation for 15 min at 37 °C. Add stop solution 50 μl to each well, stop the reaction (the blue colour change to yellow colour). Take blank well as zero, read absorbance at 450 nm after adding stop solution and within 15 min.

#### 5-HT_1A_ binding assay

The affinity of potential compounds **6a**, **18a** and serotonin for 5-HT_1A_R was determined using the method reported in the previous literature^27^. Cell membrane homogenates (20 µg protein) are incubated for 60 min at 22 °C with 0.3 nM [^3^H]8-OH-DPAT in the absence or presence of the test compound in a buffer containing 10 mM MgSO_4_, 50 mM Tris-HCl (pH = 7.4), 0.5 mM EDTA and 0.1% ascorbic acid in buffer. Non-specific binding is determined in the presence of 5 µM 8-OH-DPAT. Following incubation, the samples are filtered rapidly under vacuum through glass fibre filters pre-soaked with 0.3% PEI and rinsed several times with ice-cold 50 mM Tris-HCl using a 96-sample cell harvester. The filters are dried then counted for radioactivity in a scintillation counter using a scintillation cocktail. The results are expressed as a percent inhibition of the control radioligand specific binding.

#### CUMS model

The CUMS procedure was adjusted with reference to literature and described as follows^28^^,^^29^. Kunming mice (Male, weight 20 ± 1 g) were acclimatised and fed for 3 days in a room with a 12 h light/dark cycle, 45 ± 10% air humidity and free access to food and water. On the test day, 40 mice were assigned to different groups (control, model, fluoxetine and **6a** group, *n* = 10 for each group). At the end of the acclimatisation period, each group was given one unpredictable stimulus per day for 5 weeks, except for the control group, which was kept as control. The stimuli included cold water swimming (4 °C) for 5 min, warm water swimming (30 °C) for 5 min, food deprivation for 12 h, prohibition of water for 12 h, cage tilt at 30 °C for 12 h, moist bedding for 12 h, and reversal of day and night for 24 h. Administration began on day 15, compound **6a** and fluoxetine were administered by gavage (dose: 40 mg/kg; volume: 0.1 ml/10g body weight) once daily, and the control group also was administered with the same volume of 0.5% sodium carboxymethylcellulose (0.1 ml/10g body weight) once daily. At the end of the modelling period, the success of the modelling was verified by FST and changes in body mass of the mice.

#### HE staining

The procedure was adjusted with reference to the literature and is described as follows^30^^,^^31^. After CUMS modelling, the mice were executed by cervical dislocation after CO_2_ inhalation and the whole brain is removed and fixed in saline and stored in 4% paraformaldehyde solution. The whole procedure was carried out quickly and on ice. Mice brain tissue was embedded in paraffin and sectioned, dewaxed in xylene, washed in water, stained with haematoxylin staining solution for 5 min, washed in water, fractionated in fractionation solution and washed in water, counterblue solution and rinsed in water for 10 min. The slices were dehydrated in 85% and 95% alcohol and then stained with eosin for 5 min. after dehydration, the slices were sealed with neutral gum. The sections were then sealed with neutral gum after dehydration. The sections were observed by microscope and images were collected for analysis. The reagents were purchased from Sinopharm Chemical Reagent Co., Ltd and Wuhan Xavier Biotechnology Co., Ltd.

#### Western blot analysis

The procedure was adjusted with reference to the literature and is described as follows^30^^,^^31^. After CUMS modelling, the mice were executed by cervical dislocation after CO_2_ inhalation and the whole brain is removed and rinsed in saline and stored in 4% paraformaldehyde solution. The whole procedure was carried out rapidly and on ice. The concentration of brain tissue proteins was calibrated to 2 mg/ml using the BCA protein assay kit (P0012, Beyotime) and each group of protein samples was separated on a 10% SDS-PAGE gel. Subsequently, they were transferred to PVDF membranes and blocked with 5% skimmed milk powder for 1 h. The Primary antibodies against 5-HT_1A_R (Anti-HTR_1A_ Antibody, Rabbit IgG, 1:1000, BA1391, Boster), PKA (Anti-PKA alpha + beta Rabbit pAb, 1:1000, GB11598, Servicebio), BDNF (Anti-BDNF Rabbit pAb, 1: 1000, GB11559, Servicebio), GAPDH (Recombinant Anti-GAPDH antibody, Rabbit IgG, 1:1500, GB15004, Servicebio) were incubated with the membranes at 4 °C overnight. After washes with TBST 3 times, secondary antibodies (HRP Conjugated AffiniPure Goat Anti-rabbit IgG, 1:5000, BA1054, Boster) were diluted and incubated with the membranes at room temperature for 1 h. Images were observed by Tanon 4600 and protein expression was calculated using Image J.

#### Immunohistochemical analysis

The procedure was adjusted with reference to literature and is described as follows^30^^,^^31^. Mice brain tissue was embedded in paraffin and sectioned, dewaxed in dewaxing solution, placed in a repair cassette in citric acid antigen repair solution (pH = 6.0) for antigen repair, 3% hydrogen peroxide solution blocked for 25 min at room temperature and rinsed with PBS (pH = 7.4). Tissue was blocked with 3% BSA for 30 min at room temperature, primary antibody (Anti-5-HT_1A_ Receptor Rabbit pAb, GB114285-100, Servicebio) was incubated overnight at 4 °C, secondary antibody (HRP conjugated Goat Anti-Rabbit IgG, GB23303, Servicebio) was incubated for 1 h at room temperature, DAB chromogenic solution was used for colour development, haematoxylin was used for re-staining for 3 min, the film was dehydrated and sealed with blocking gel. Images were taken and observed by microscopy and protein expression was calculated using Image J. Reagents were purchased from Sinopharm Chemical Reagent Co., Ltd and Wuhan Xavier Biotechnology Co., Ltd.

#### In silico studies

In this study, the main focus was on molecular docking of potential target compounds to target proteins using DS 2021 software to simulate possible compound-protein interactions. The molecular formulae of all the target compounds were constructed using Sketch Molecules in the Small Molecules module of the DS 2021 software, and the physicochemical parameters (MW, nHBD, nHBA, RotB, TPSA, and CLogP) and pharmacokinetic parameters (BBB and ABS) were predicted for all the target compounds.

## Supplementary Material

Supplemental Material
