# Supplementary material for: Design, synthesis, and biological evaluation of piperazine derivatives involved in the 5-HT1AR/BDNF/PKA pathway
Source: J Enzyme Inhib Med Chem. 2023 Dec 11;39(1):2286183. doi: 10.1080/14756366.2023.2286183 (PMC11721615; doi:10.1080/14756366.2023.2286183)

Design, synthesis, and biological evaluation of piperazine derivatives involved in the 5-HT$_{1A}$R/BDNF/PKA pathway

In silico studies

In this study, the main focus was on molecular docking of potential target compounds to target proteins using DS2021 software to simulate possible compound-protein interactions. The crystal structure of serotonin with 5-HT$_{1A}$R (PDB: 7E2Y) was downloaded from the PDB database (https://www.rcsb.org/). The DS 2021 software was used to pre-process 5-HT$_{1A}$R and to find the site of action of serotonin, which was set as a spherical region with x = 103.03, y = 114.78, z = 108.36, Radius = 8 for molecular docking of the target compound. Molecular docking was performed using the CDOCKER method in DS 2021 software and the docking results were processed and analysed under this module.

The molecular formula of all the target compounds were constructed using Sketch Molecules in the Small Molecules module of the DS 2021 software, and the physicochemical parameters (MW, nHBD, nHBA, RotB, TPSA, CLogP), pharmacokinetic parameters (BBB and ABS) were predicted for all the target compounds (**Table** 2 and **Figure** 14).

Table 2. Physiochemical properties of target compounds.

Compds	MW[a]	ClogP	nHBD	nHBA	nRot	TPSA	Compds	MW[a]	ClogP	nHBD	nHBA	nRot	TPSA
**6a**	379	2.96	1	4	5	63	**14a**	367	2.83	1	4	6	63
**6b**	397	3.10	1	4	5	63	**14b**	385	2.97	1	4	6	63
**6c**	413	3.67	1	4	5	63	**14c**	401	3.54	1	4	6	63
**6d**	447	3.84	1	4	6	63	**14d**	435	3.71	1	4	7	63
**6e**	393	3.46	1	4	5	63	**14e**	333	3.32	1	4	7	63
**7a**	345	2.83	1	4	6	63	**15a**	381	2.69	1	4	6	63
**7b**	359	3.35	1	4	7	63	**15b**	347	3.22	1	4	8	63
**7c**	373	3.88	1	4	8	63	**15c**	361	3.75	1	4	9	63
**7d**	387	4.41	1	4	9	63	**15d**	375	4.28	1	4	10	63
**7e**	401	4.94	1	4	10	63	**15e**	389	4.81	1	4	11	63
**7f**	415	5.47	1	4	11	63	**15f**	403	5.34	1	4	12	63
**9a**	379	2.96	1	4	5	63	**17a**	367	2.83	1	4	6	63
**9b**	397	3.10	1	4	5	63	**17b**	385	2.97	1	4	6	63
**9c**	413	3.67	1	4	5	63	**17c**	401	3.54	1	4	6	63
**9d**	447.	3.84	1	4	6	63	**17d**	435	3.71	1	4	7	63
**9e**	345	3.46	1	4	6	63	**17e**	333	3.32	1	4	7	63
**10a**	393	2.83	1	4	5	63	**18a**	381	2.69	1	4	6	63
**10b**	359	3.35	1	4	7	63	**18b**	347	3.22	1	4	8	63

**10c**	373	3.88	1	4	8	63	**18c**	361	3.75	1	4	9	63
**10d**	387	4.41	1	4	9	63	**18d**	375	4.28	1	4	10	63
**10e**	401	4.94	1	4	10	63	**18e**	389	4.81	1	4	11	63
**10f**	415	5.47	1	4	11	63	**18f**	403	5.34	1	4	12	63

[a] MW, molecular weight; CLogP, calculated lipophilicity; nHBD, number of hydrogen bond donors; nHBA, number of hydrogen bond acceptors; nRotB, number of rotatable bonds; TPSA, topological polar surface area.

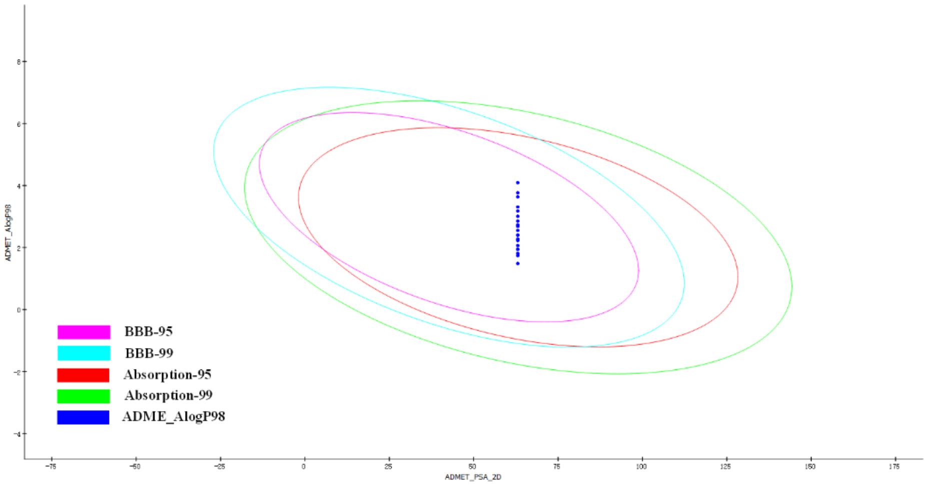

Figure 14. ADME/T value prediction for target compounds. The blue dots in the four circular circles indicate that the compound has good pharmacokinetic properties.

To conform the stability of the protein-ligand complex, the classical molecular dynamics (MD) simulation was performed using the Standard Dynamics Cascade protocol of the Discovery Studio software (DS v2021; Accelrys, Inc., San Diego, CA, USA)[1]. The optimal configuration from the docking calculations were used as the initial configuration of the MD simulation. The charmm27 force field[2,3] was adopted to describe the intermolecular and intramolecular interactions. To setup the simulation, the complex was first solvated in a rectangular box with dimensions of 61.7 × 63.4 × 99.9 Å^3 (Figure 1). So far, the minimum distance of the complex from the periodic boundary was specified as 0.9 Å. Water molecules were described using the TIP3P model[4]. The counterions were added to the solution to keep the electrical neutrality of the system. The 50,000-step energy minimization process is carried out using the steepest method of energy descent. Then, a 10 ns simulation under the NPT ensemble was carried out at 298 K and 1 atm to achieve the equilibration of the system. Subsequently,

a 10 ns simulation under the NVT ensemble was performed as the production period. During the simulation, periodic conditions were applied in the three directions. LINCS algorithm[5] was applied to constrain the bond lengths of other components. The temperature was maintained using the V-rescale thermostat algorithm6. The pressure control was changed to the Parrinello-Rahman method in the production run. The cut-off distance for the Lennard-Jones and electrostatic interactions was 1.2 nm. Particle mesh Ewald method[7] was used to calculate the long-range electrostatic interactions. After the simulation, the RMSD of the complex was analyzed as shown in Figure 2. It can be seen that the fluctuation range is kept within 0.5 Å, which confirms the stability of the complex. On the basis of the configuration from the MD simulation, the intermolecular interactions was further analyzed.

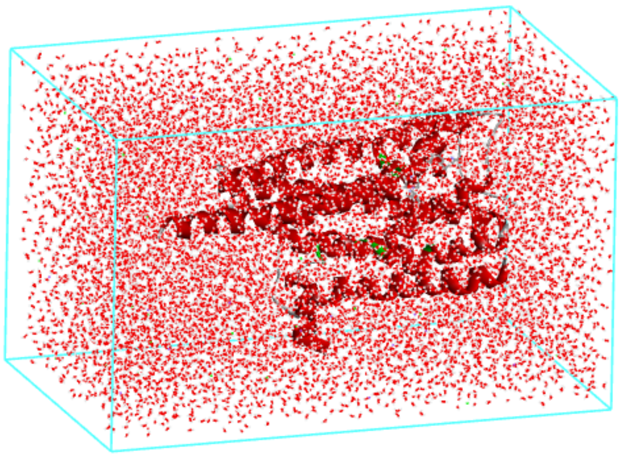

Figure 15. Configuration of the solvated complex for the MD simulation. The protein is shown in Cartoon model and water molecules are shown in red line for clarity.

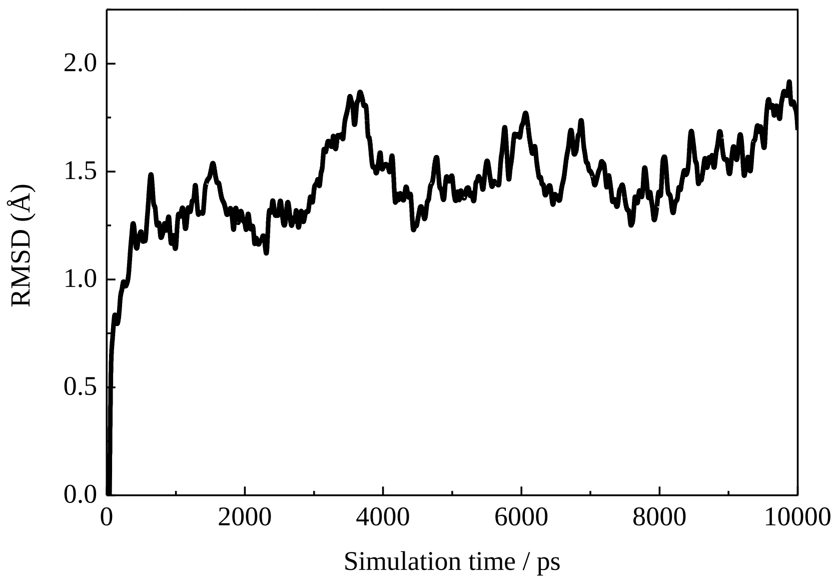

Figure 16. RMSD of the complex during the simulation.

References

[1] Rao SN, Head MS, Kulkarni A, LaLonde JM. Validation studies of the site-directed docking program LibDock. J Chem Inf Model. 2007;47:2159-2171.

[2] Foloppe N, MacKerell Jr AD. All-atom empirical force field for nucleic acids: I. Parameter optimization based on small molecule and condensed phase macromolecular target data. J Comput Chem. 1999;21:86-104.

[3] Fraczkiewicz R, Braun W. Exact and efficient analytical calculation of the accessible surface areas and their gradients for macromolecules. J Comput Chem. 1997;19(3):319-333.

[4] Jorgensen WL, Chandrasekhar J, Madura JD, Impey RW, Klein ML. Comparison of simple potential functions for simulating liquid water. J Chem Phys. 1983;79:926-935.

[5] Hess B, Bekker H, Berendsen HJC, Fraaije JGEM. LINCS: A linear constraint solver for molecular simulations. J Comput Chem. 1997;18:1463-1472.

[6] Bussi G, Donadio D, Parrinello M. Canonical sampling through velocity rescaling. J Phys Chem. 2007;126:014101.

[7] Essmann U, Perera L, Berkowitz ML, Darden T, Lee H, Pedersen LG. A smooth particle mesh Ewald method. J Phys Chem. 1995;103:8577-8593.

^{1}H-NMR and ^{13}C-NMR spectra of the target compound:

6-(2-(4-Benzylpiperazin-1-yl)-2-oxoethoxy)-3,4-dihydroquinolin-2(1H)-one (6a)

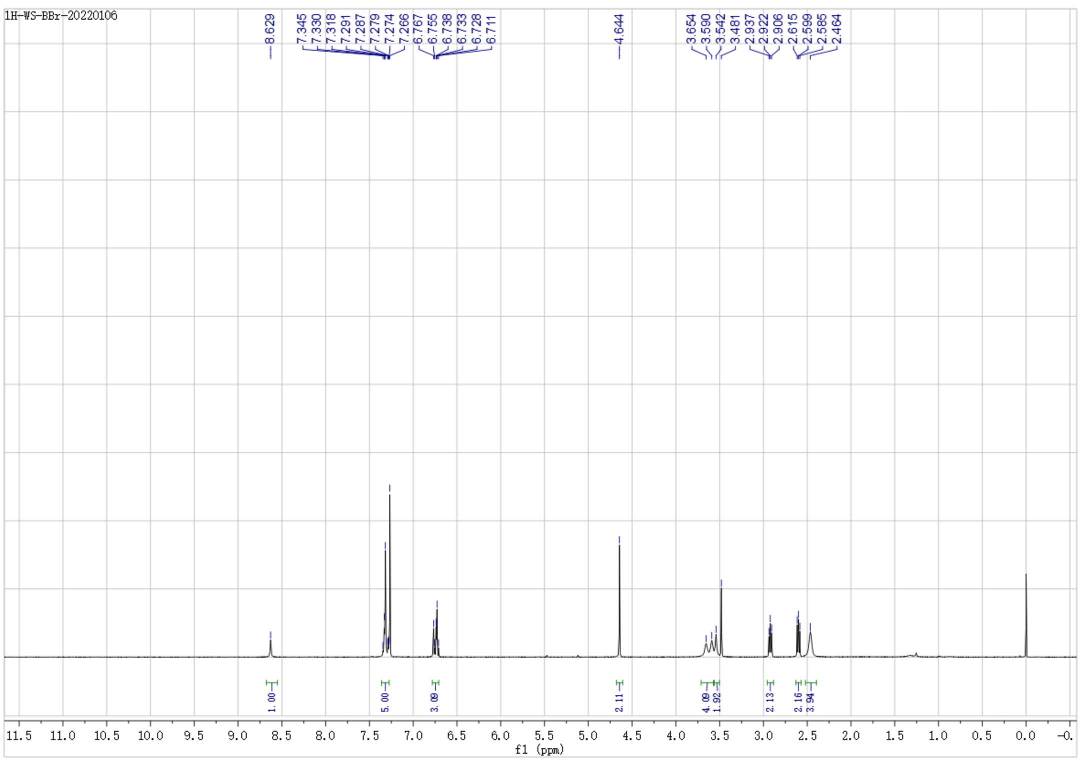

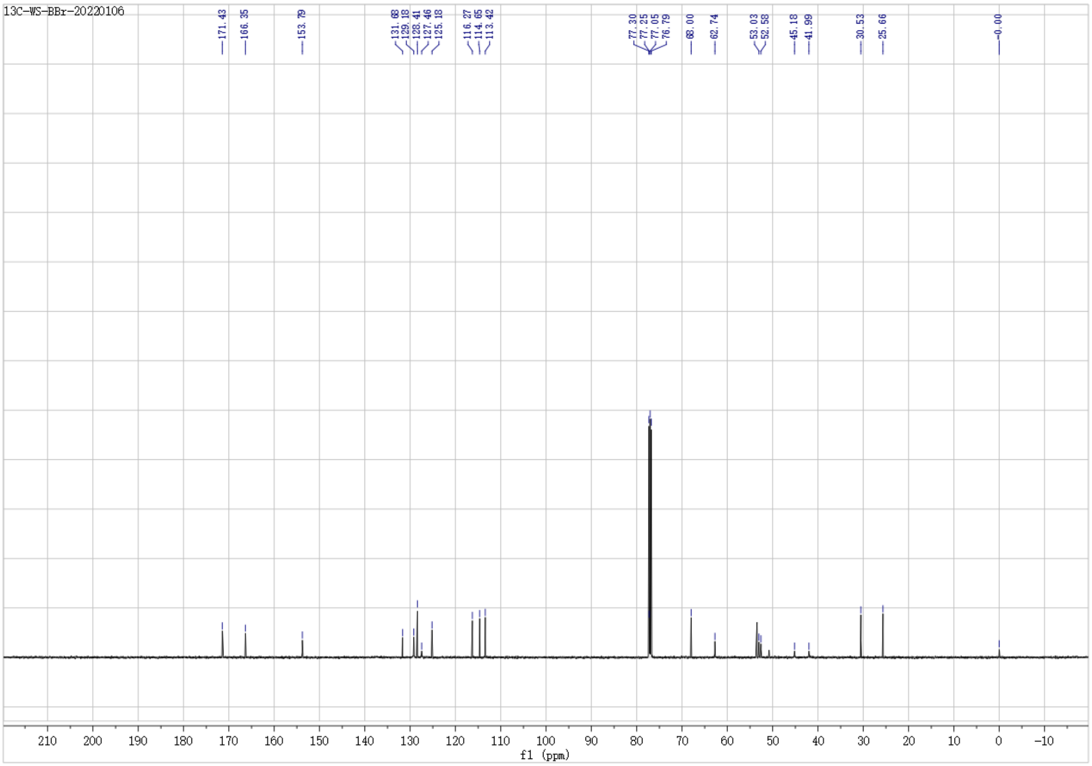

6-(2-(4-(4-Fluorobenzyl)piperazin-1-yl)-2-oxoethoxy)-3,4-dihydroquinolin-2(1H)-one (6b)

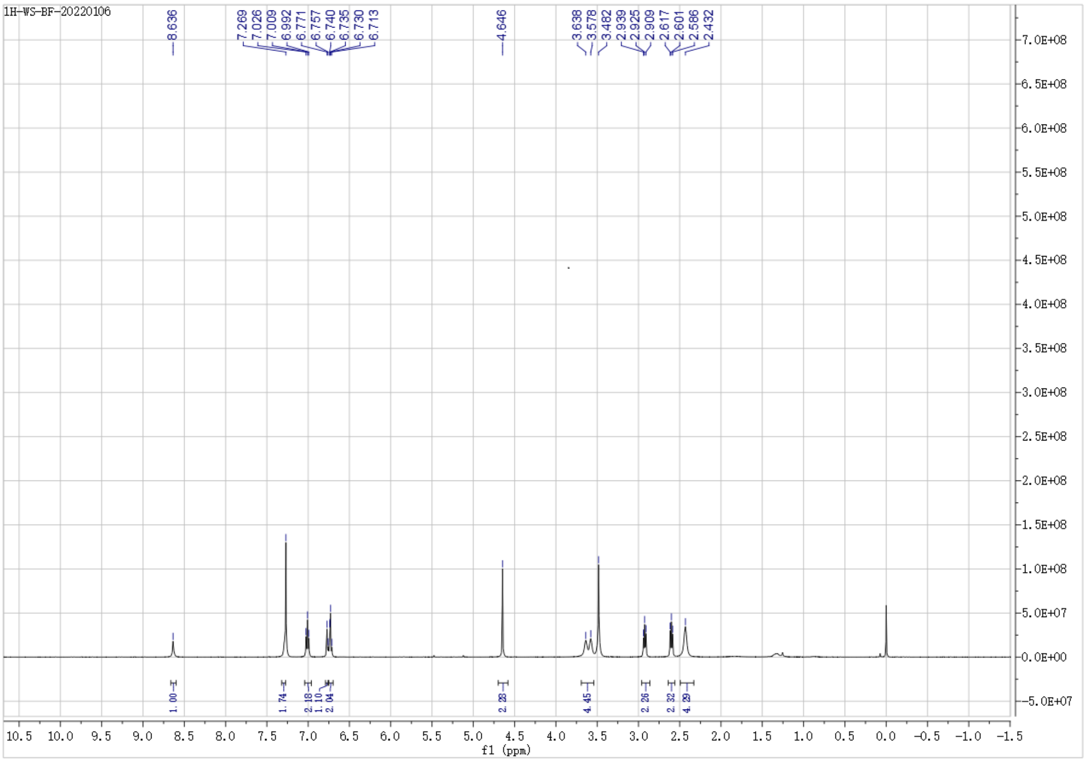

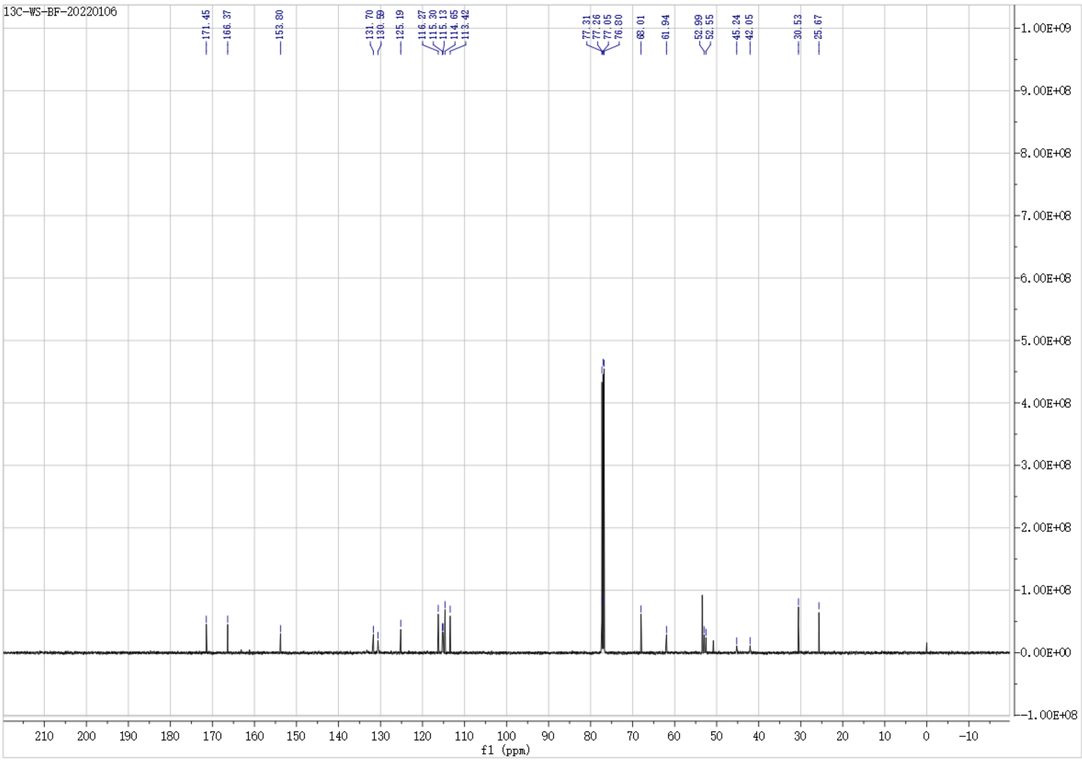

6-(2-(4-(4-Chlorobenzyl)piperazin-1-yl)-2-oxoethoxy)-3,4-dihydroquinolin-2(1H)-one (6c)

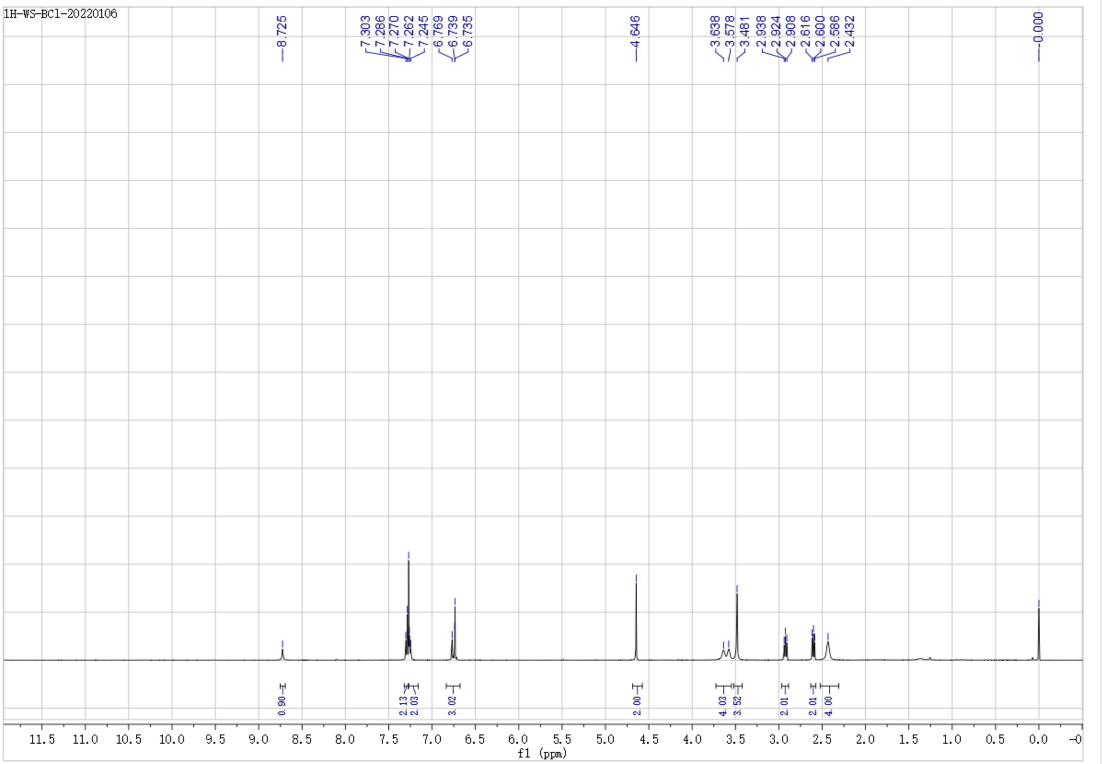

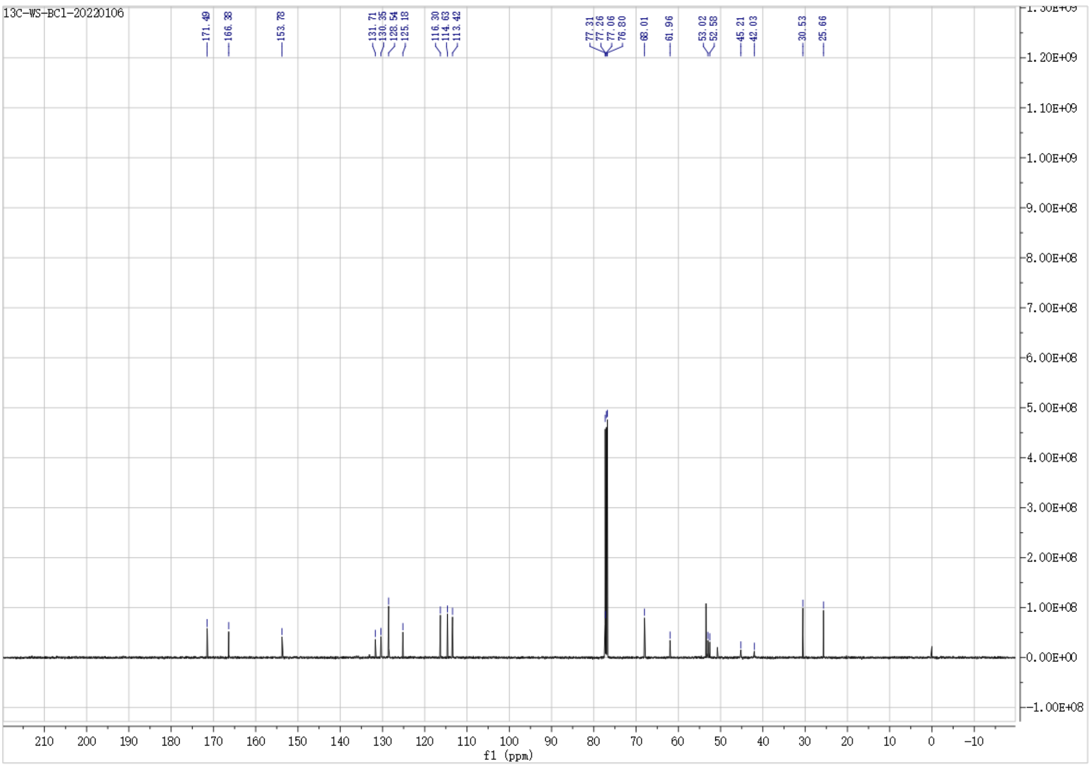

6-(2-Oxo-2-(4-(4-(trifluoromethyl)benzyl)piperazin-1-yl)ethoxy)-3,4-dihydroquinolin-2(1H)-one (6d)

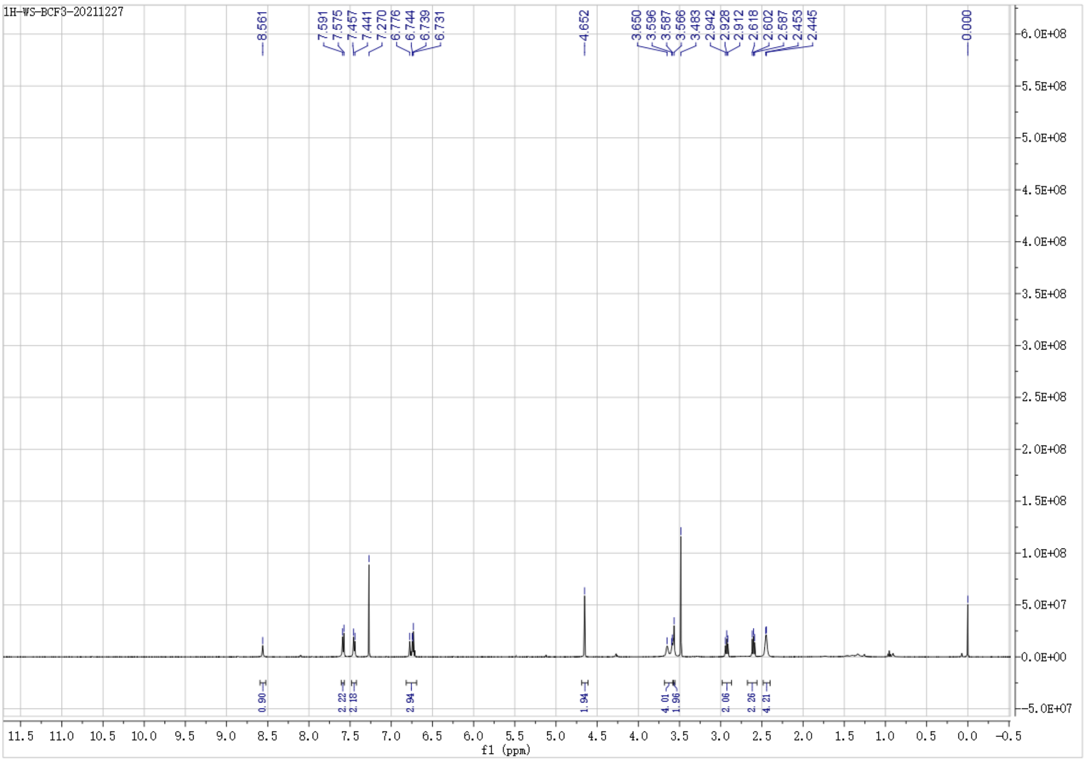

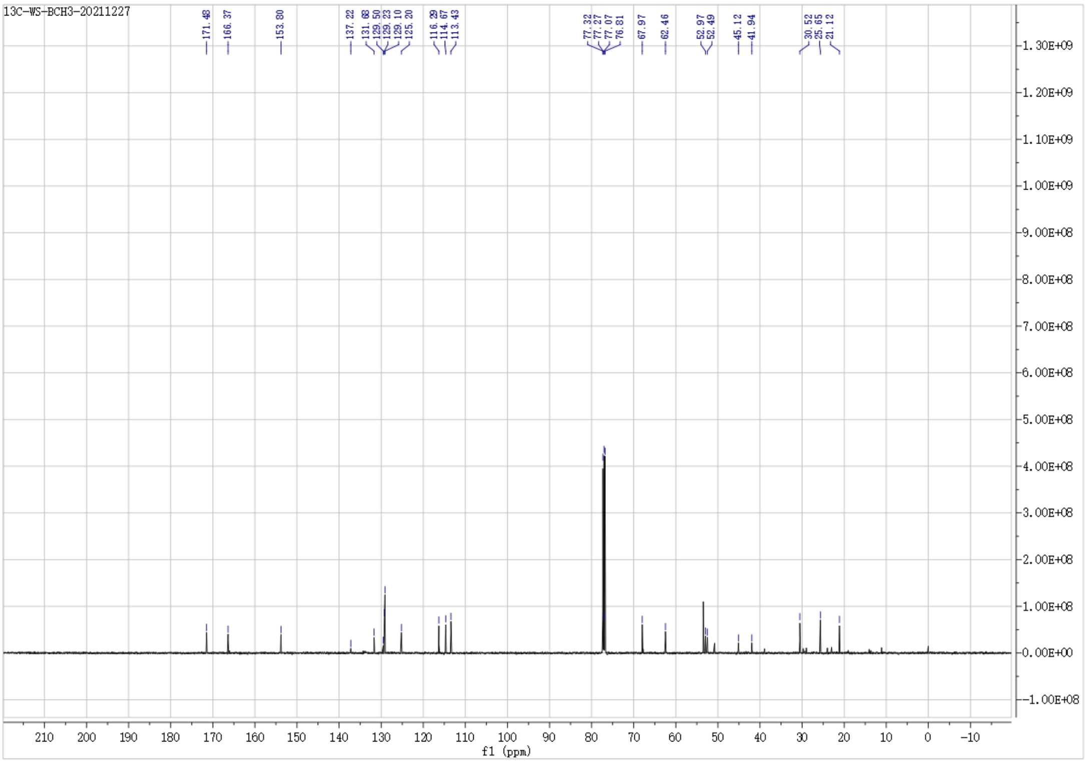

6-(2-(4-(4-Methylbenzyl)piperazin-1-yl)-2-oxoethoxy)-3,4-dihydroquinolin-2(1H)-one (6e)

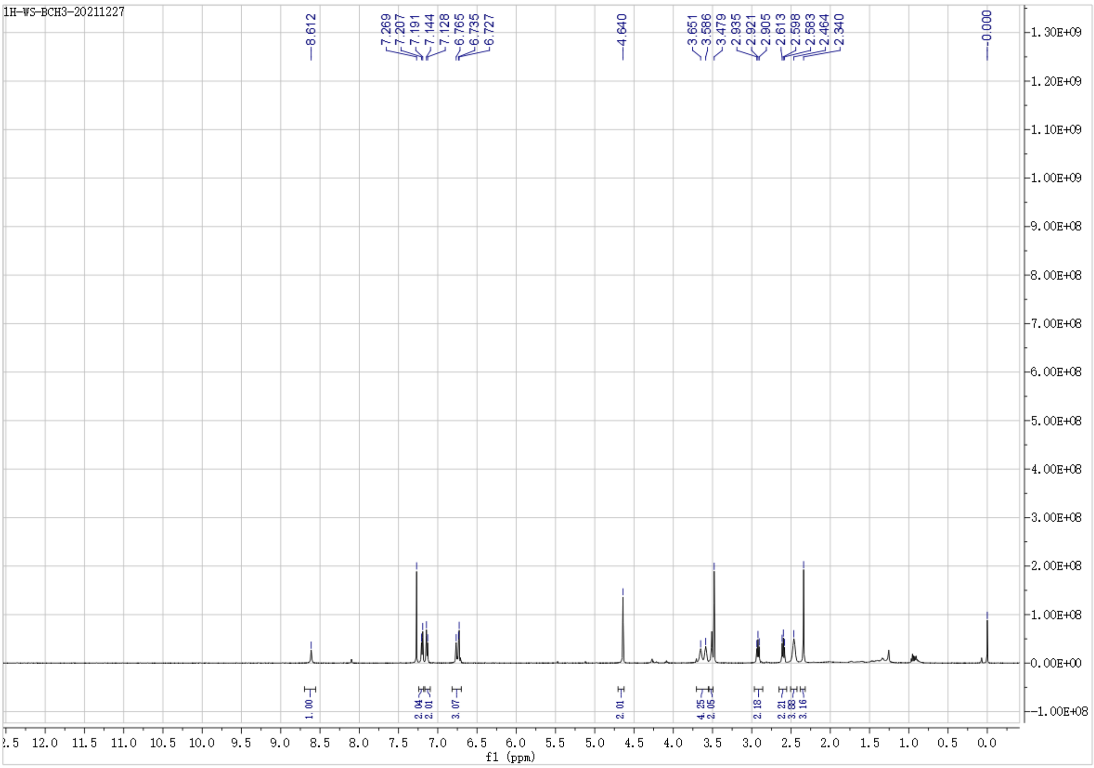

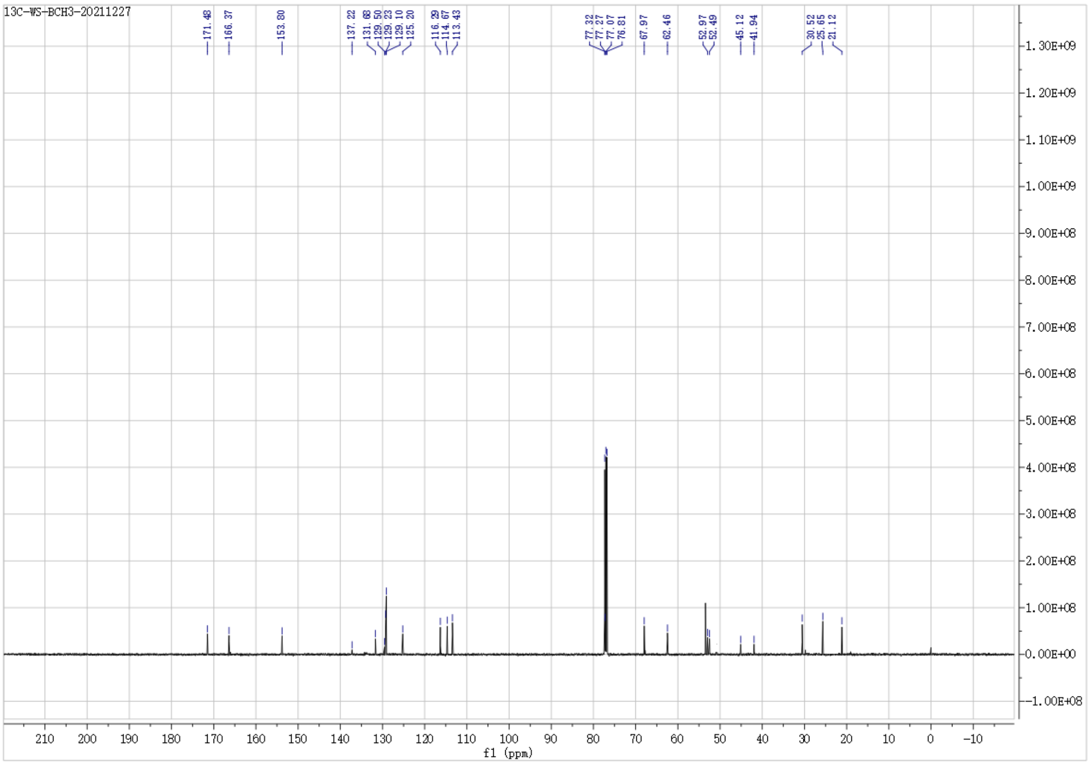

6-(2-(4-Butylpiperazin-1-yl)-2-oxoethoxy)-3,4-dihydroquinolin-2(1H)-one (7a)

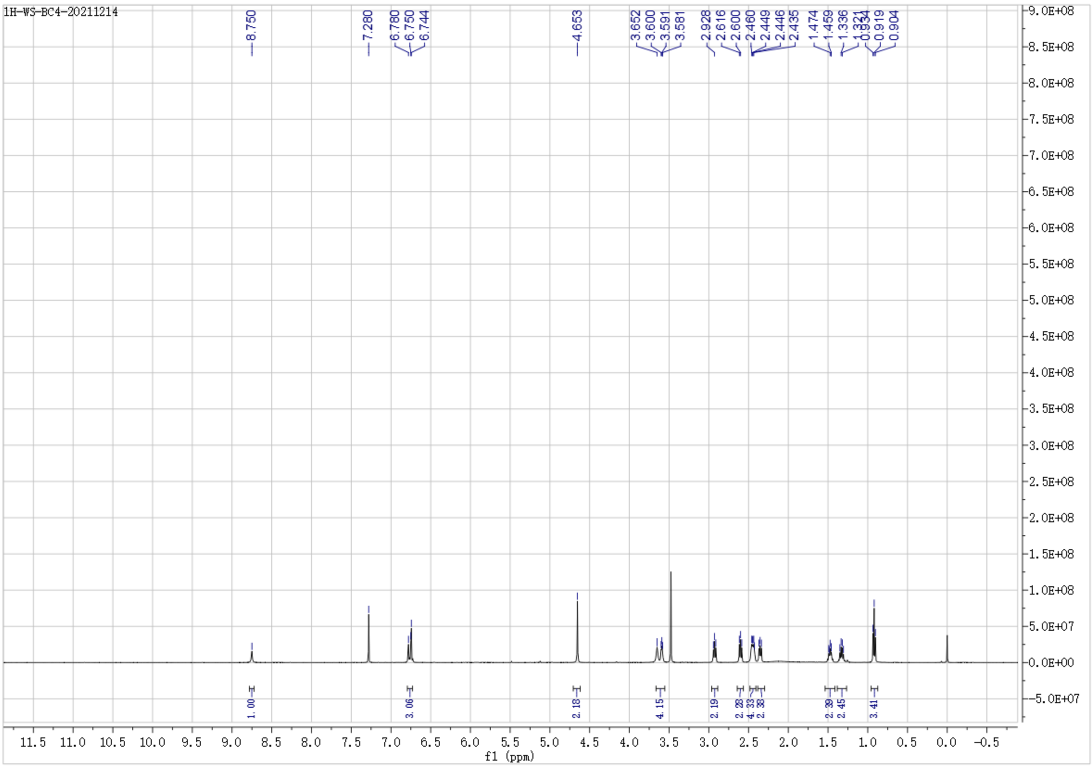

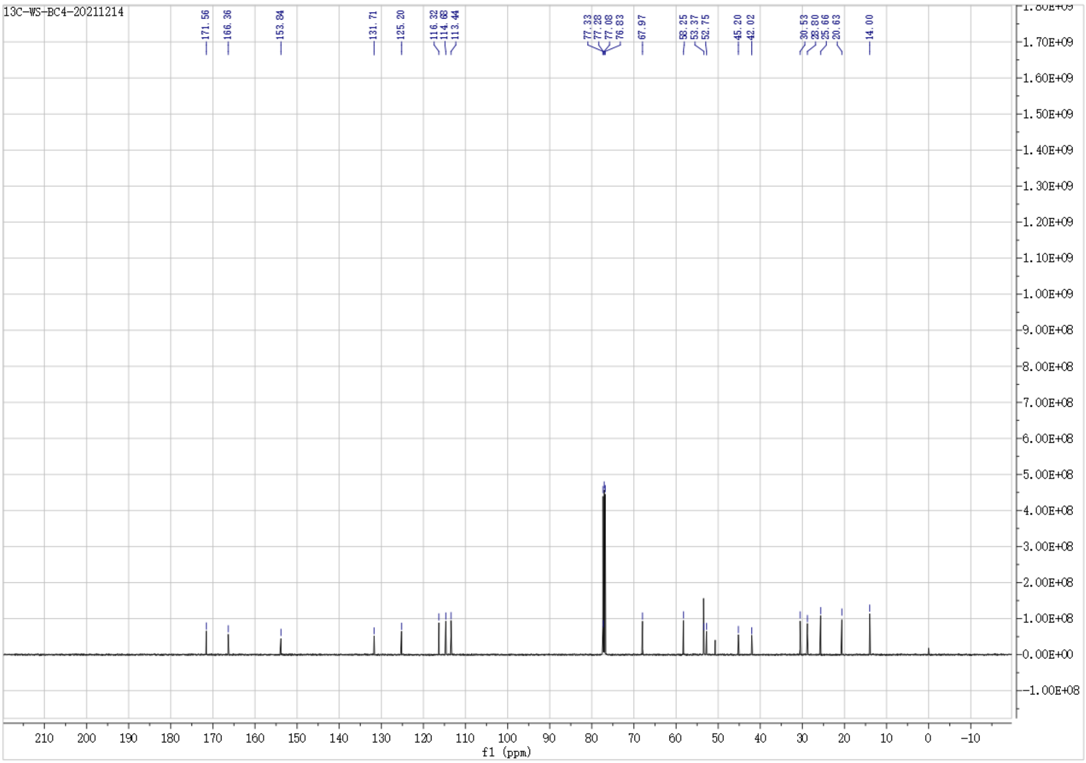

6-(2-Oxo-2-(4-pentylpiperazin-1-yl)ethoxy)-3,4-dihydroquinolin-2(1H)-one (7b)

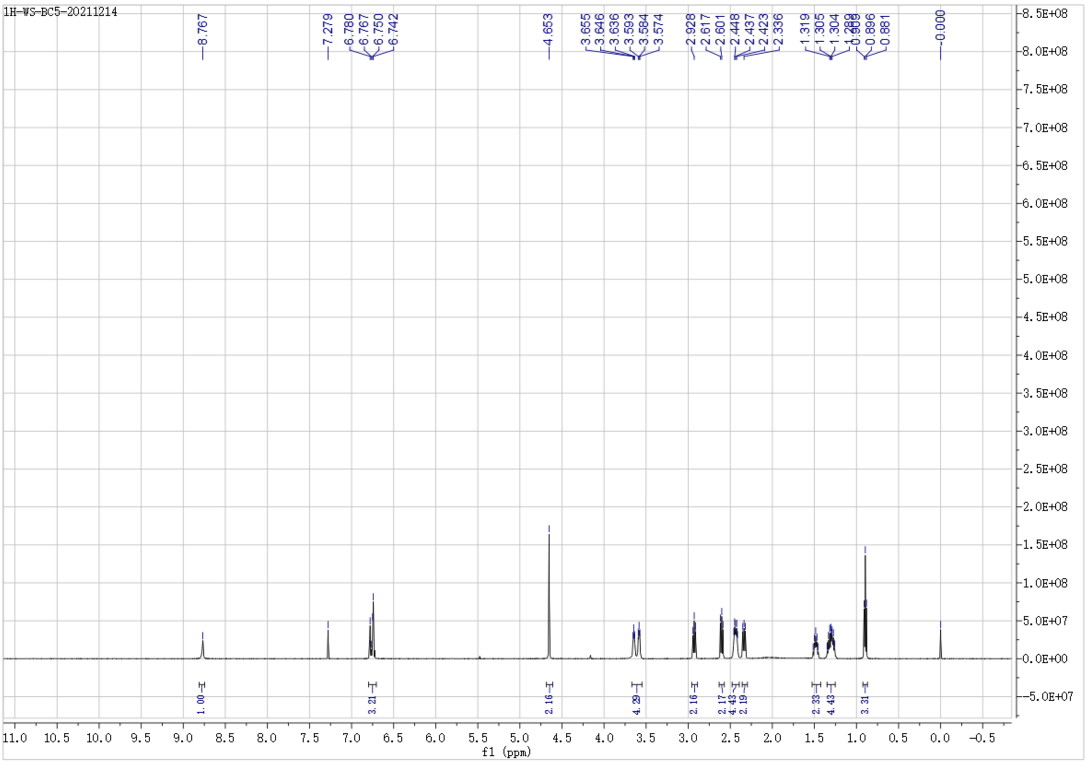

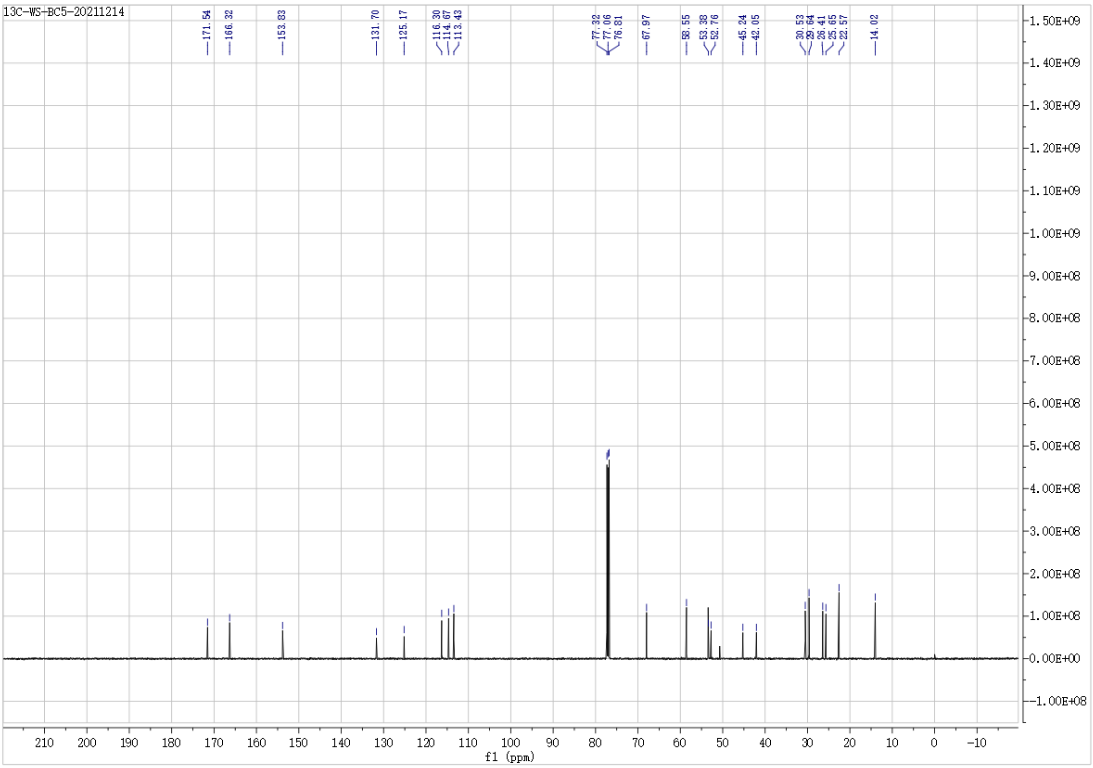

6-(2-(4-Hexylpiperazin-1-yl)-2-oxoethoxy)-3,4-dihydroquinolin-2(1H)-one (7c)

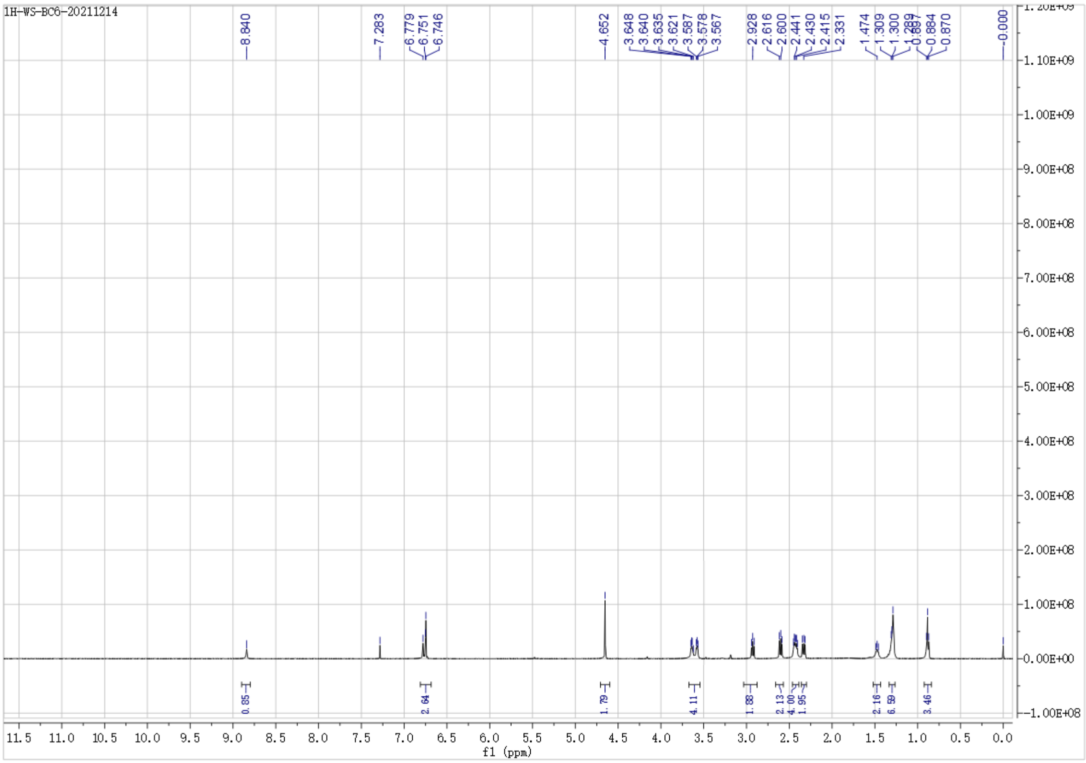

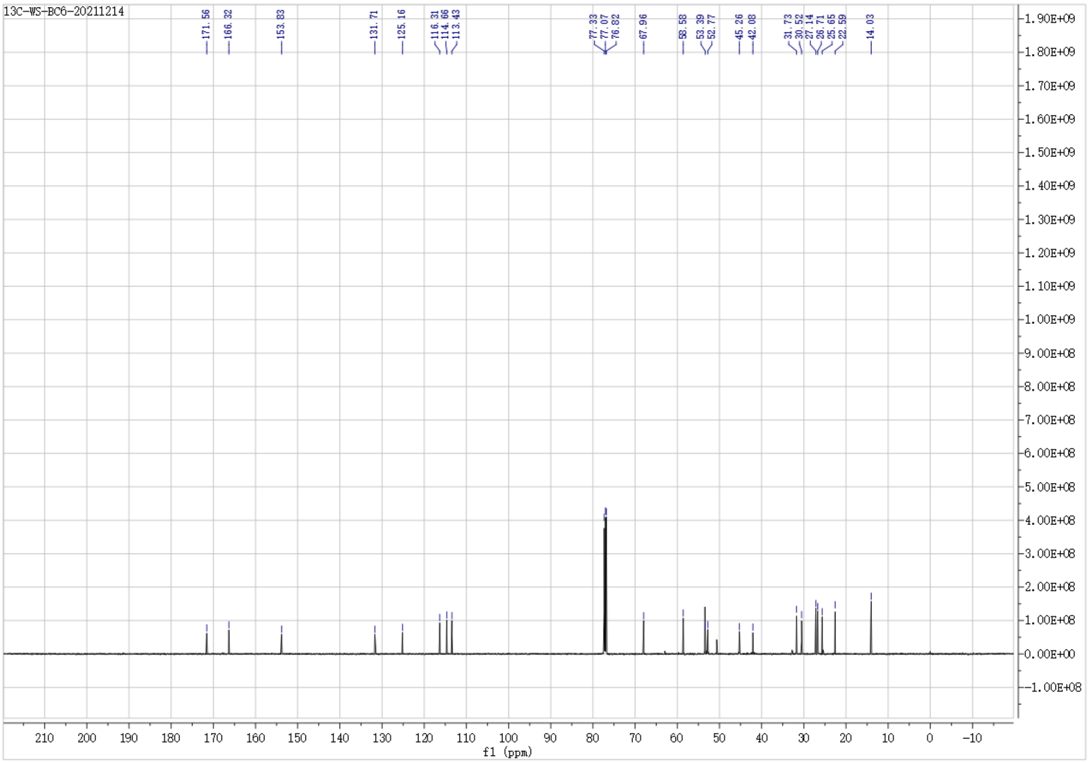

6-(2-(4-Heptylpiperazin-1-yl)-2-oxoethoxy)-3,4-dihydroquinolin-2(1H)-one (7d)

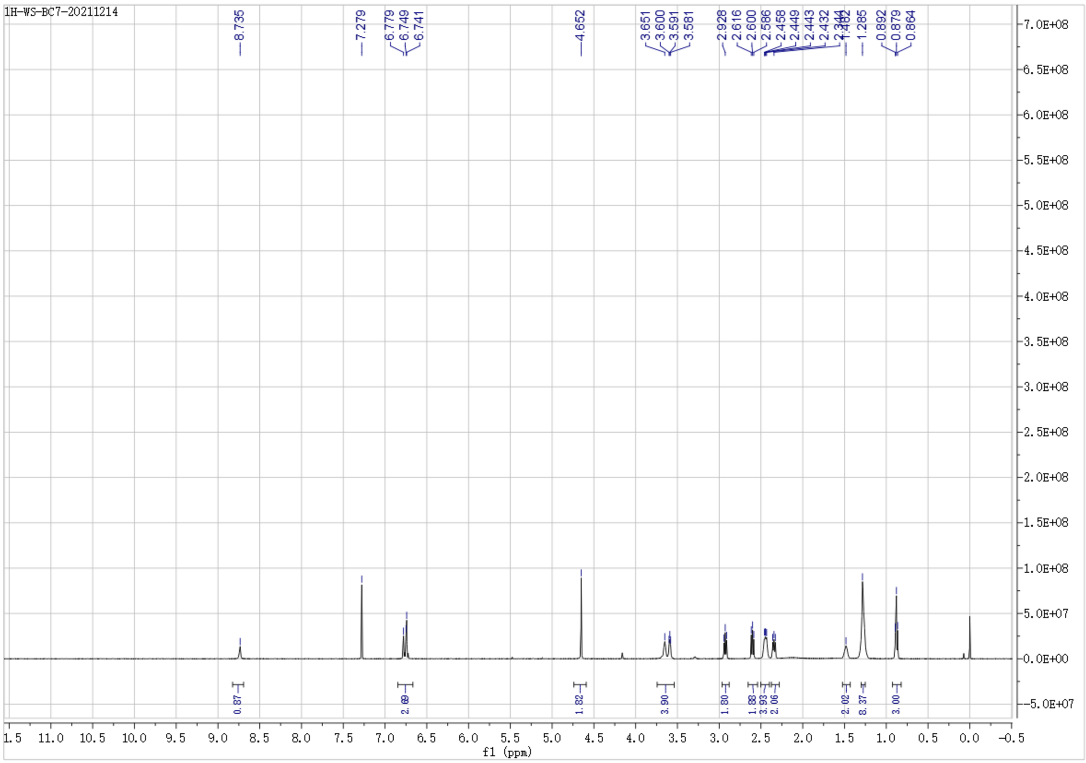

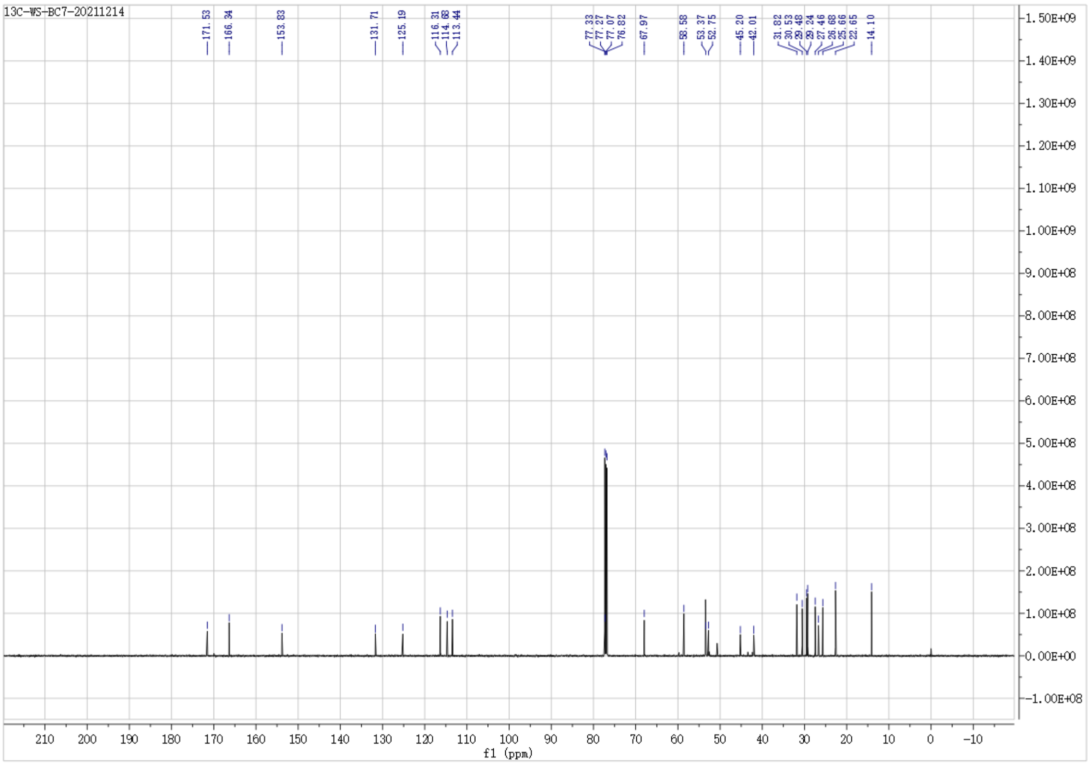

6-(2-(4-Octylpiperazin-1-yl)-2-oxoethoxy)-3,4-dihydroquinolin-2(1H)-one (7e)

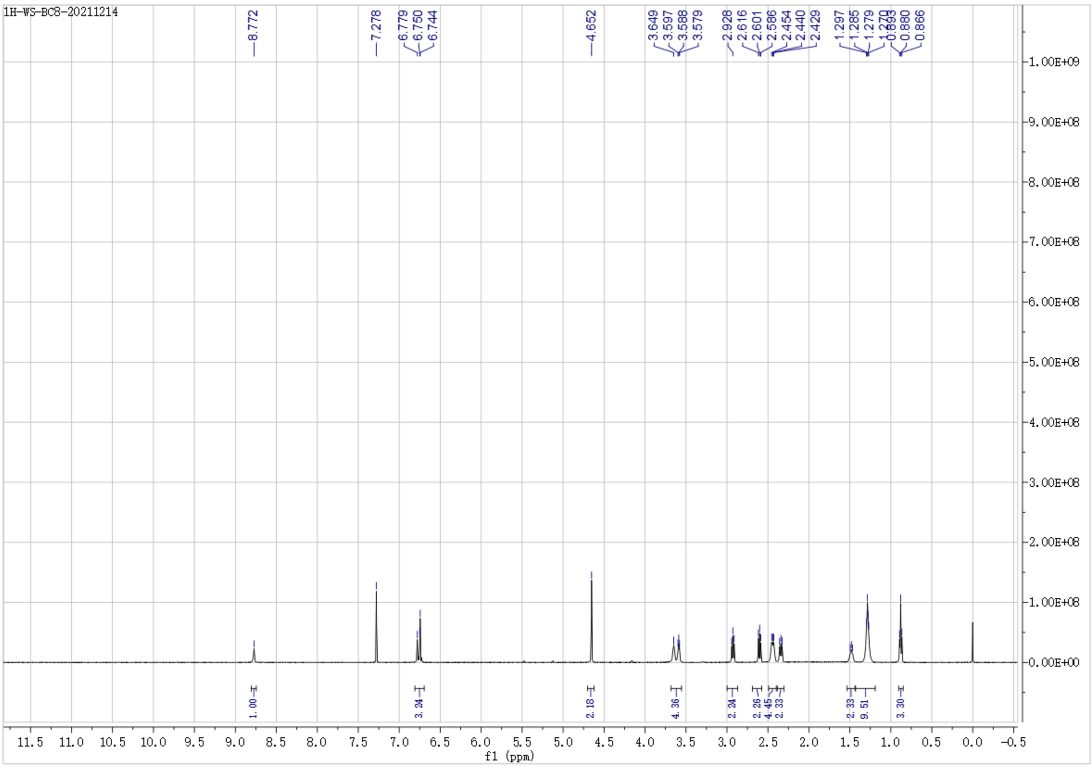

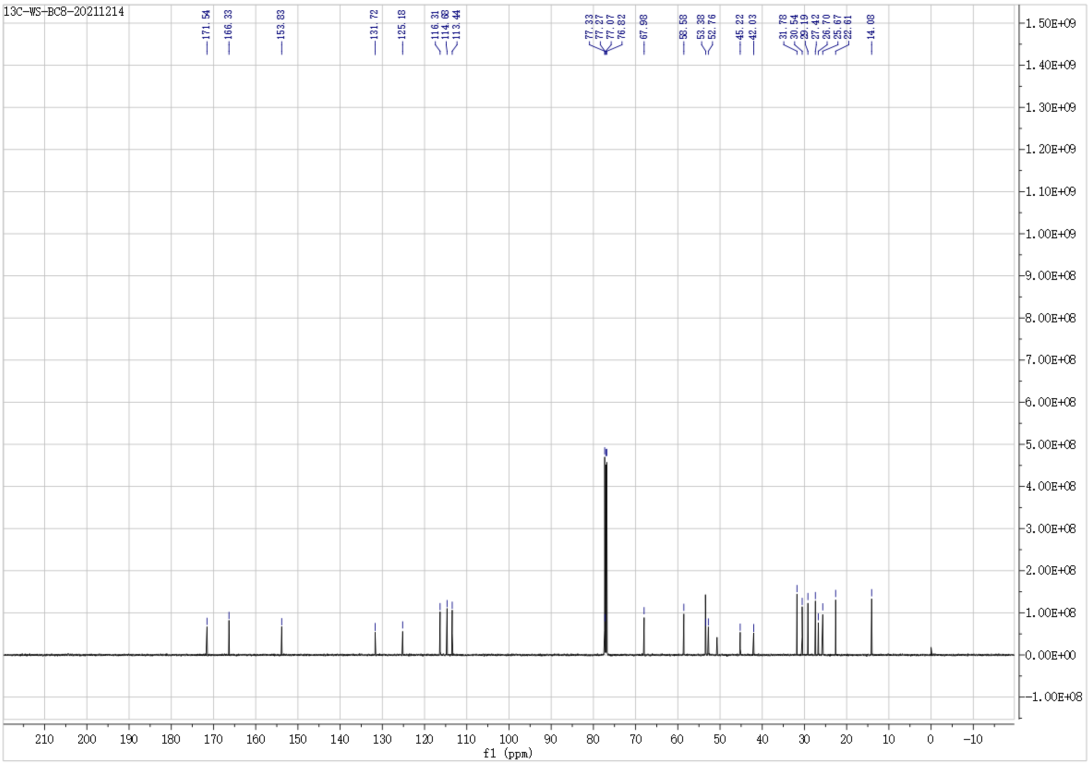

6-(2-(4-Nonylpiperazin-1-yl)-2-oxoethoxy)-3,4-dihydroquinolin-2(1H)-one (7f)

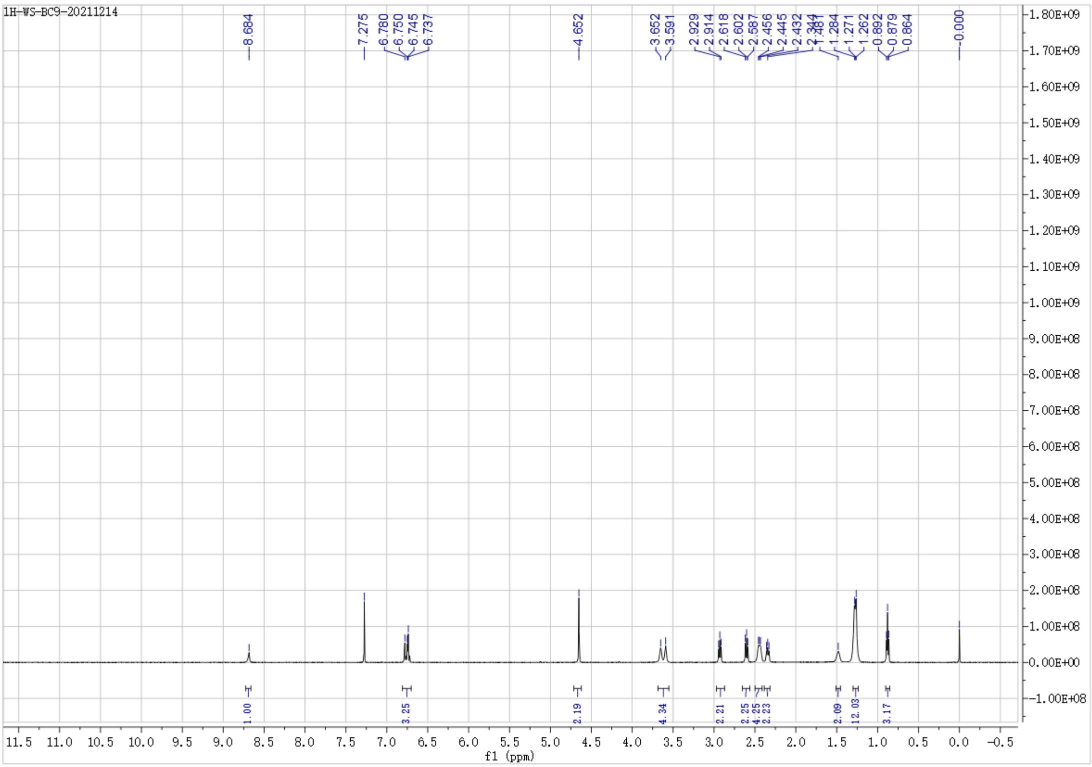

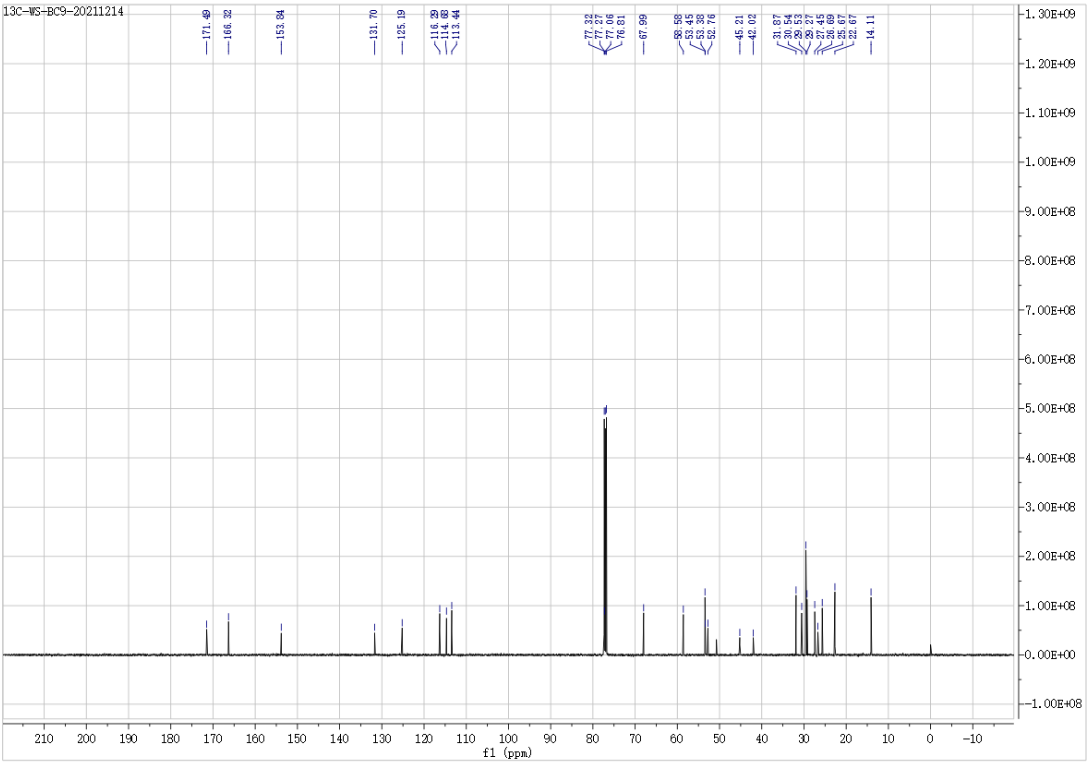

7-(2-(4-Benzylpiperazin-1-yl)-2-oxoethoxy)-3,4-dihydroquinolin-2(1H)-one (9a)

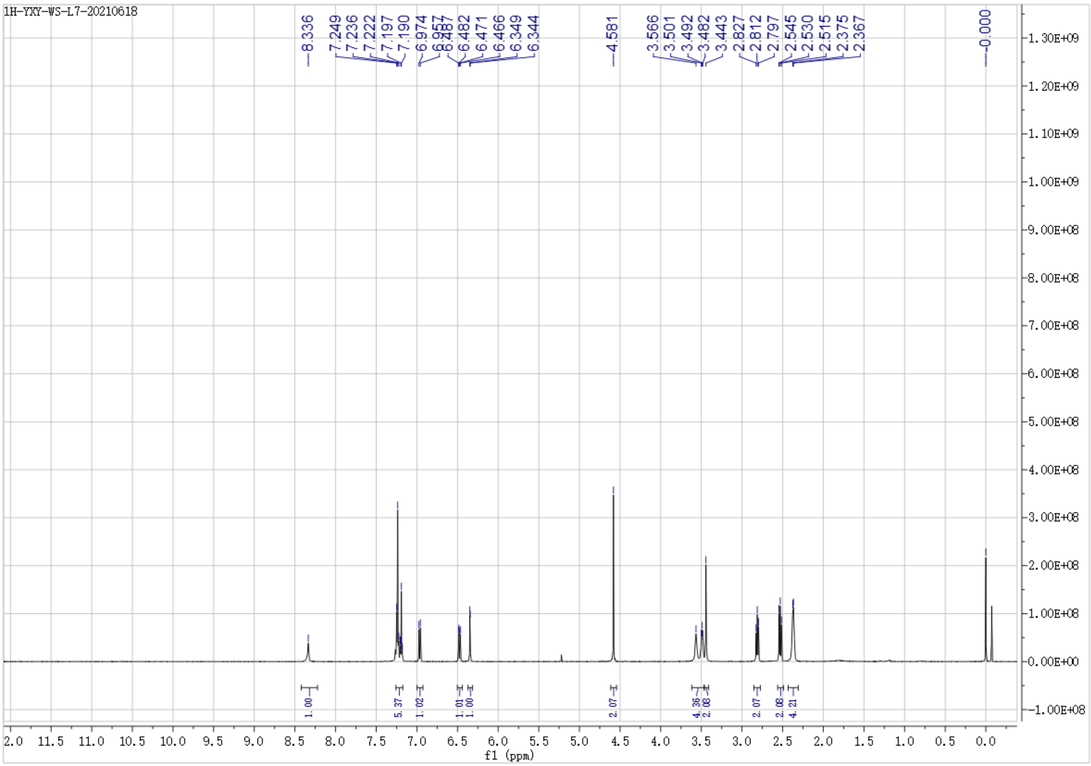

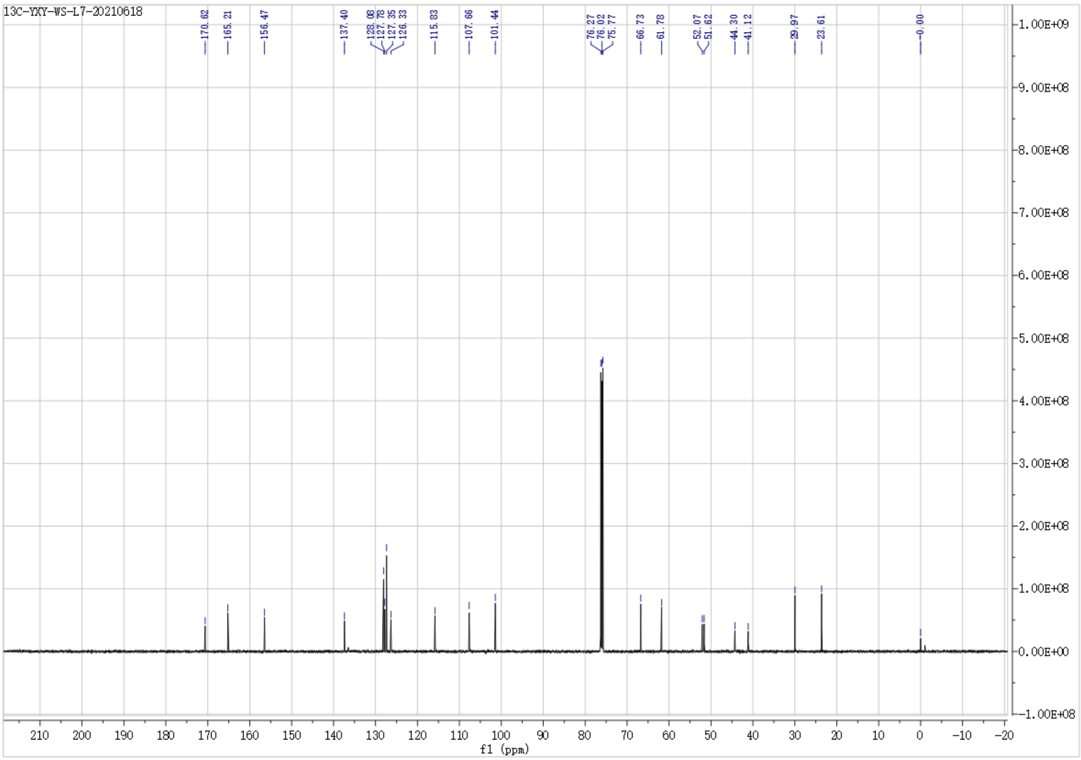

7-(2-(4-(4-Fluorobenzyl)piperazin-1-yl)-2-oxoethoxy)-3,4-dihydroquinolin-2(1H)-one (9b)

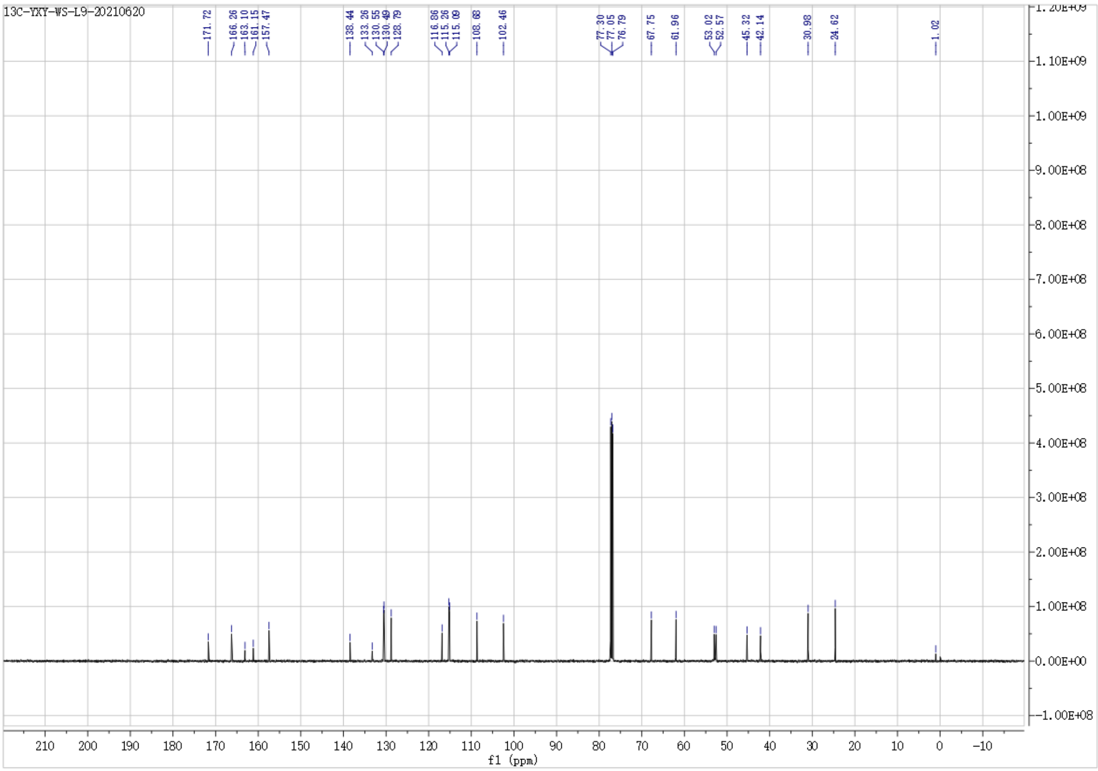

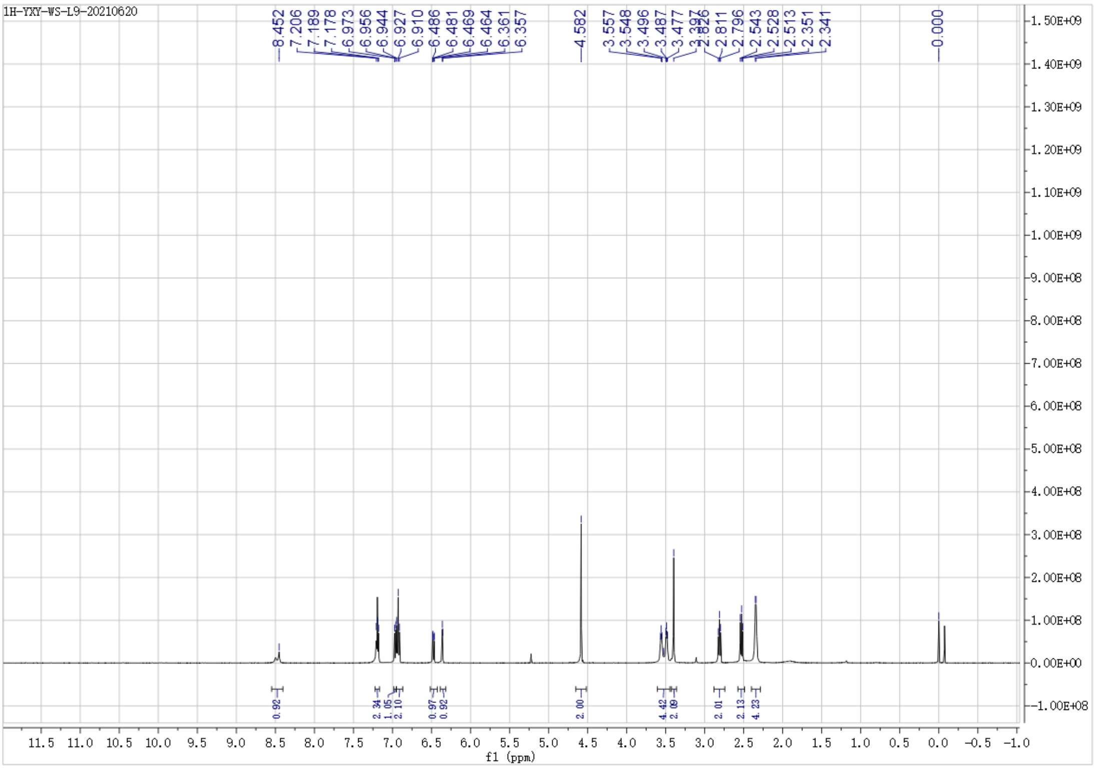

7-(2-(4-(4-Chlorobenzyl)piperazin-1-yl)-2-oxoethoxy)-3,4-dihydroquinolin-2(1H)-one (9c)

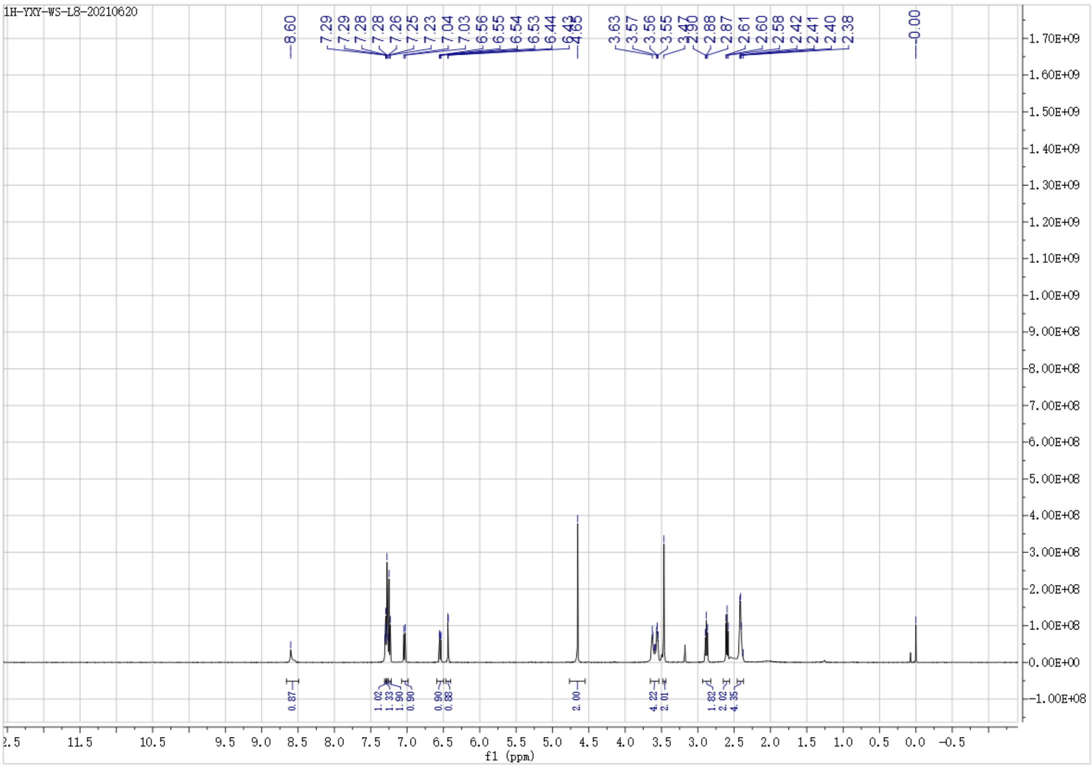

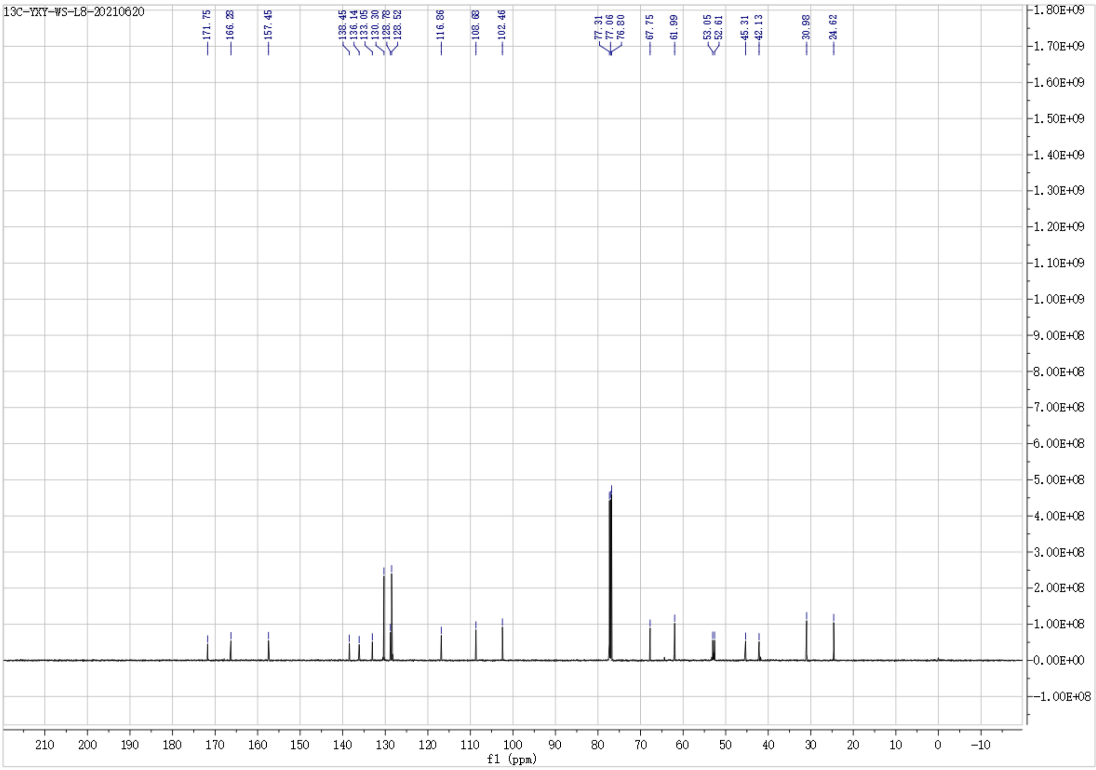

7-(2-Oxo-2-(4-(4-(trifluoromethyl)benzyl)piperazin-1-yl)ethoxy)-3,4-dihydroquinolin-2(1H)-one (9d)

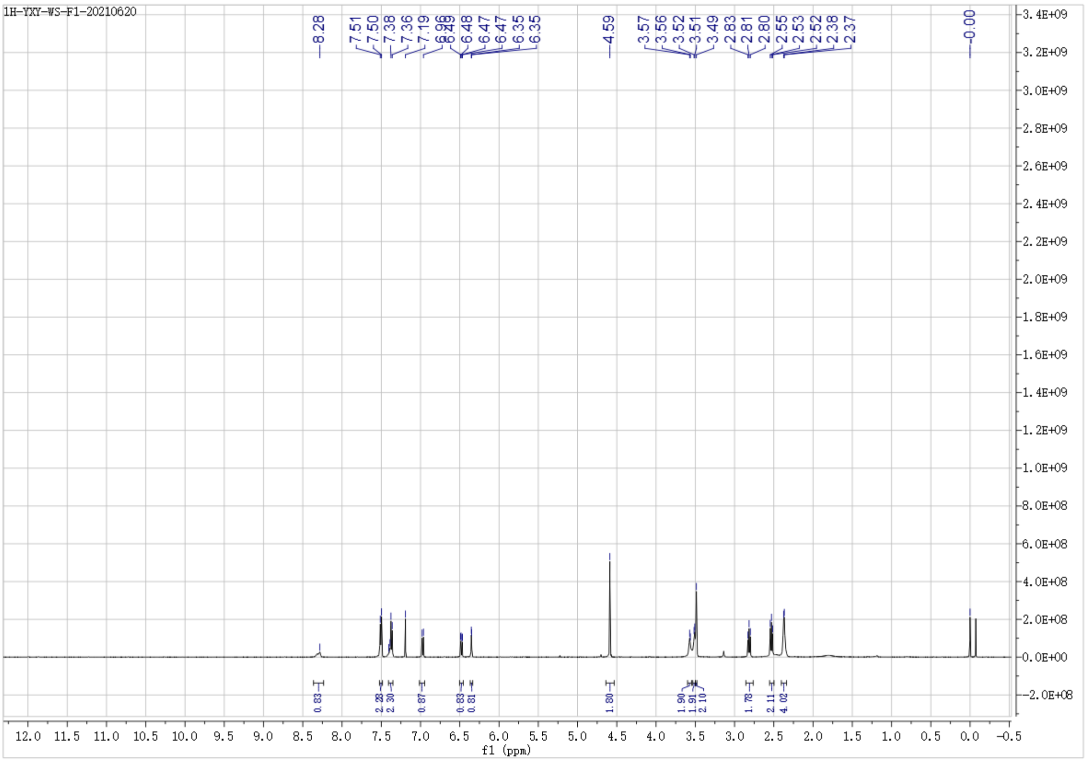

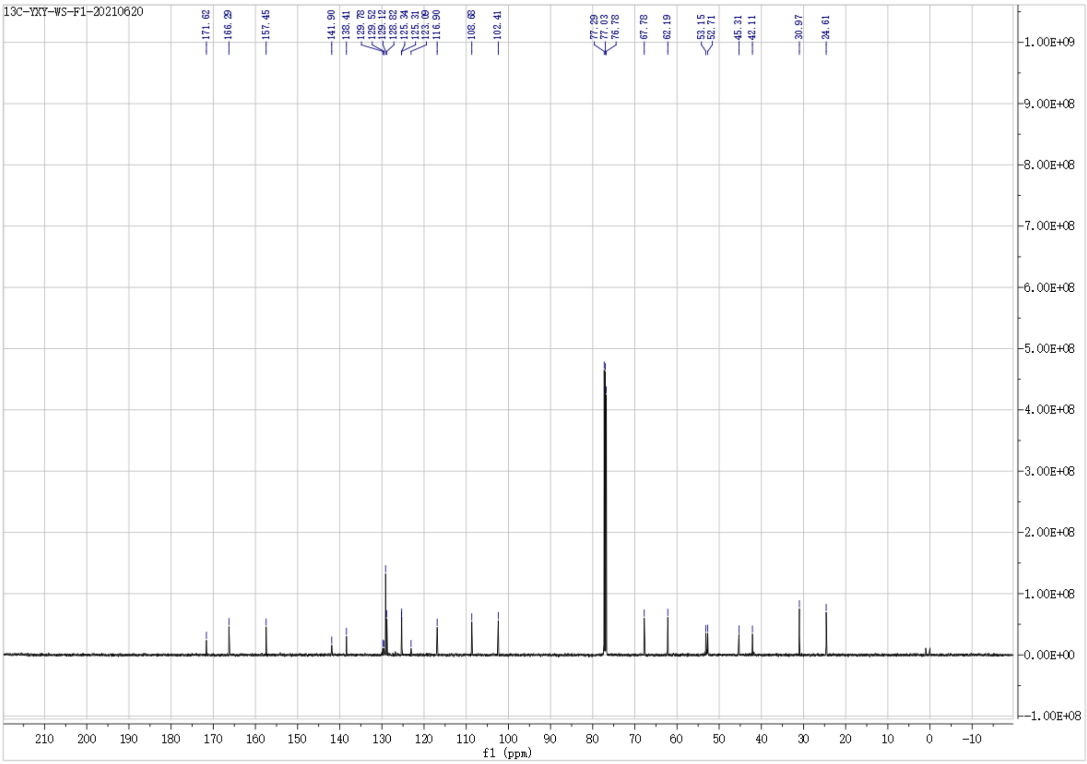

7-(2-(4-(4-Methylbenzyl)piperazin-1-yl)-2-oxoethoxy)-3,4-dihydroquinolin-2(1H)-one (9e)

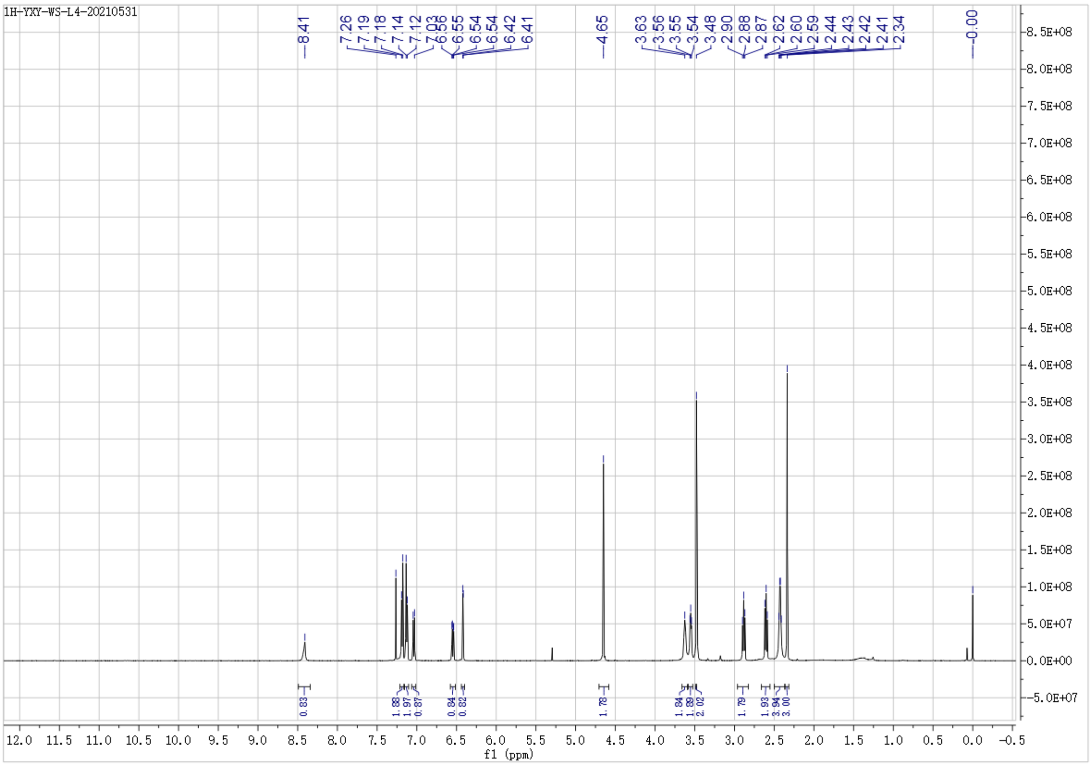

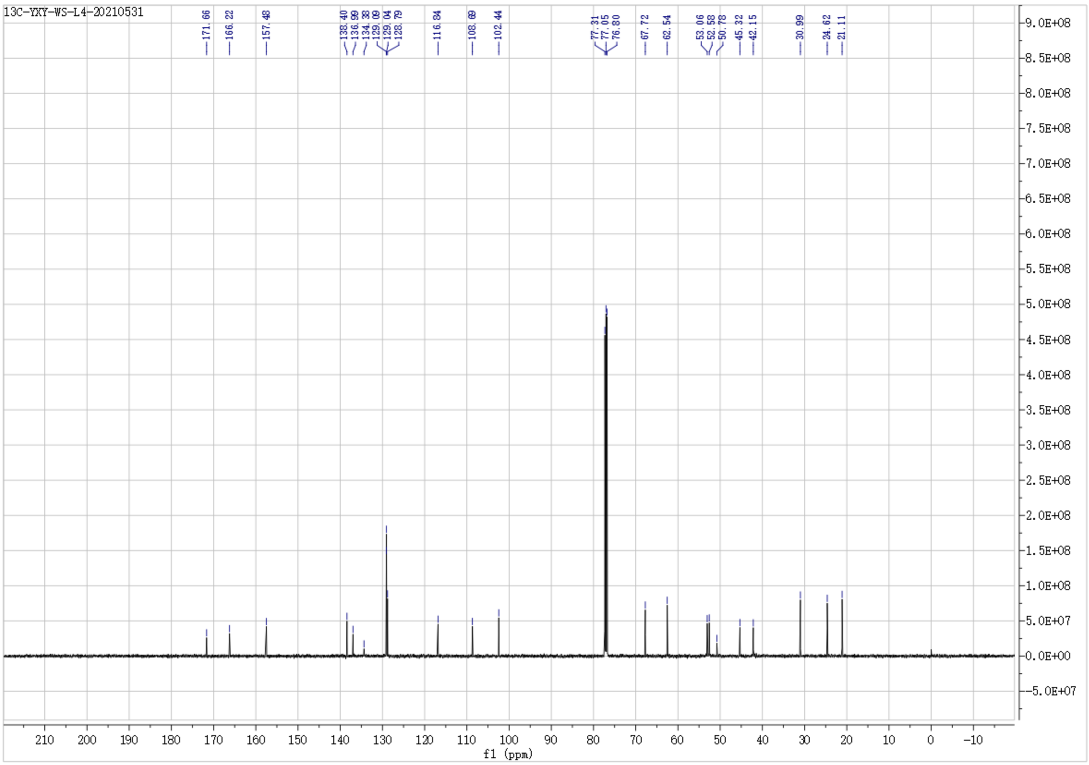

7-(2-(4-Butylpiperazin-1-yl)-2-oxoethoxy)-3,4-dihydroquinolin-2(1H)-one (10a)

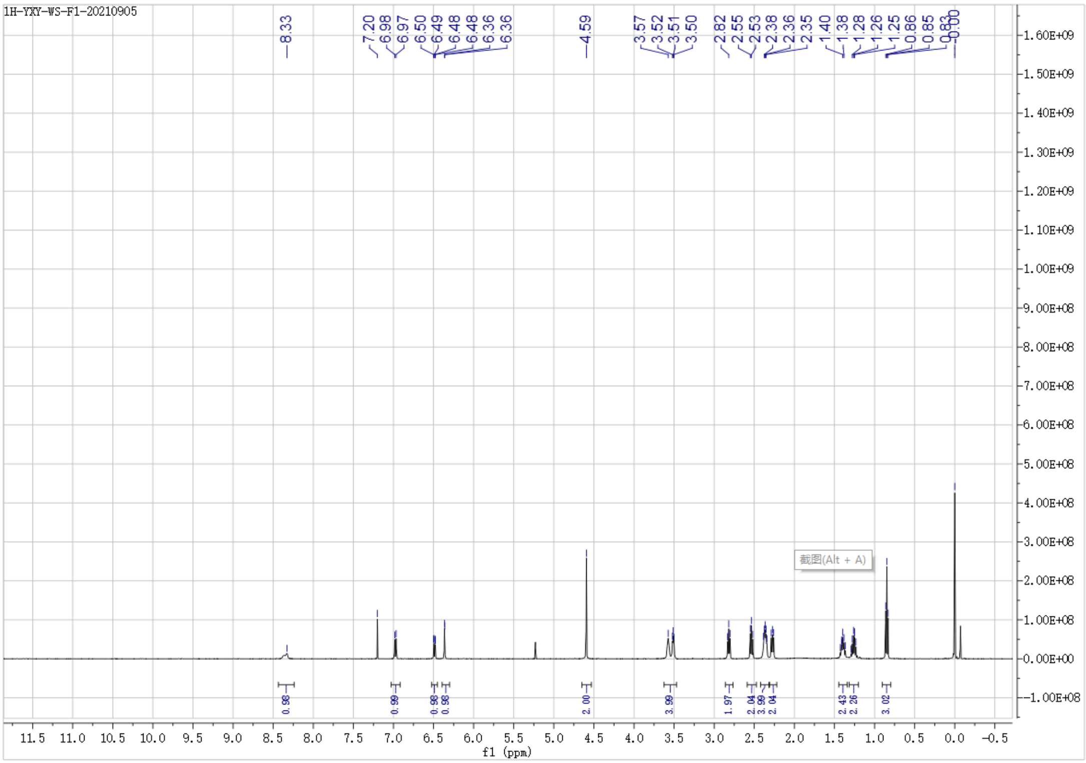

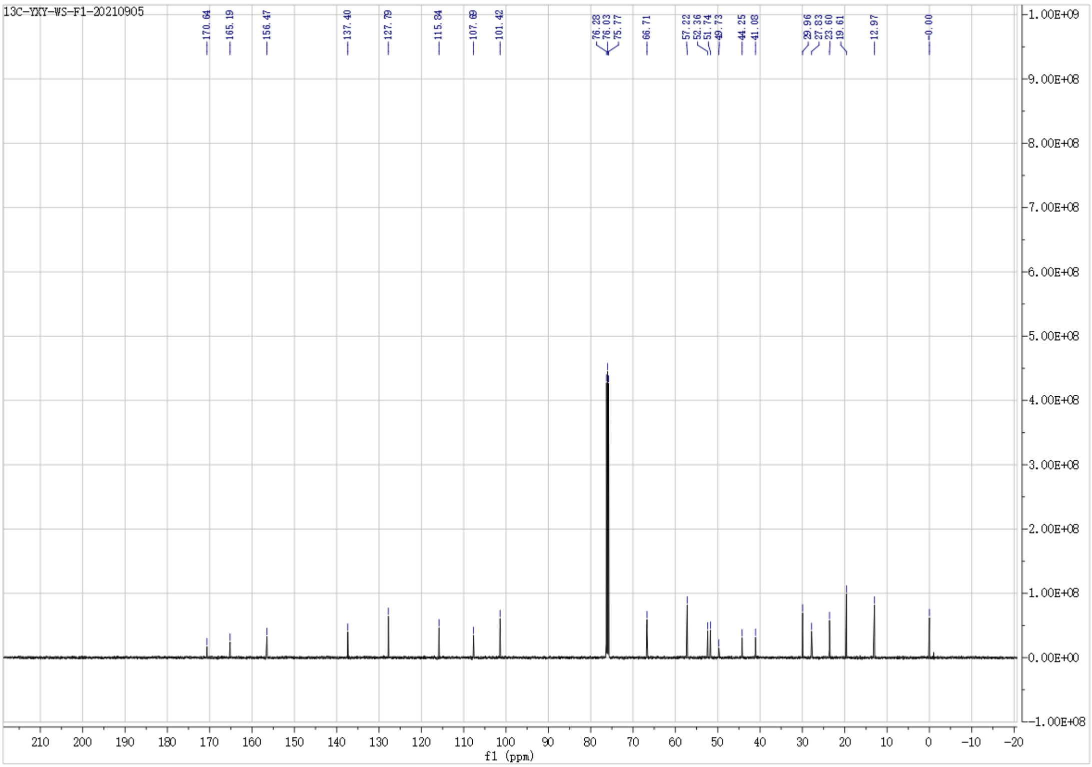

7-(2-Oxo-2-(4-pentylpiperazin-1-yl)ethoxy)-3,4-dihydroquinolin-2(1H)-one (10b)

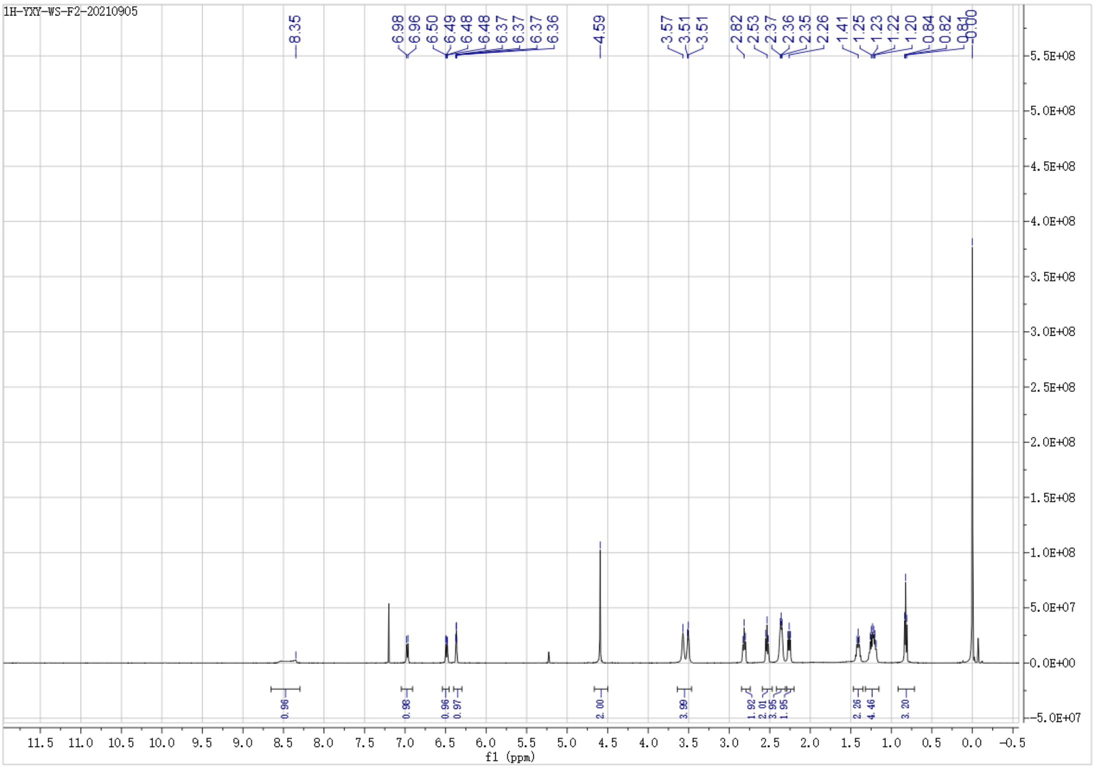

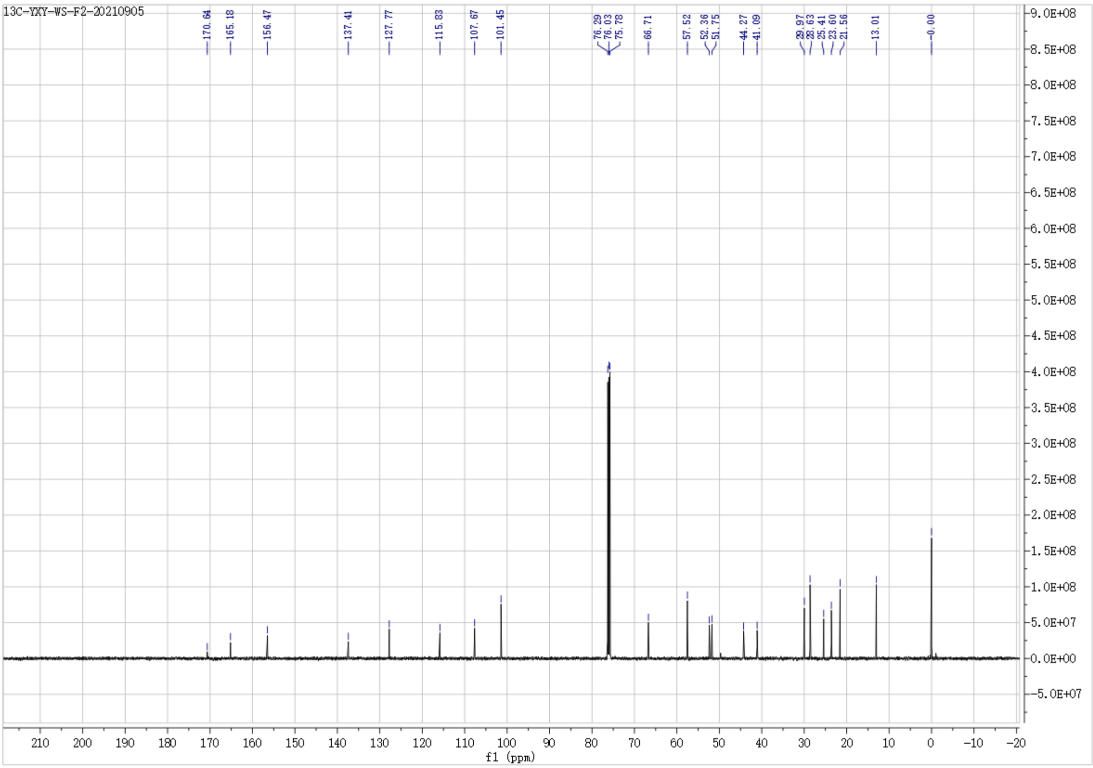

7-(2-(4-Hexylpiperazin-1-yl)-2-oxoethoxy)-3,4-dihydroquinolin-2(1H)-one (10c)

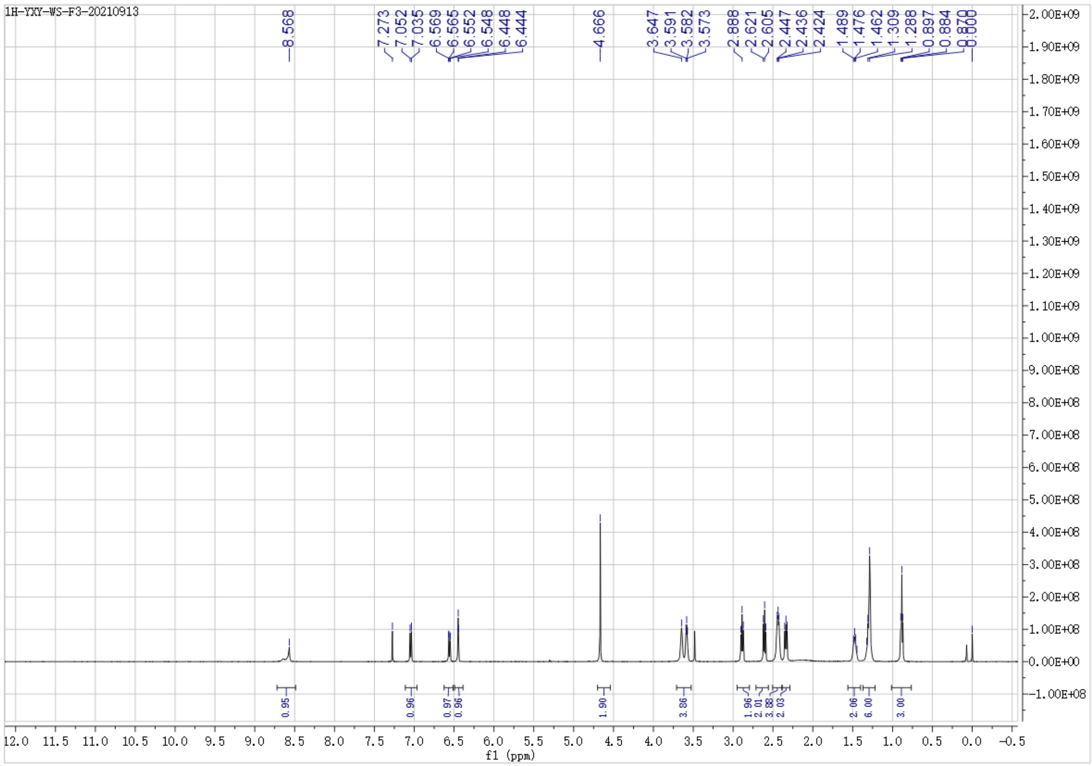

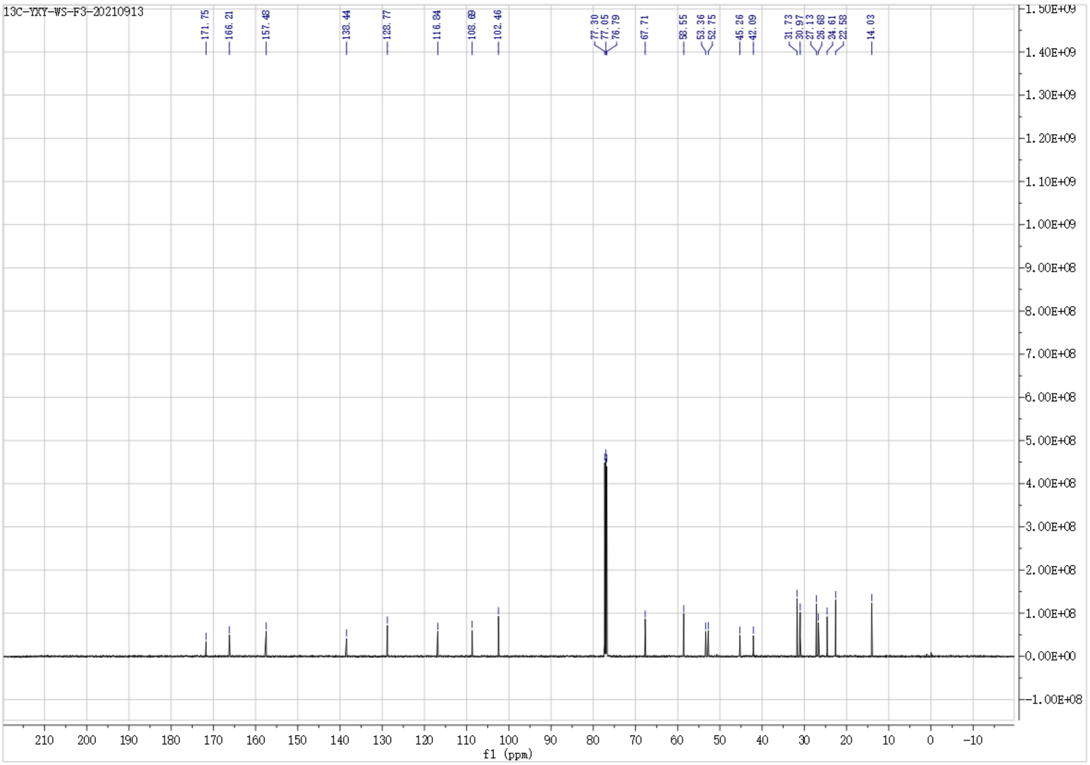

7-(2-(4-Heptylpiperazin-1-yl)-2-oxoethoxy)-3,4-dihydroquinolin-2(1H)-one (10d)

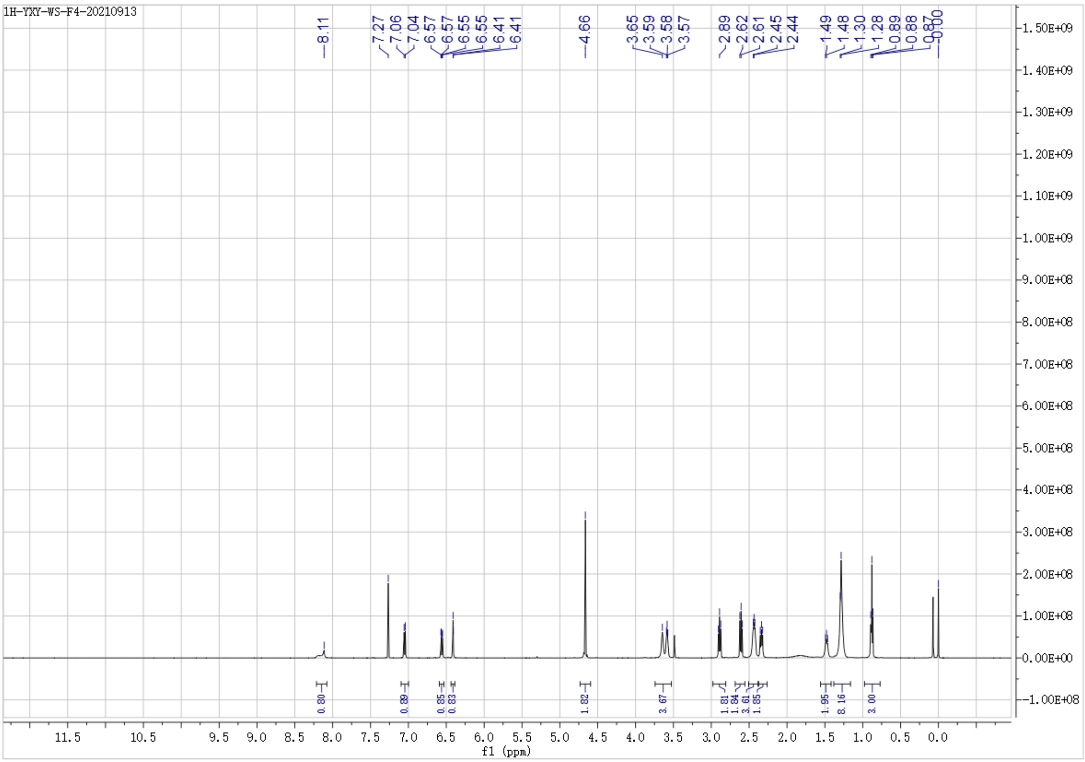

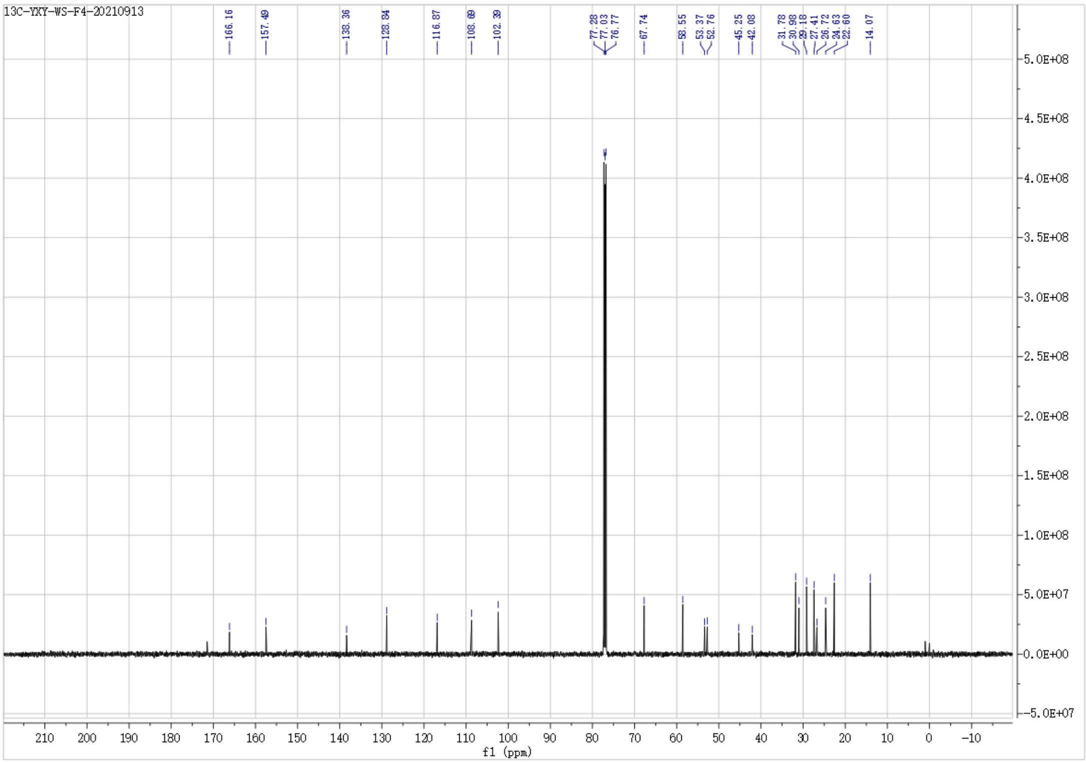

7-(2-(4-Octylpiperazin-1-yl)-2-oxoethoxy)-3,4-dihydroquinolin-2(1H)-one (10e)

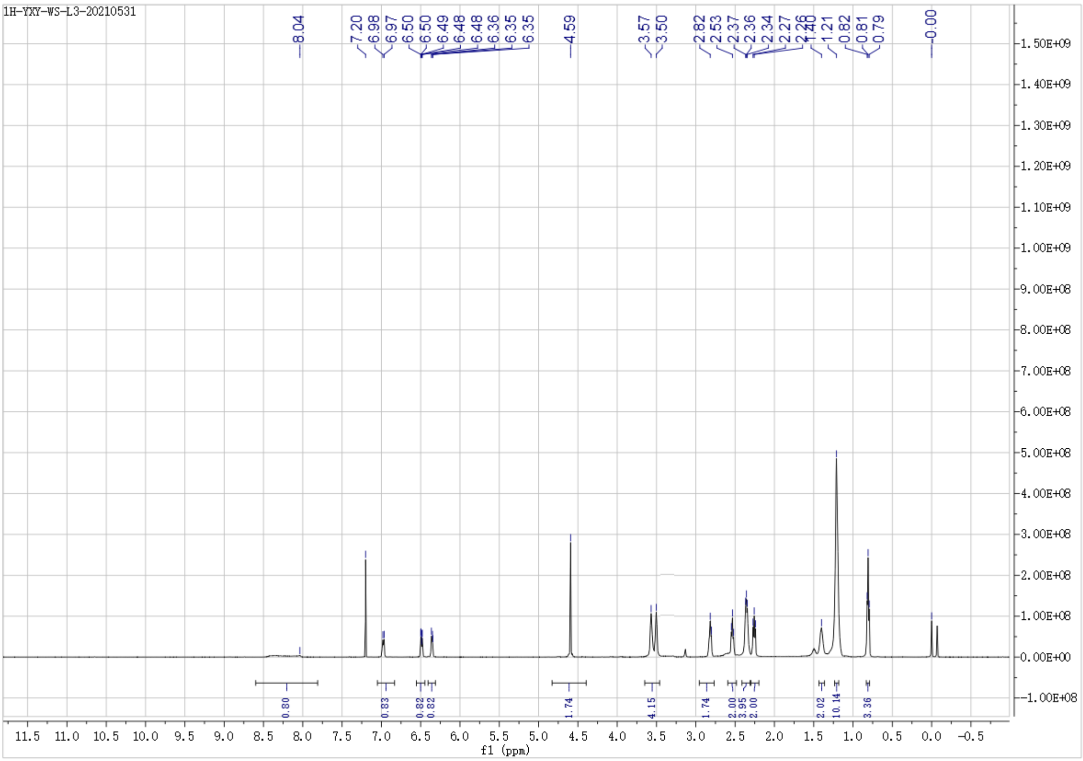

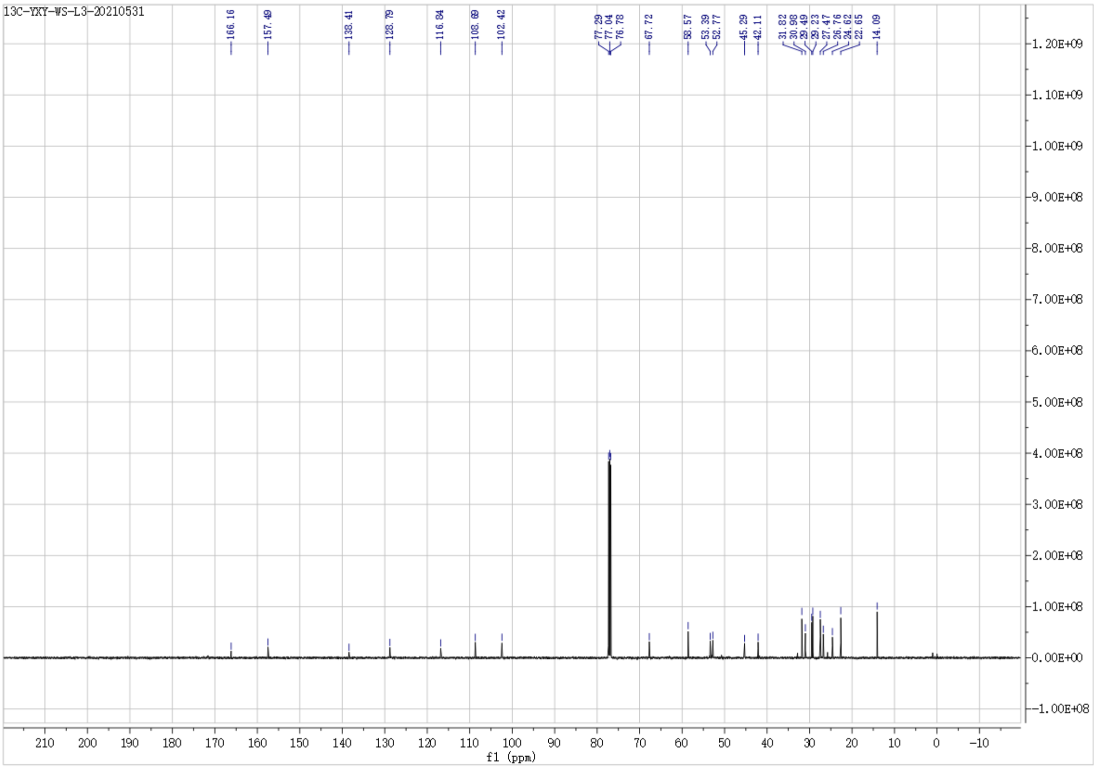

7-(2-(4-Nonylpiperazin-1-yl)-2-oxoethoxy)-3,4-dihydroquinolin-2(1H)-one (10f)

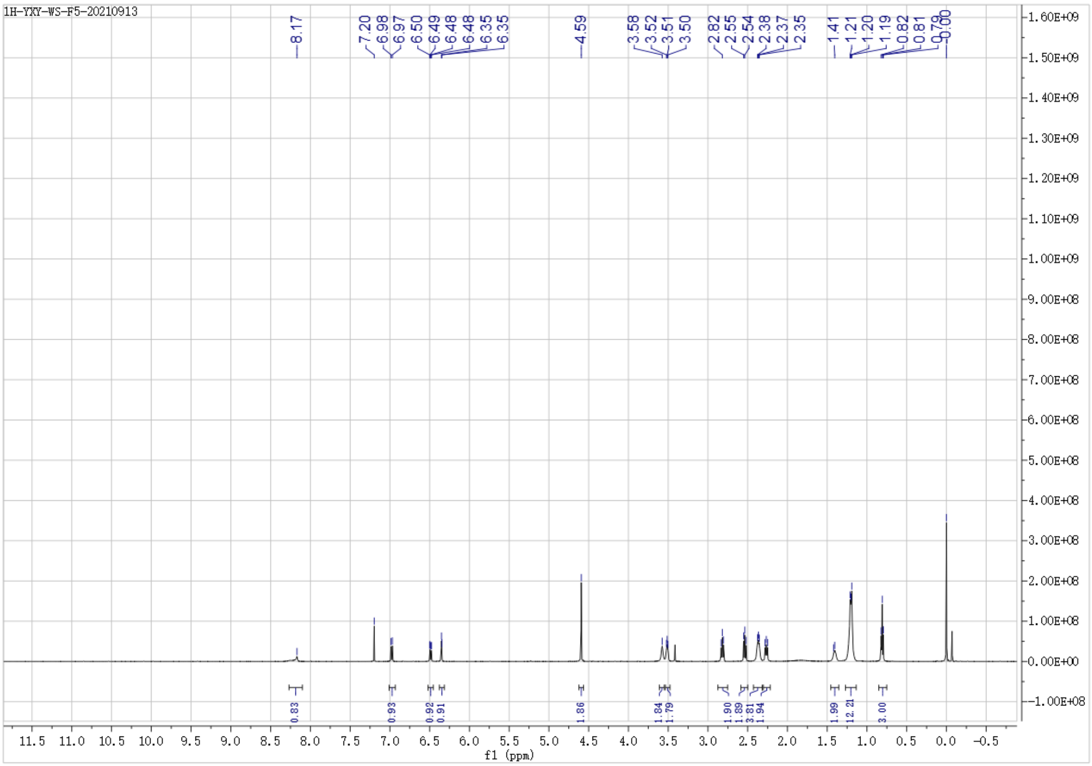

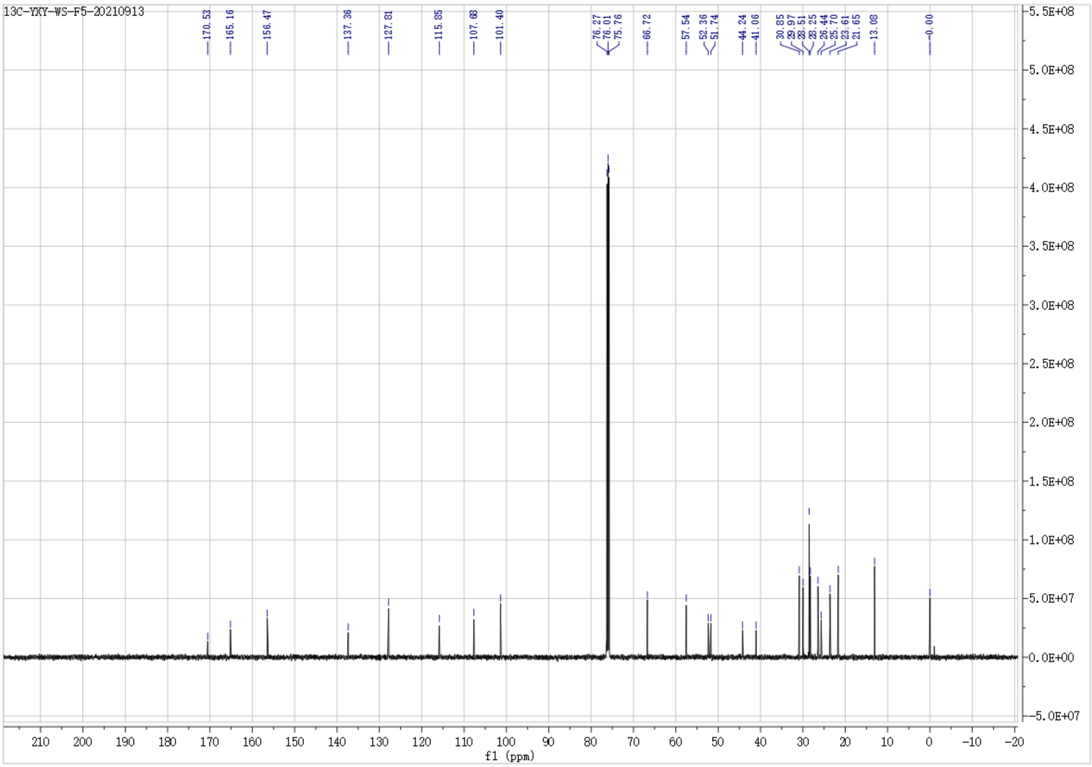

N-(4-(2-(4-benzylpiperazin-1-yl)-2-oxoethoxy)phenyl)acetamide (14a)

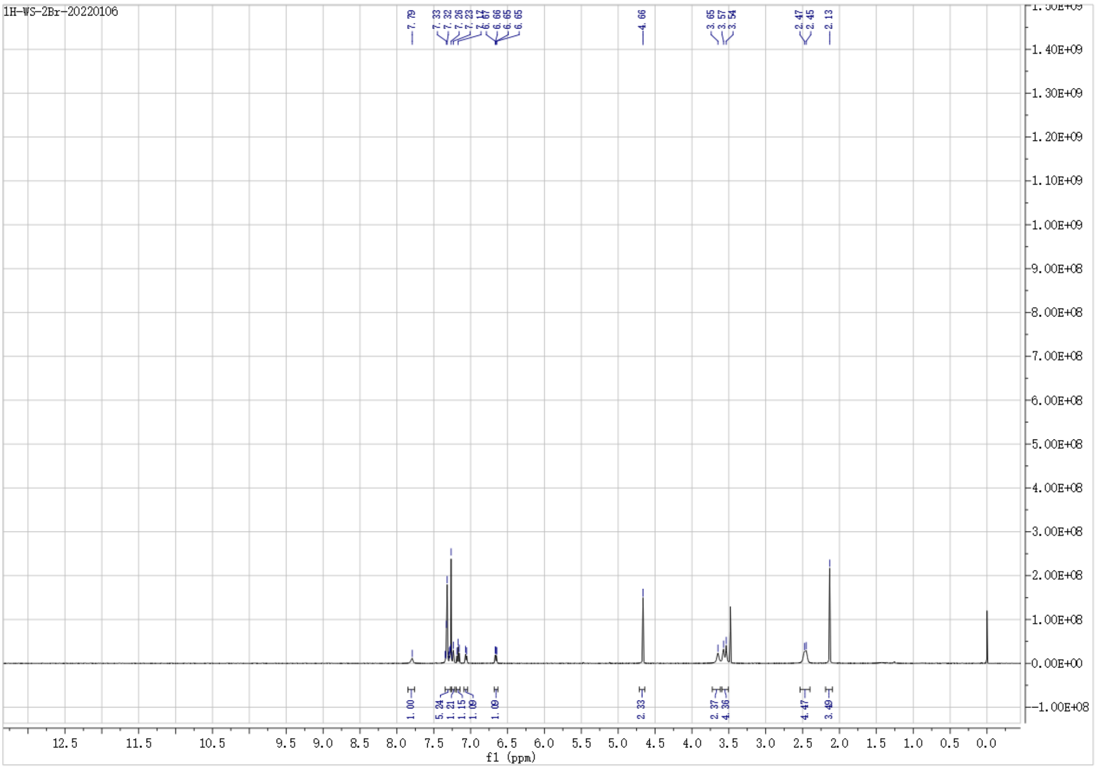

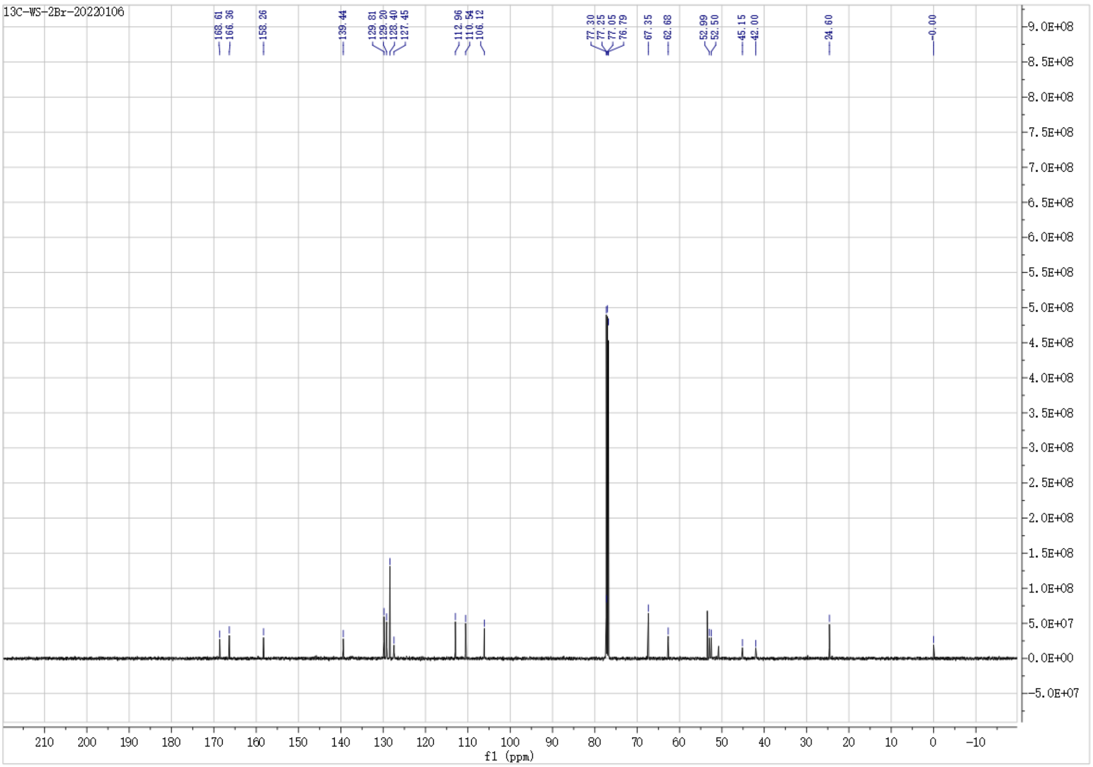

N-(4-(2-(4-(4-fluorobenzyl)piperazin-1-yl)-2-oxoethoxy)phenyl)acetamide (14b)

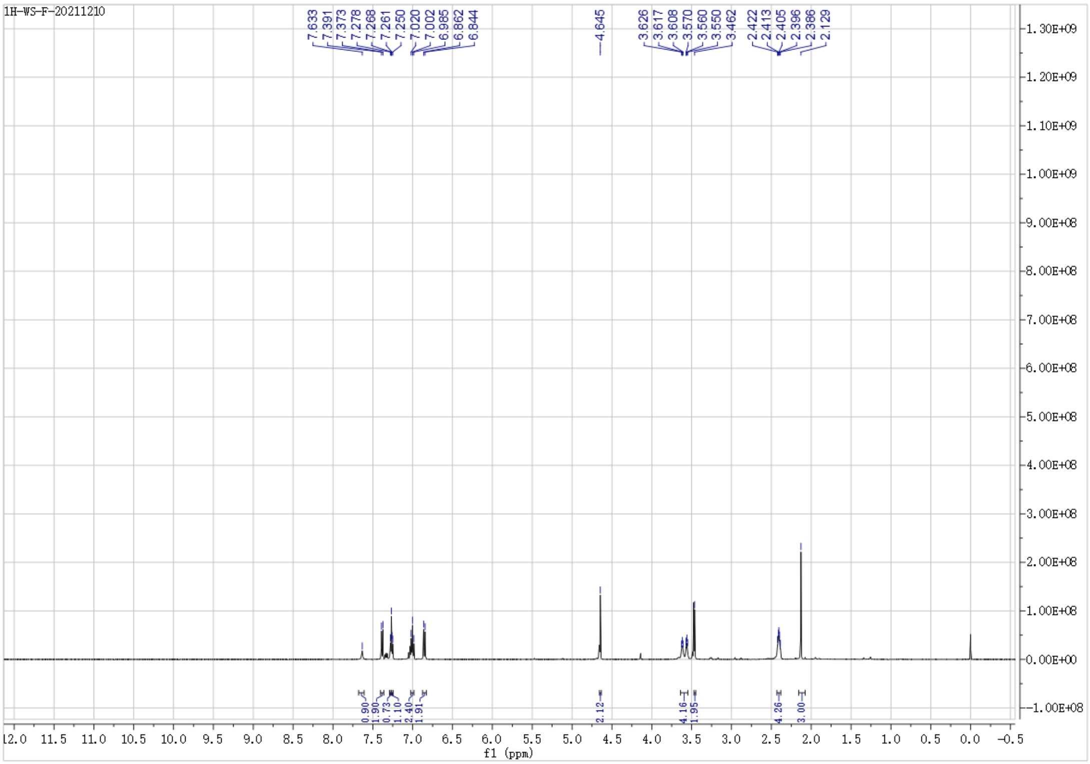

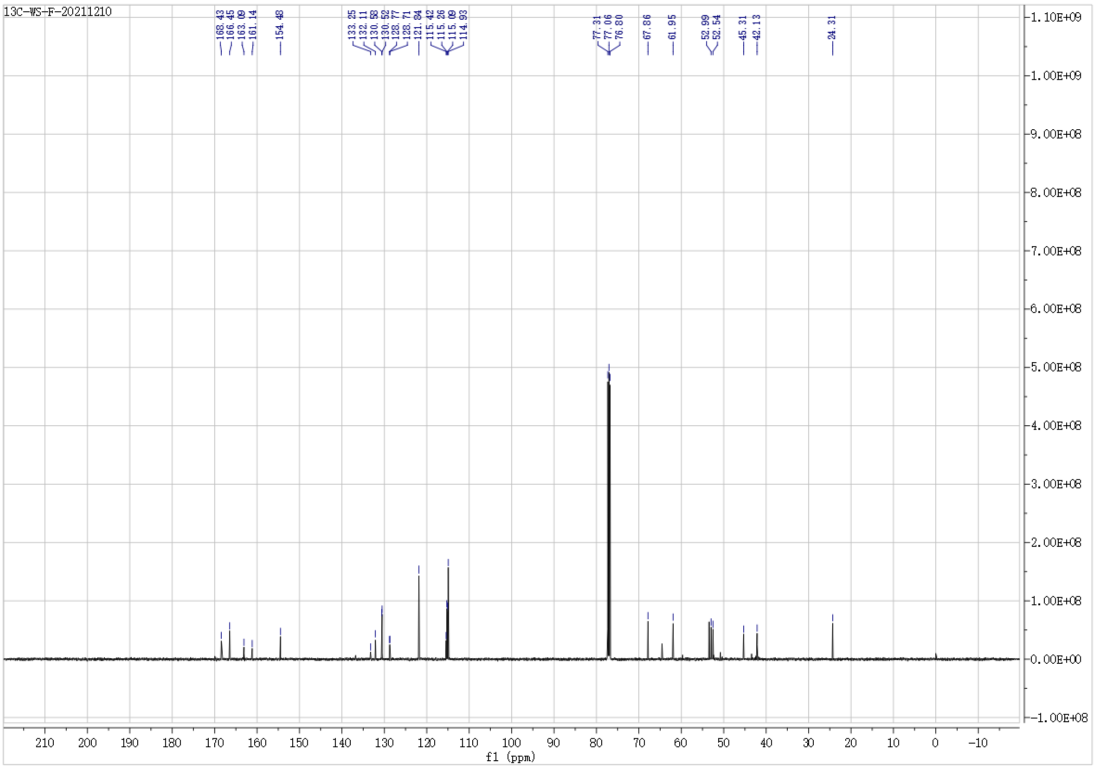

N-(4-(2-(4-(4-chlorobenzyl)piperazin-1-yl)-2-oxoethoxy)phenyl)acetamide (14c)

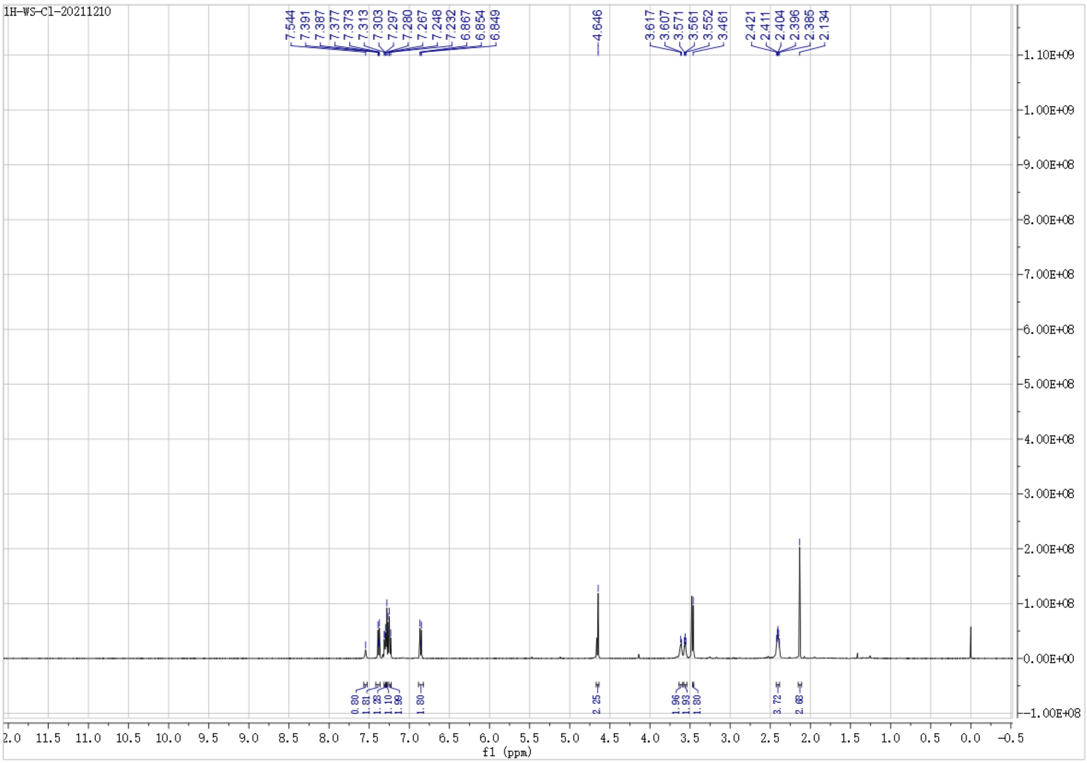

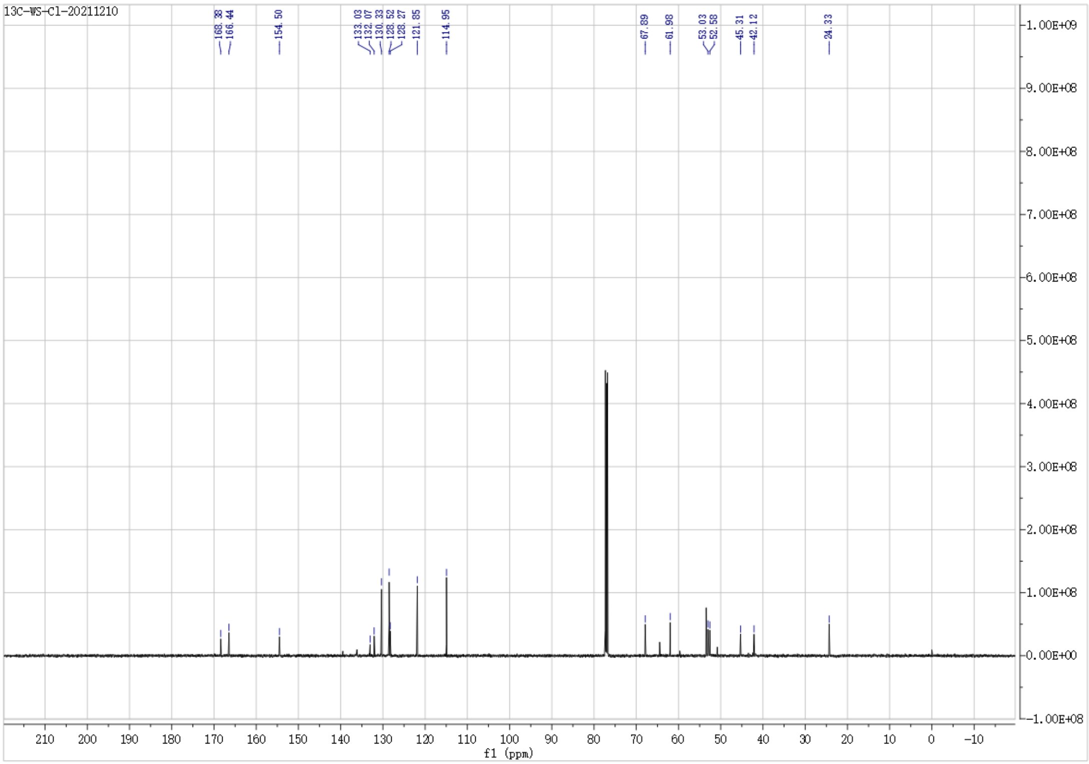

N-(4-(2-oxo-2-(4-(4-(trifluoromethyl)benzyl)piperazin-1-yl)ethoxy)phenyl)acetamide (14d)

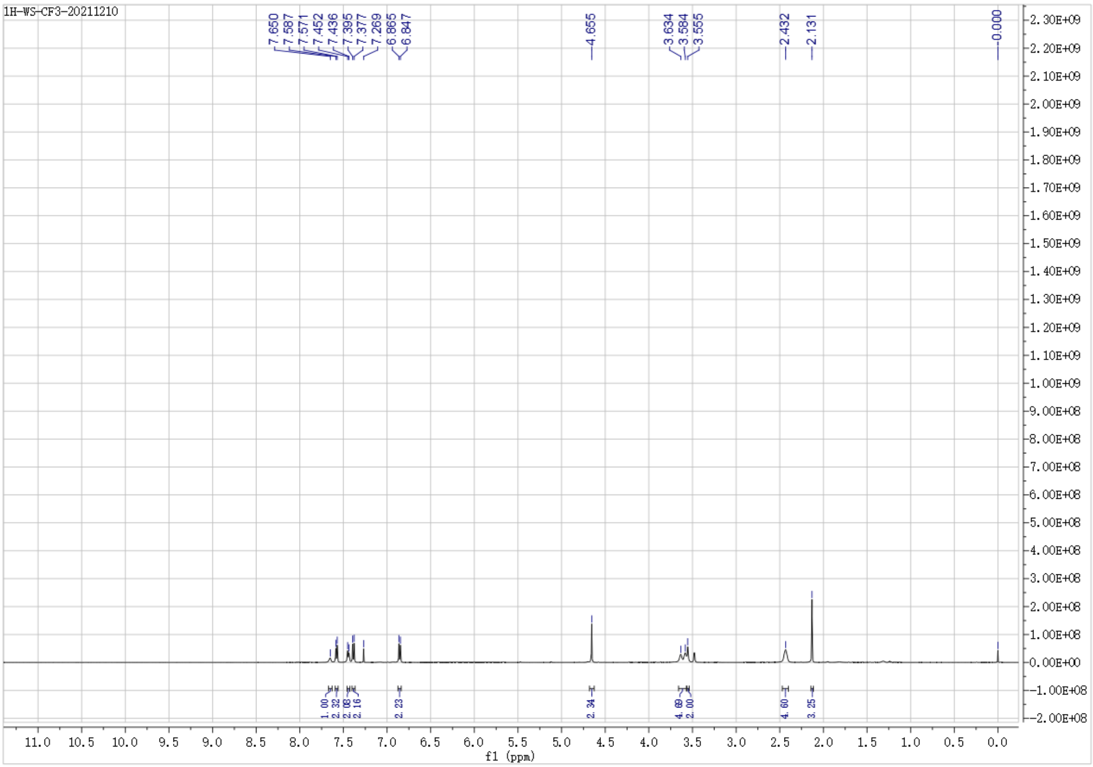

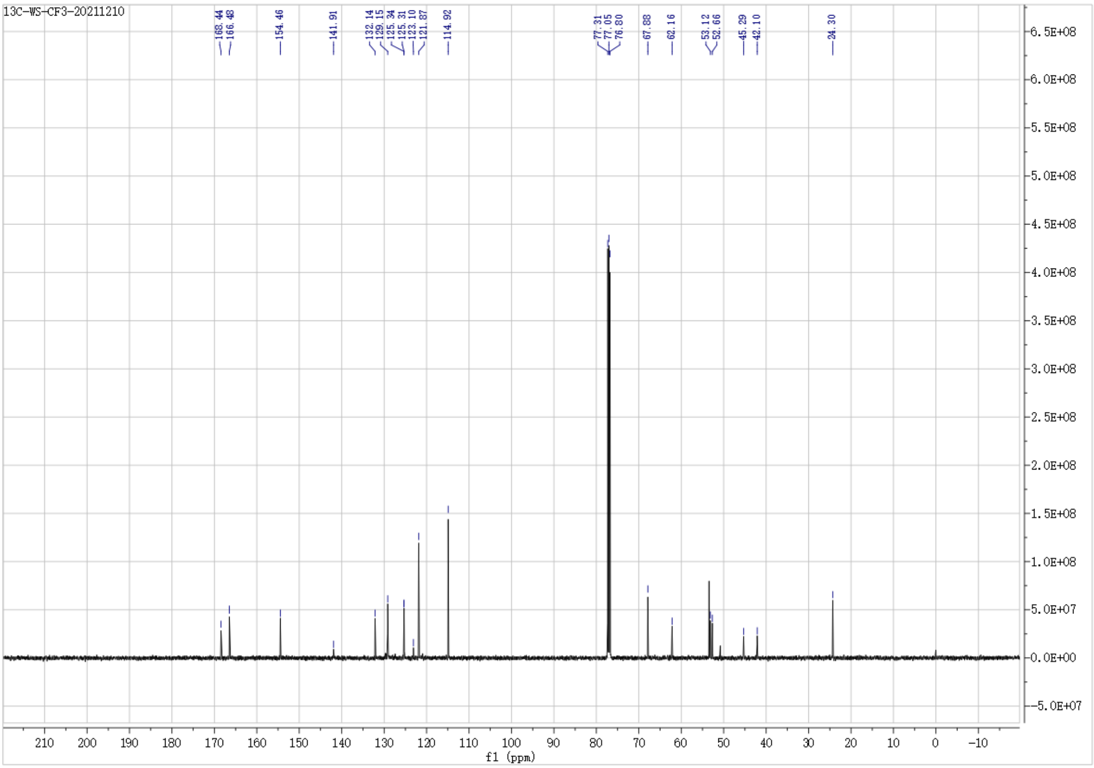

N-(4-(2-(4-(4-methylbenzyl)piperazin-1-yl)-2-oxoethoxy)phenyl)acetamide (14e)

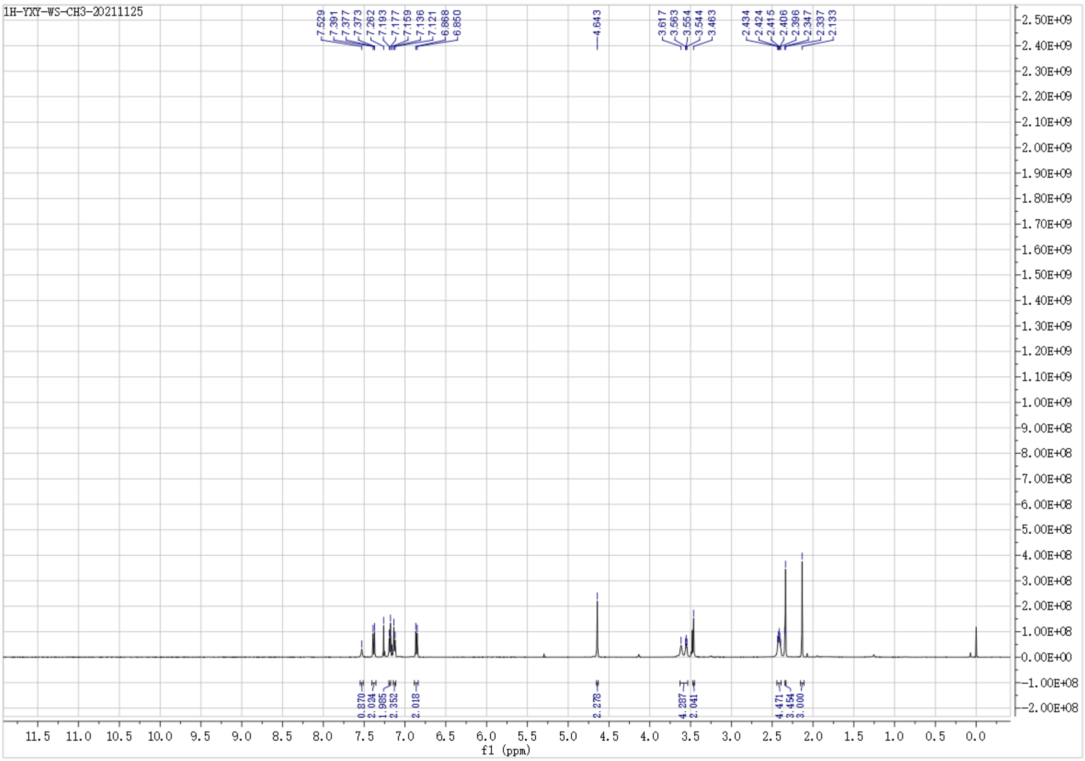

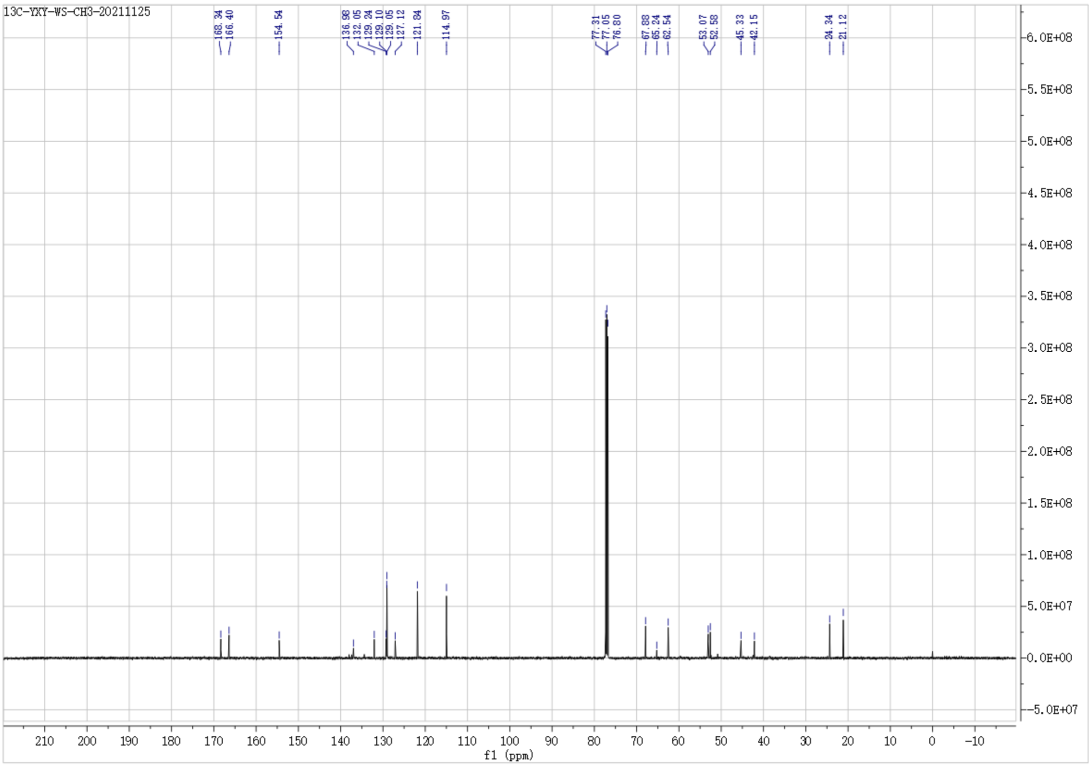

N-(4-(2-(4-butylpiperazin-1-yl)-2-oxoethoxy)phenyl)acetamide (15a)

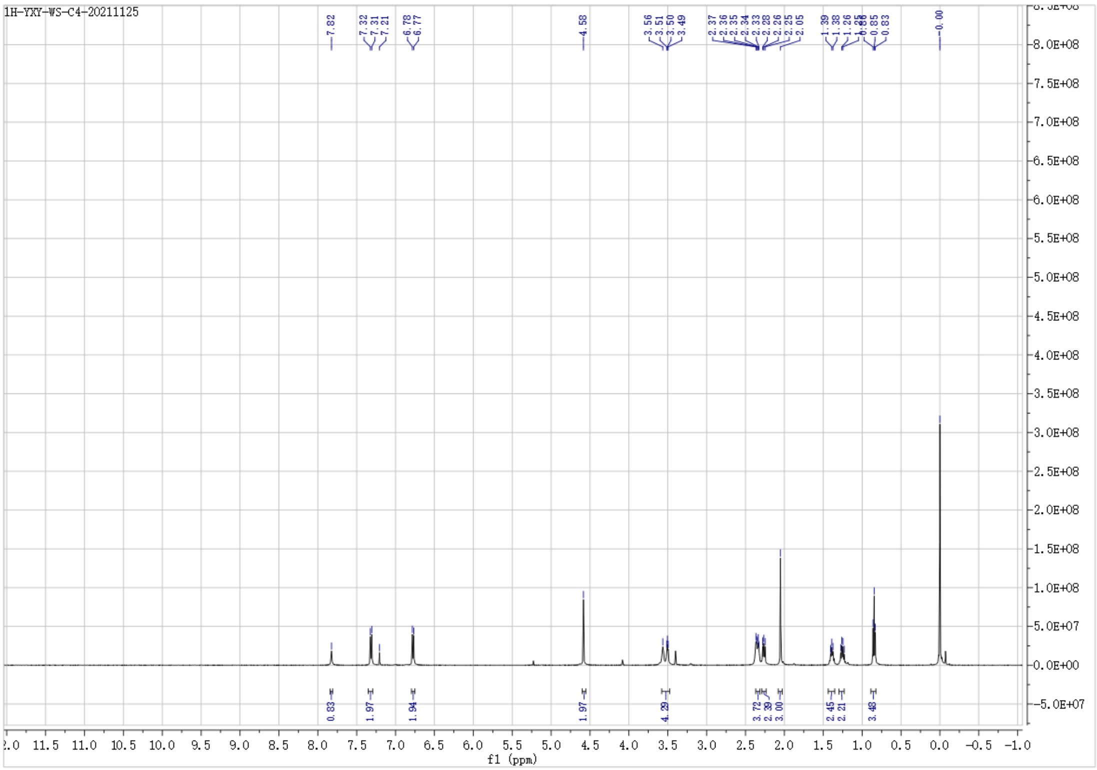

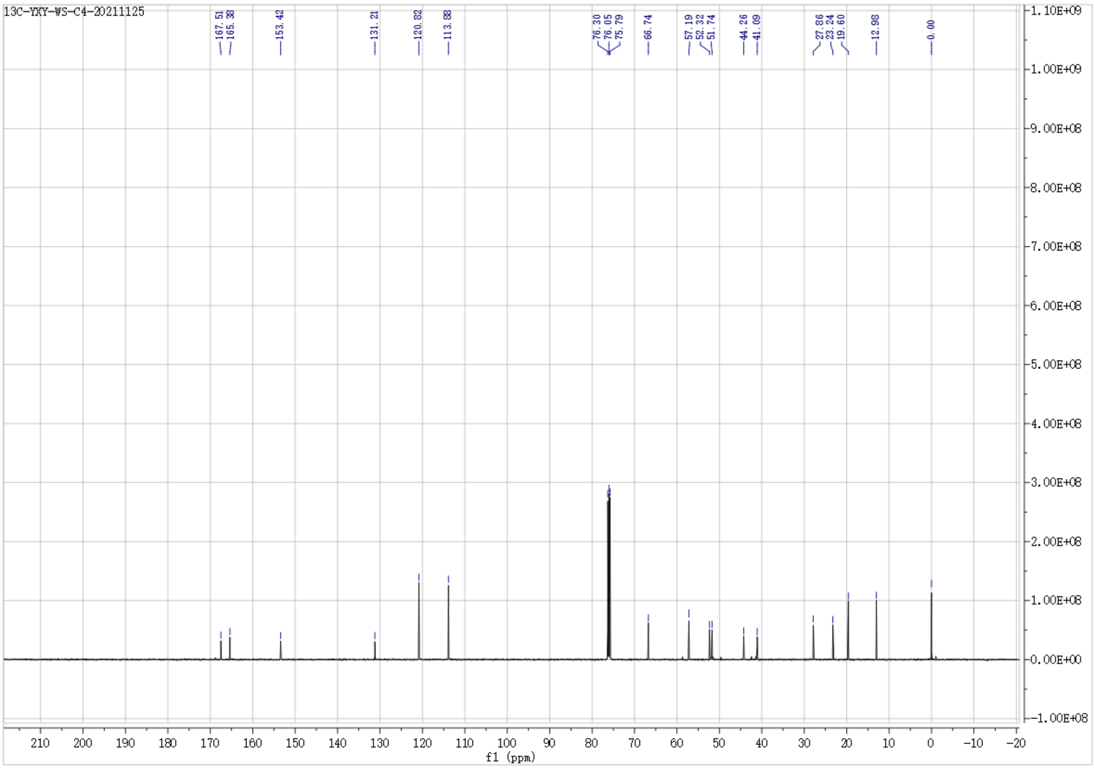

N-(4-(2-oxo-2-(4-pentylpiperazin-1-yl)ethoxy)phenyl)acetamide (15b)

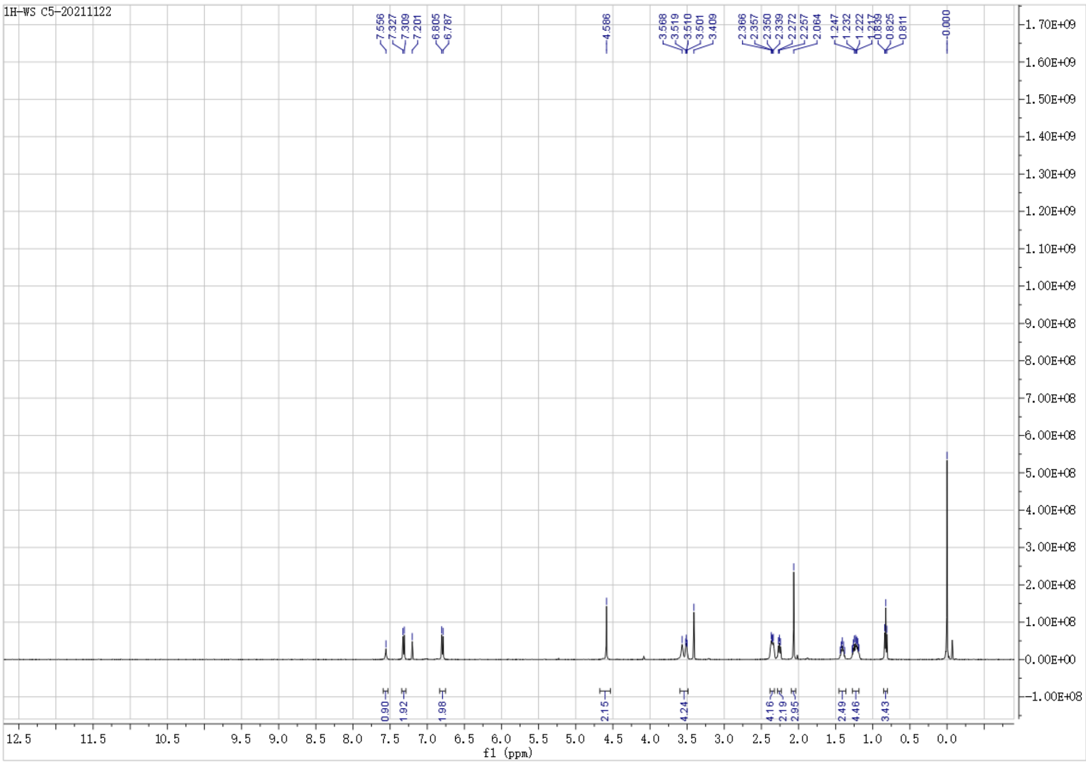

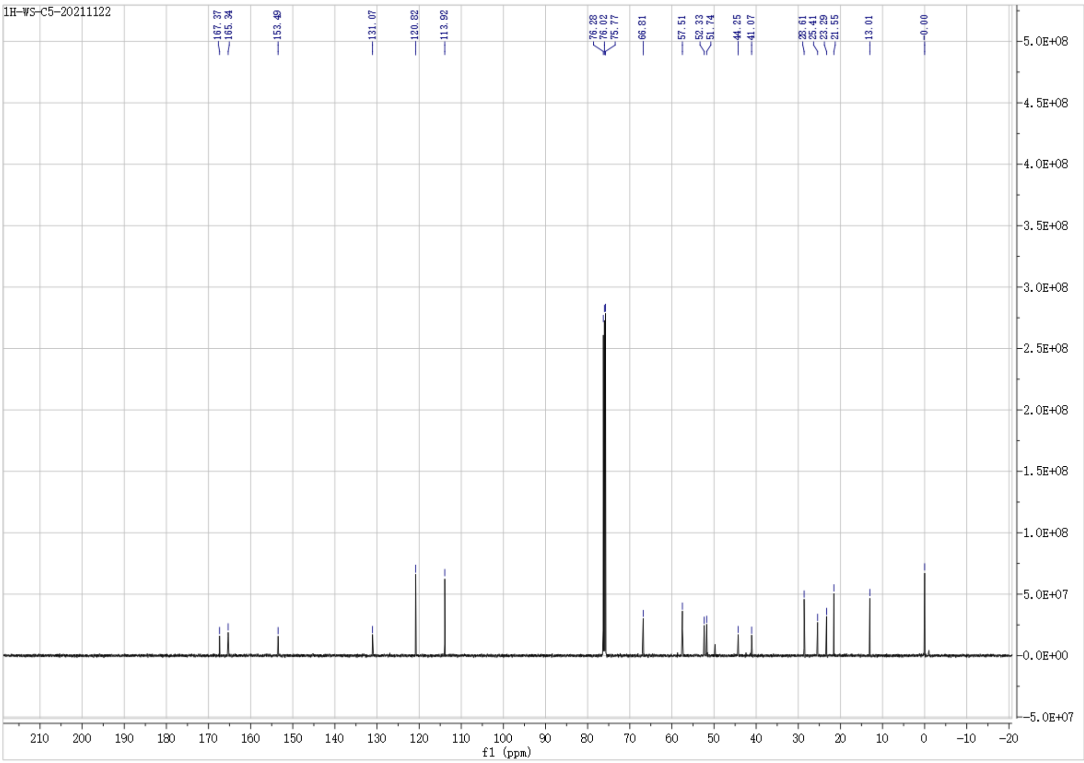

N-(4-(2-(4-hexylpiperazin-1-yl)-2-oxoethoxy)phenyl)acetamide (15c)

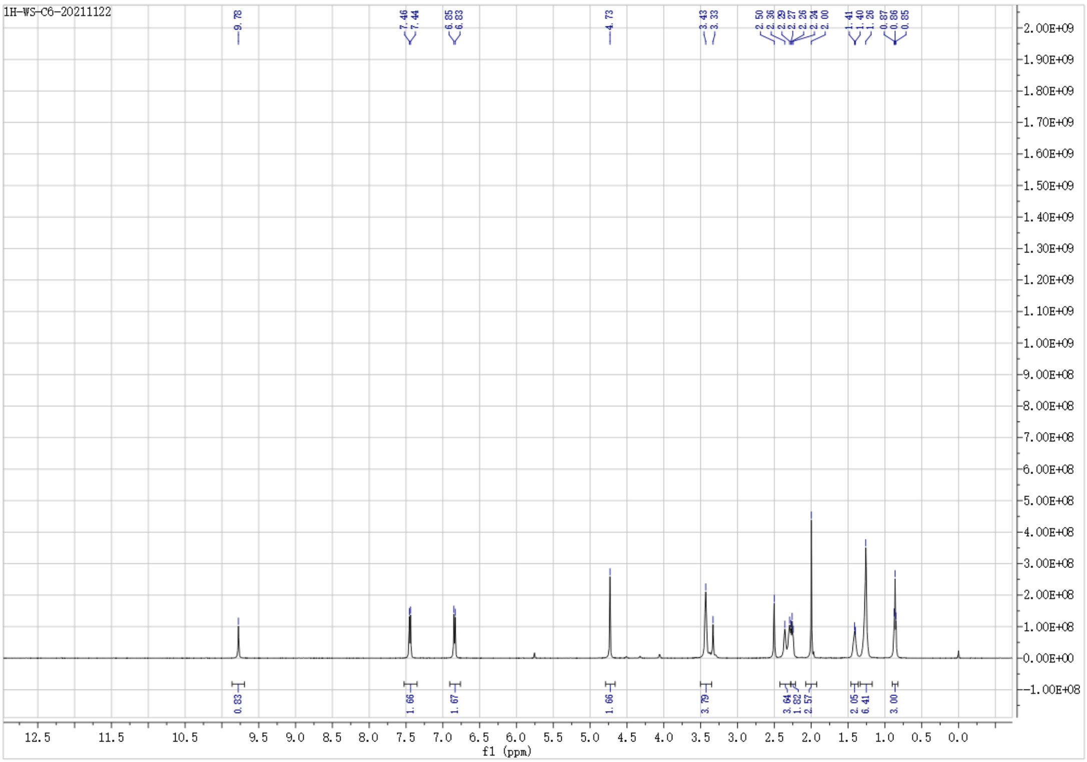

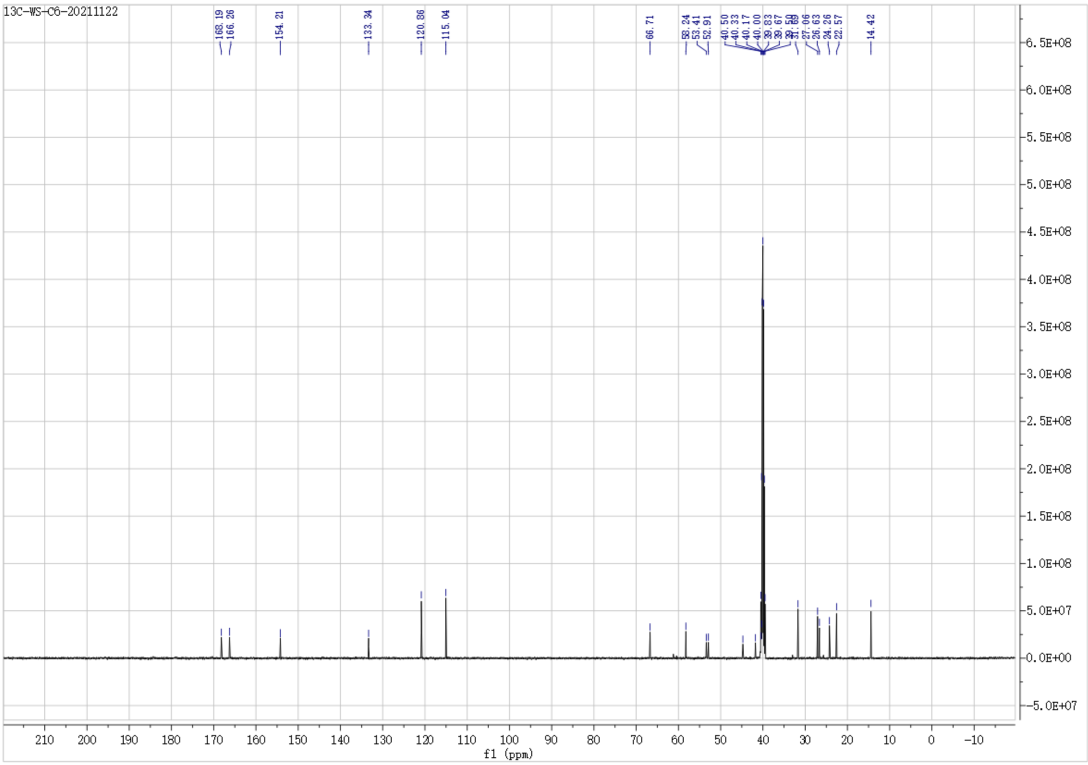

N-(4-(2-(4-heptylpiperazin-1-yl)-2-oxoethoxy)phenyl)acetamide (15d)

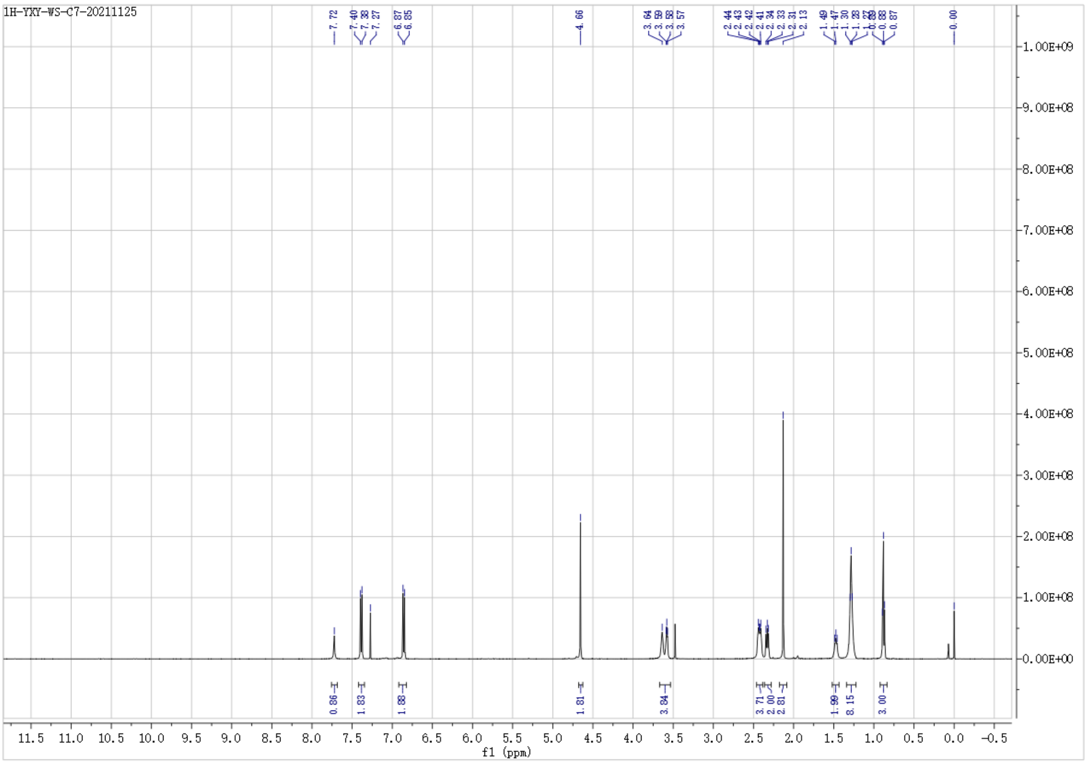

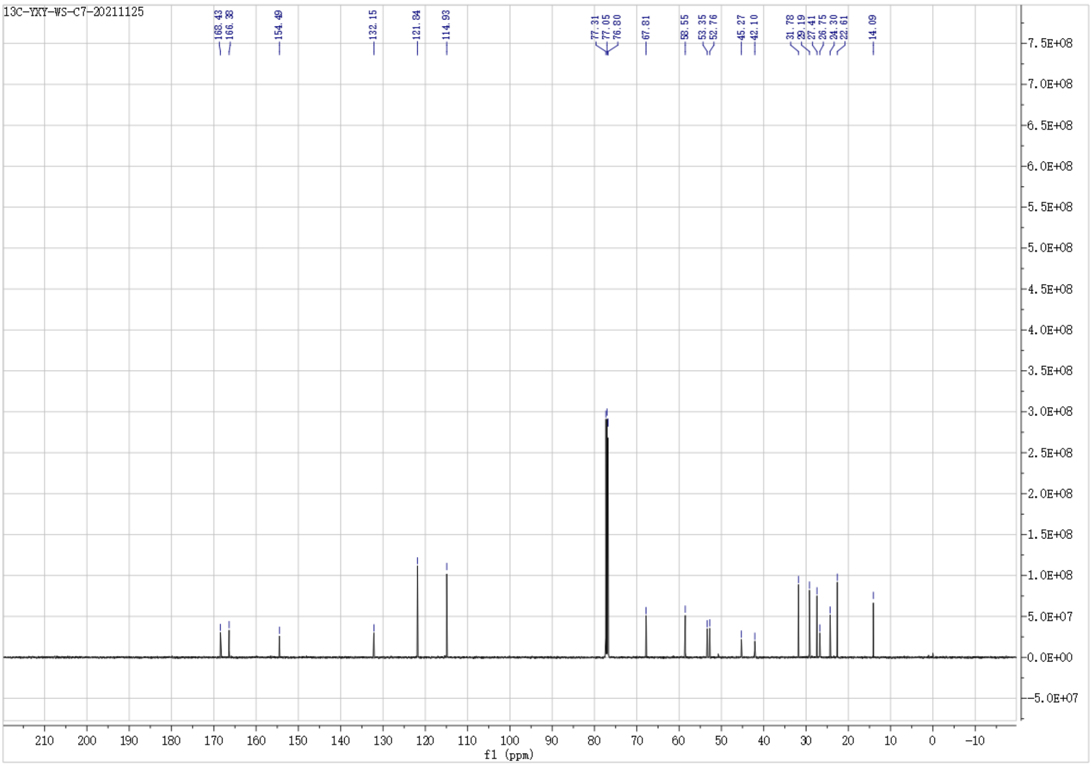

N-(4-(2-(4-octylpiperazin-1-yl)-2-oxoethoxy)phenyl)acetamide (15e)

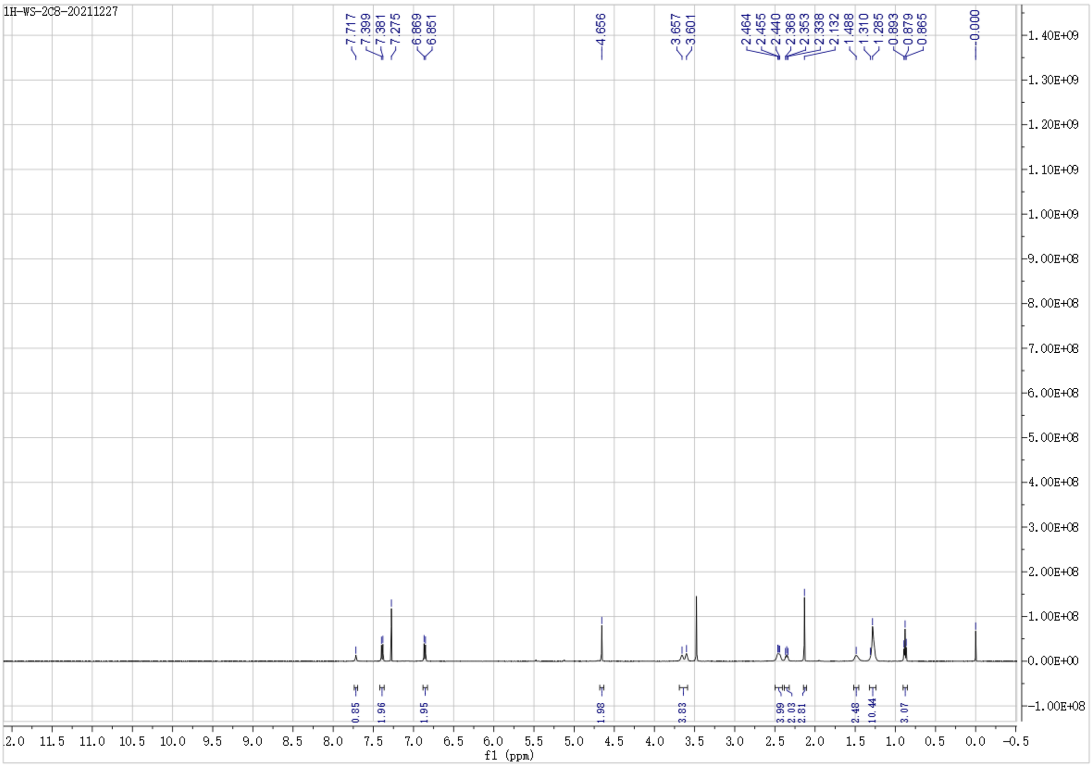

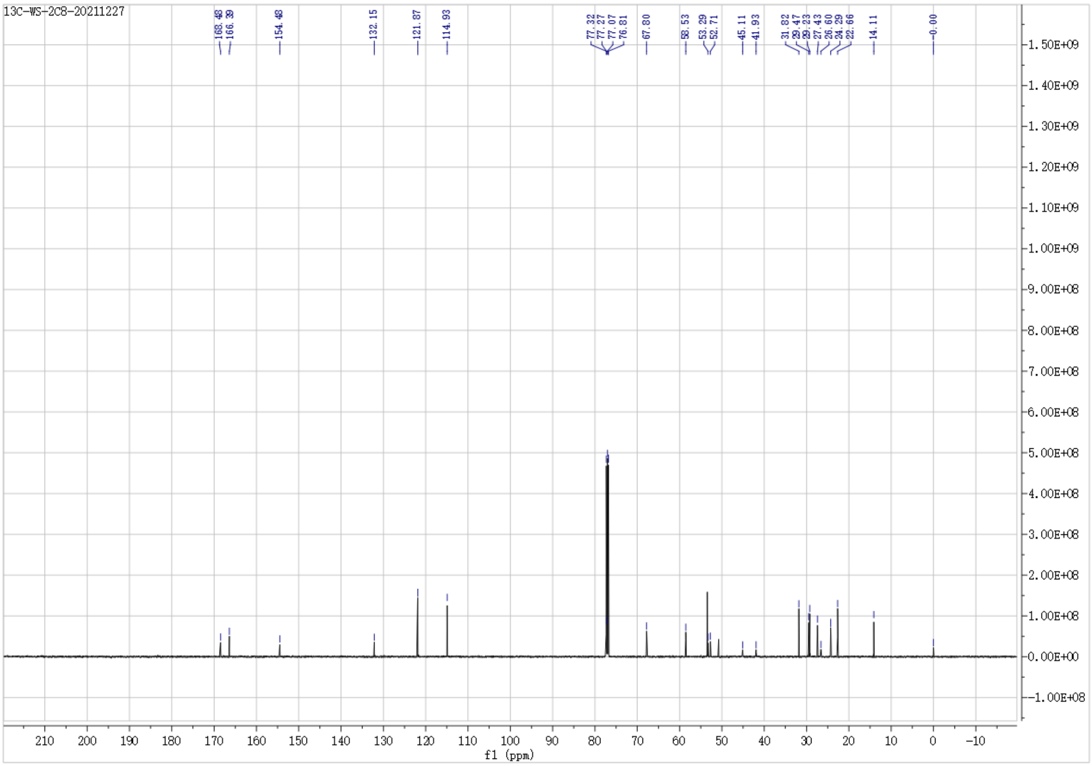

N-(4-(2-(4-nonylpiperazin-1-yl)-2-oxoethoxy)phenyl)acetamide (15f)

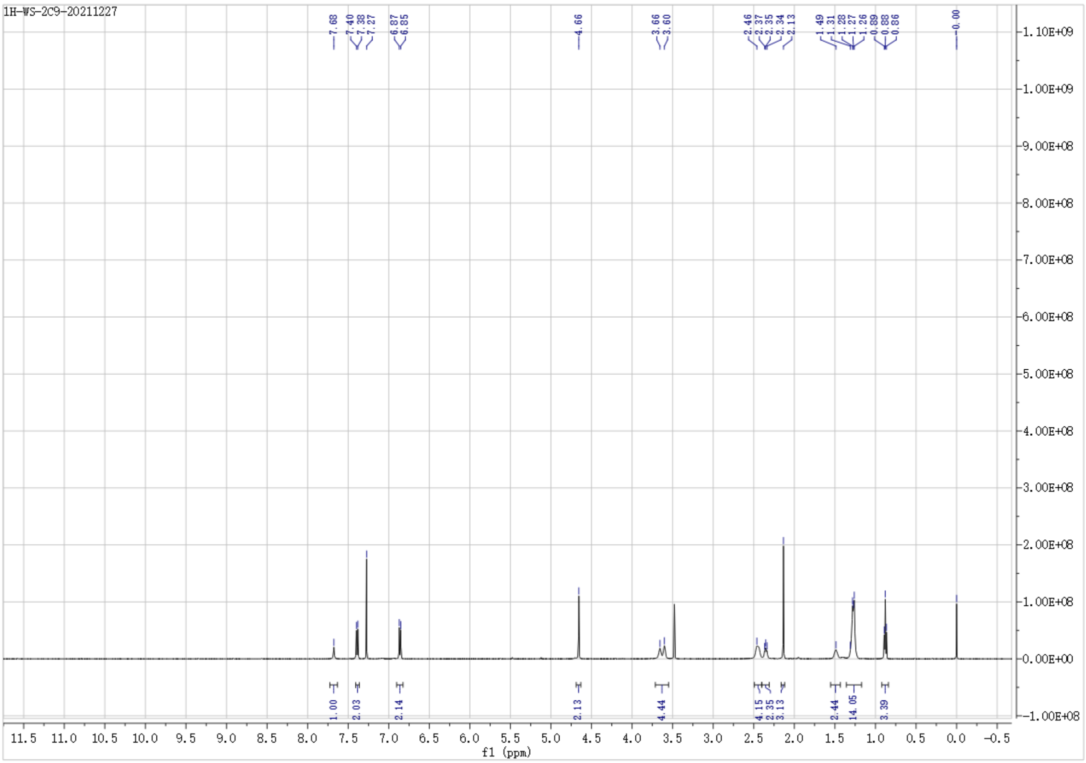

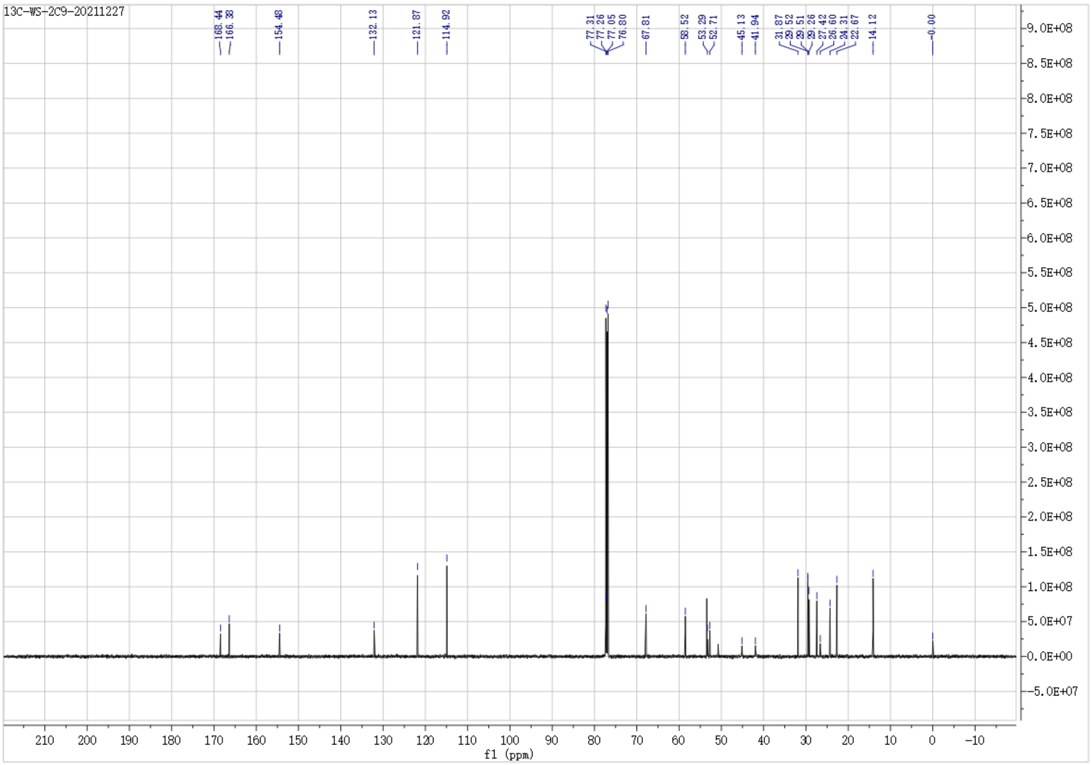

N-(3-(2-(4-benzylpiperazin-1-yl)-2-oxoethoxy)phenyl)acetamide (17a)

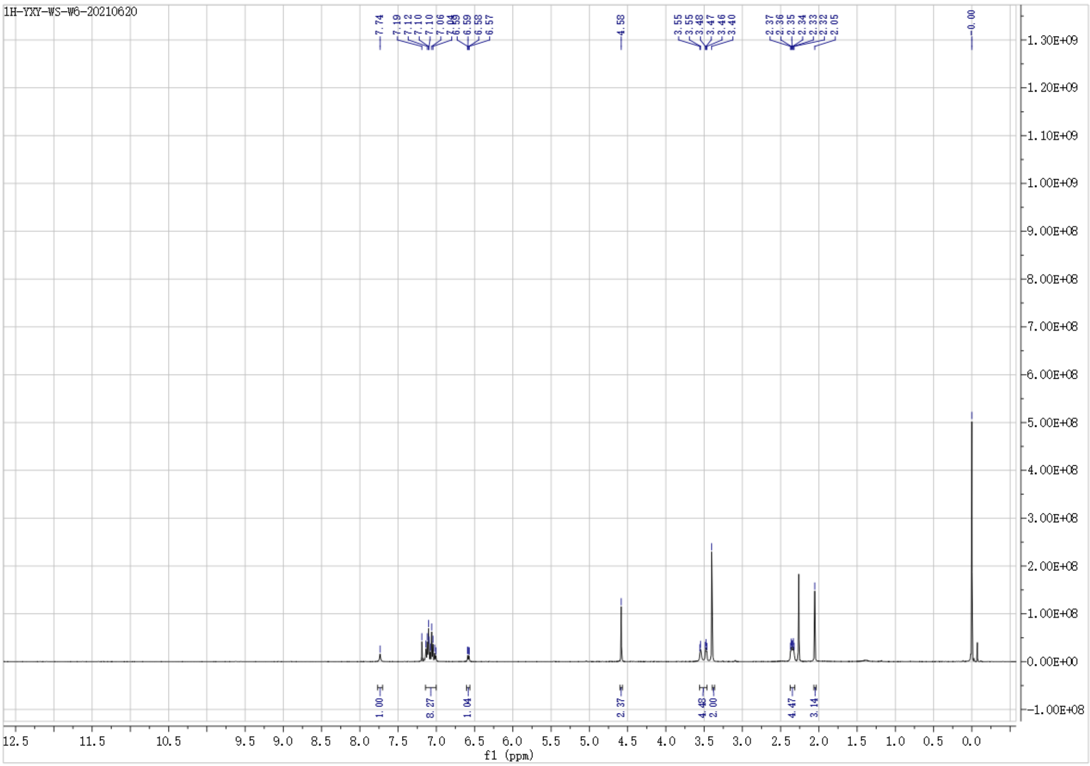

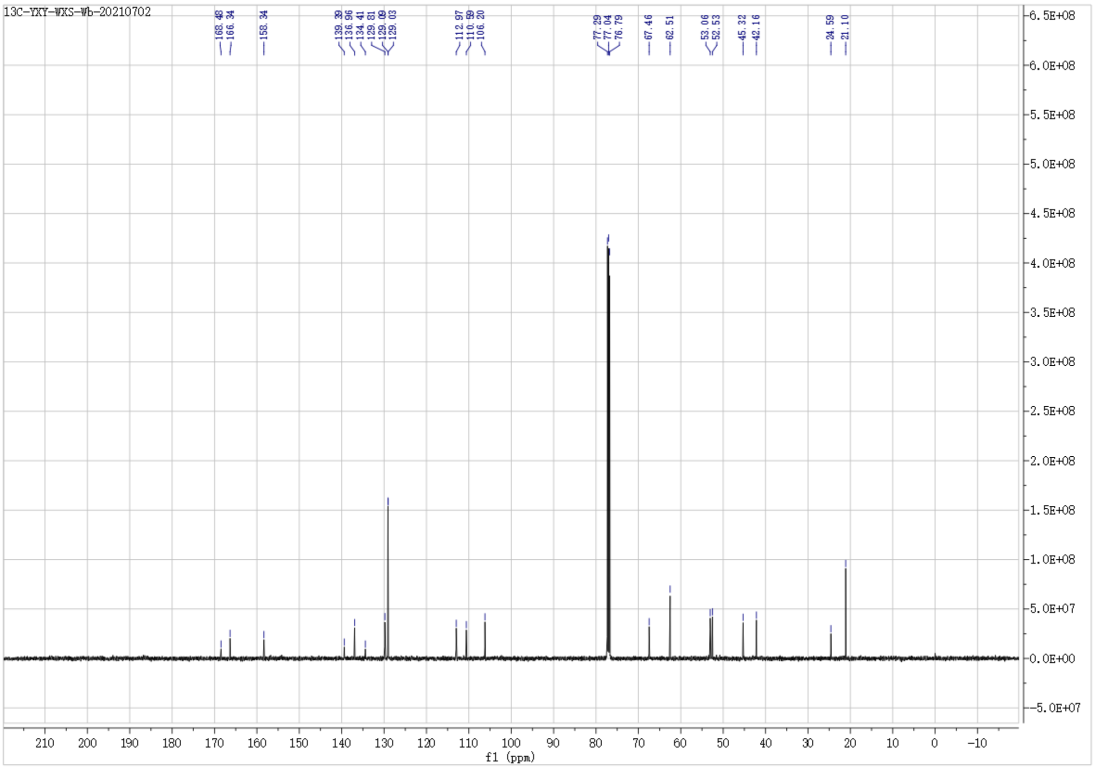

N-(3-(2-(4-(4-fluorobenzyl)piperazin-1-yl)-2-oxoethoxy)phenyl)acetamide (17b)

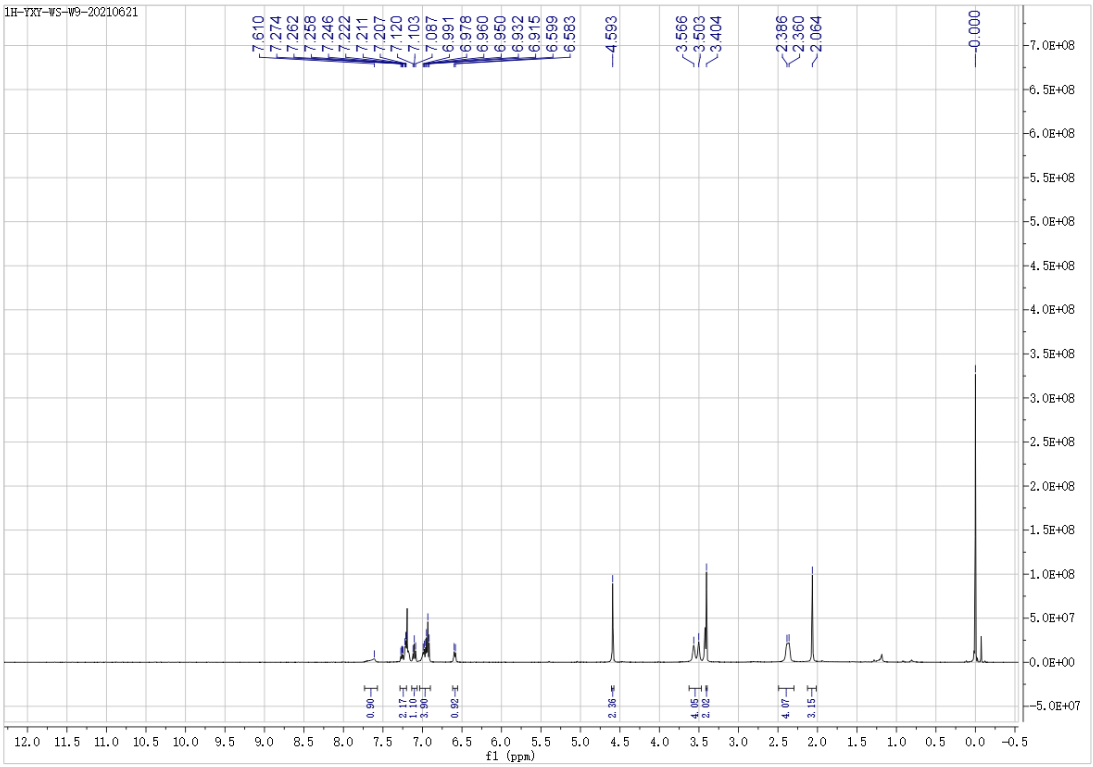

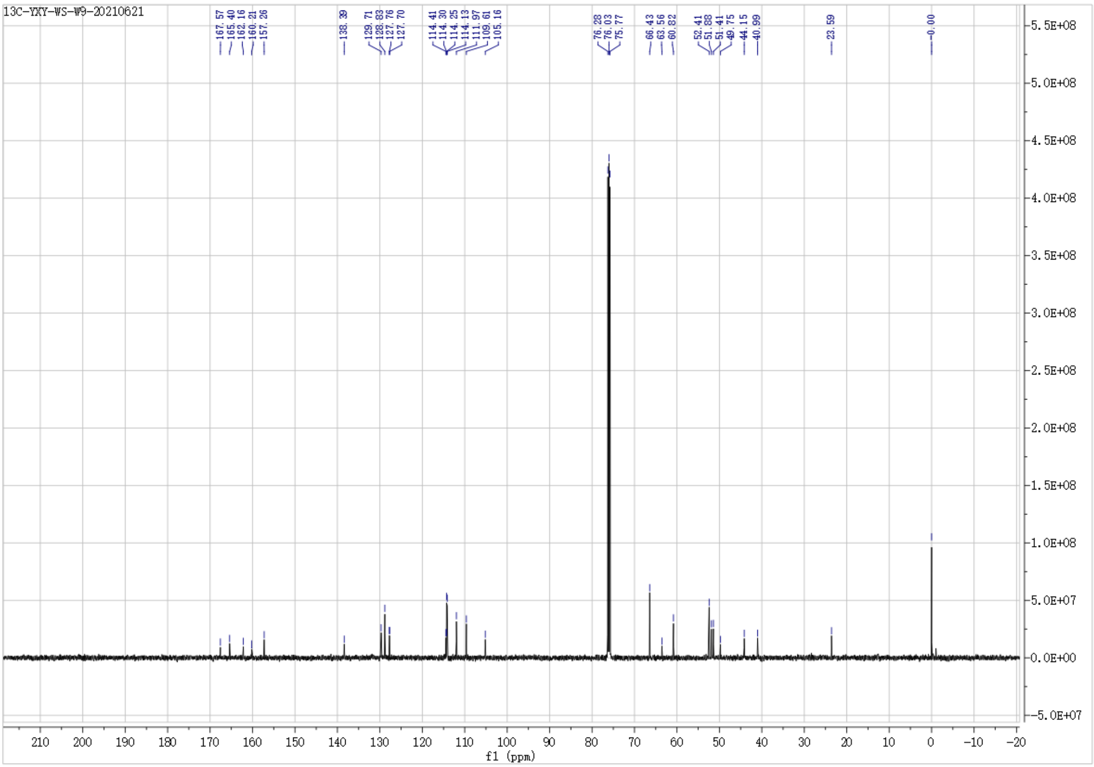

N-(3-(2-(4-(4-chlorobenzyl)piperazin-1-yl)-2-oxoethoxy)phenyl)acetamide (17c)

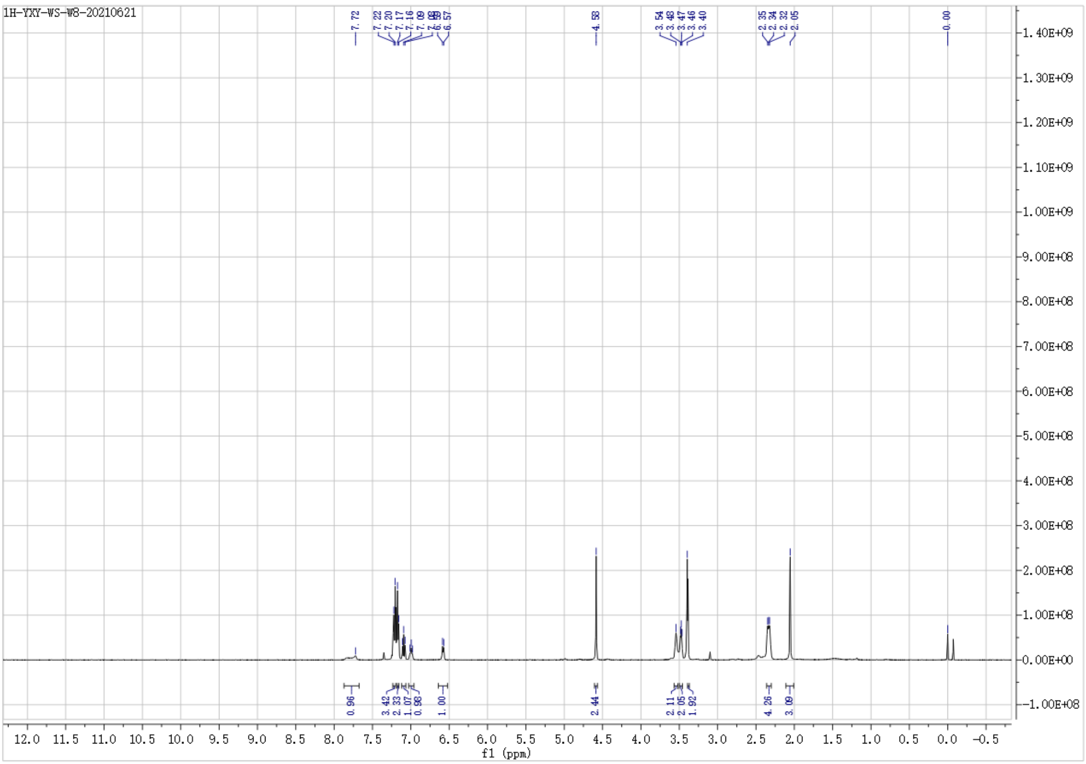

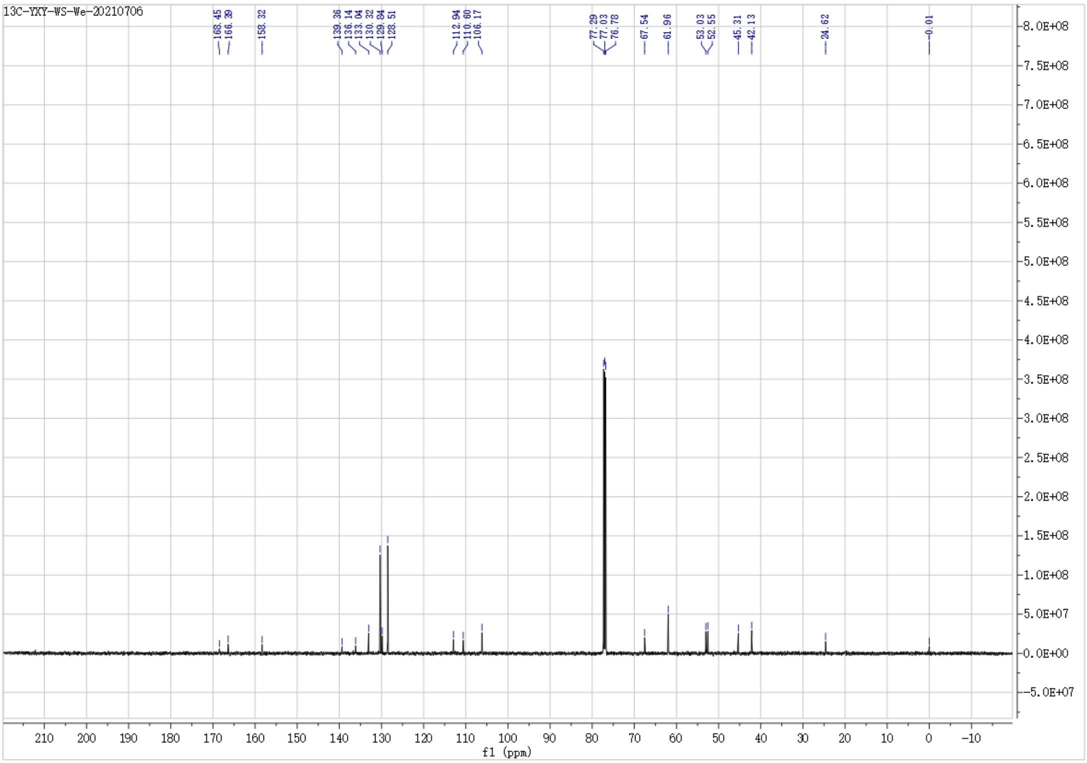

N-(3-(2-oxo-2-(4-(4-(trifluoromethyl)benzyl)piperazin-1-yl)ethoxy)phenyl)acetamide (17d)

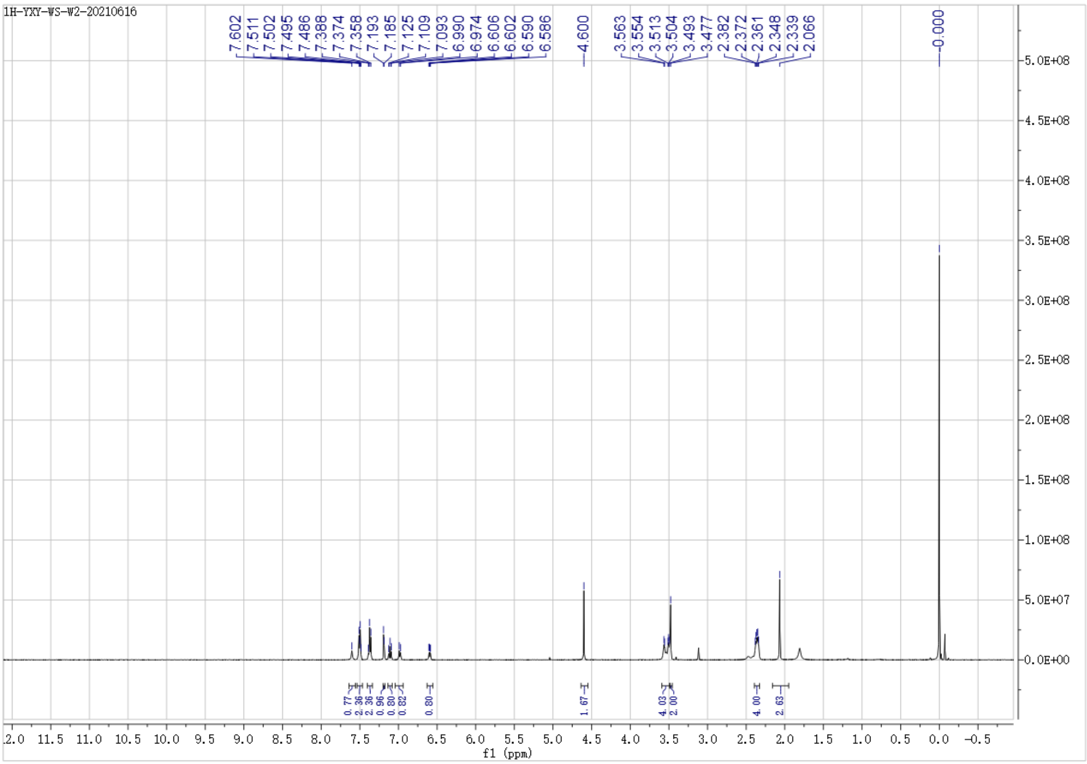

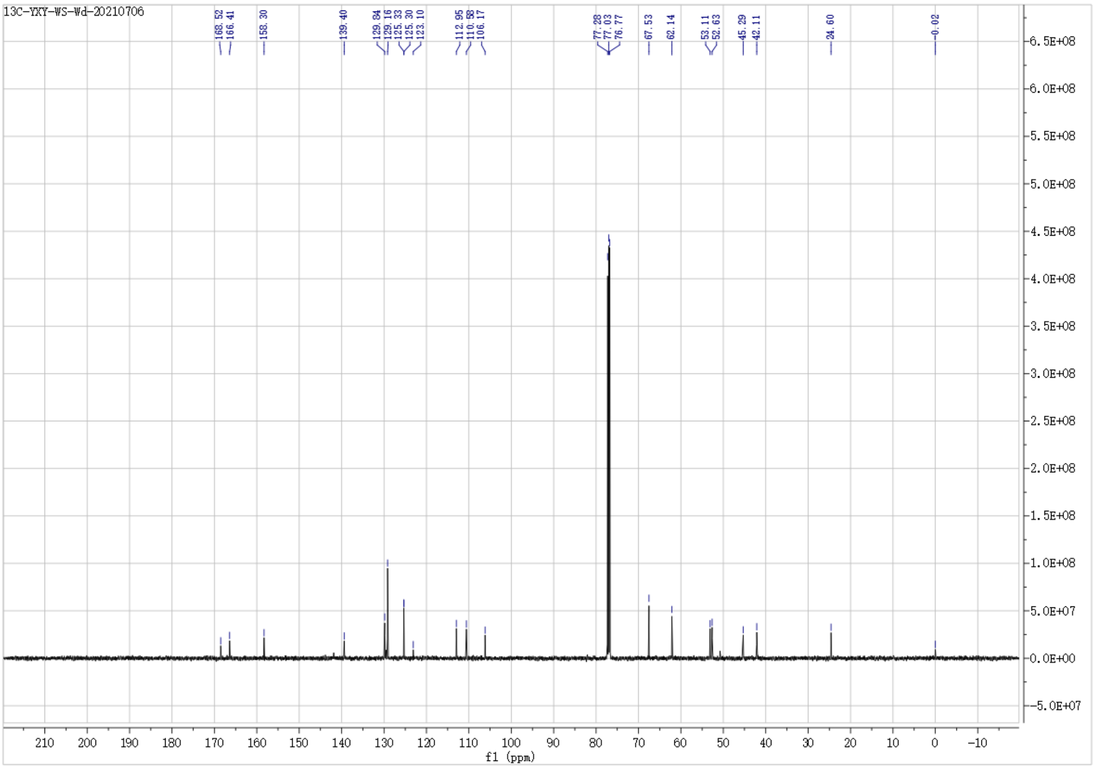

N-(3-(2-(4-(4-methylbenzyl)piperazin-1-yl)-2-oxoethoxy)phenyl)acetamide (17e)

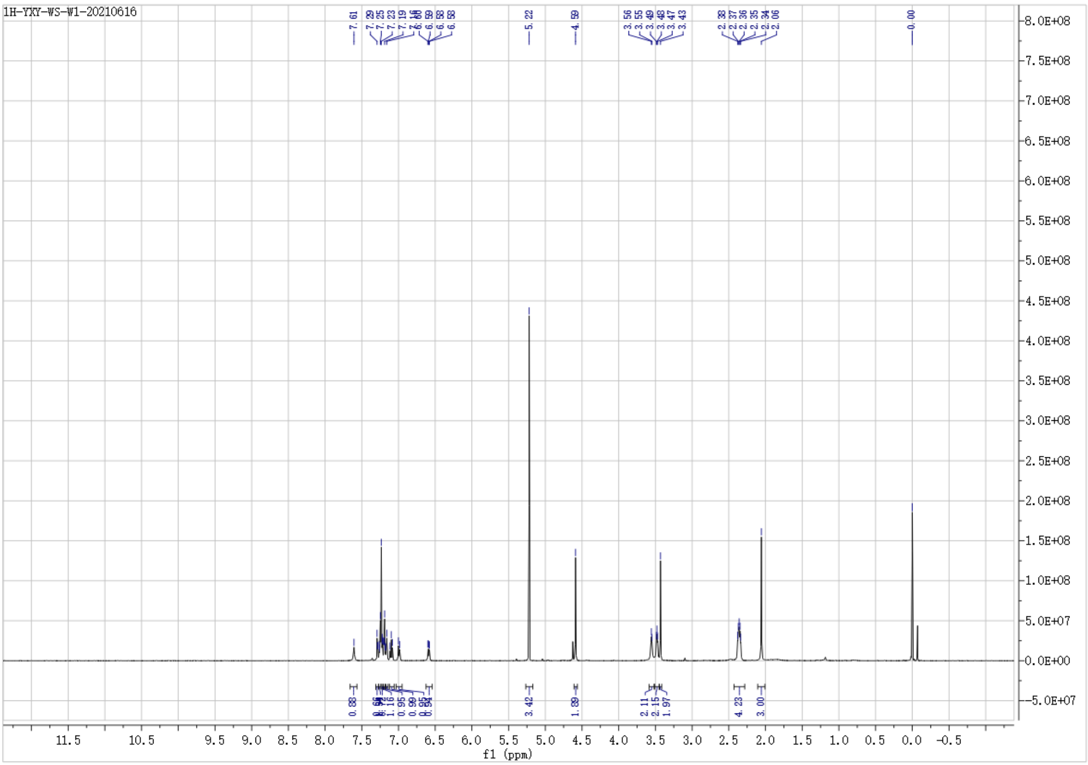

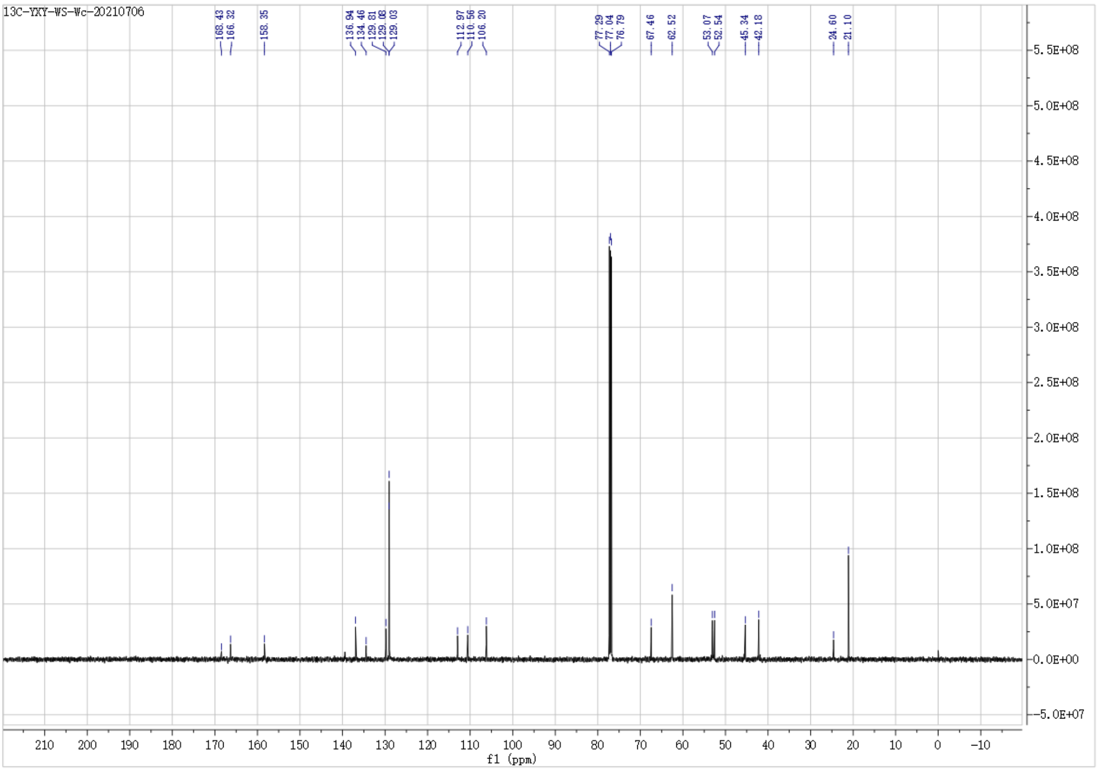

N-(3-(2-(4-butylpiperazin-1-yl)-2-oxoethoxy)phenyl)acetamide (18a)

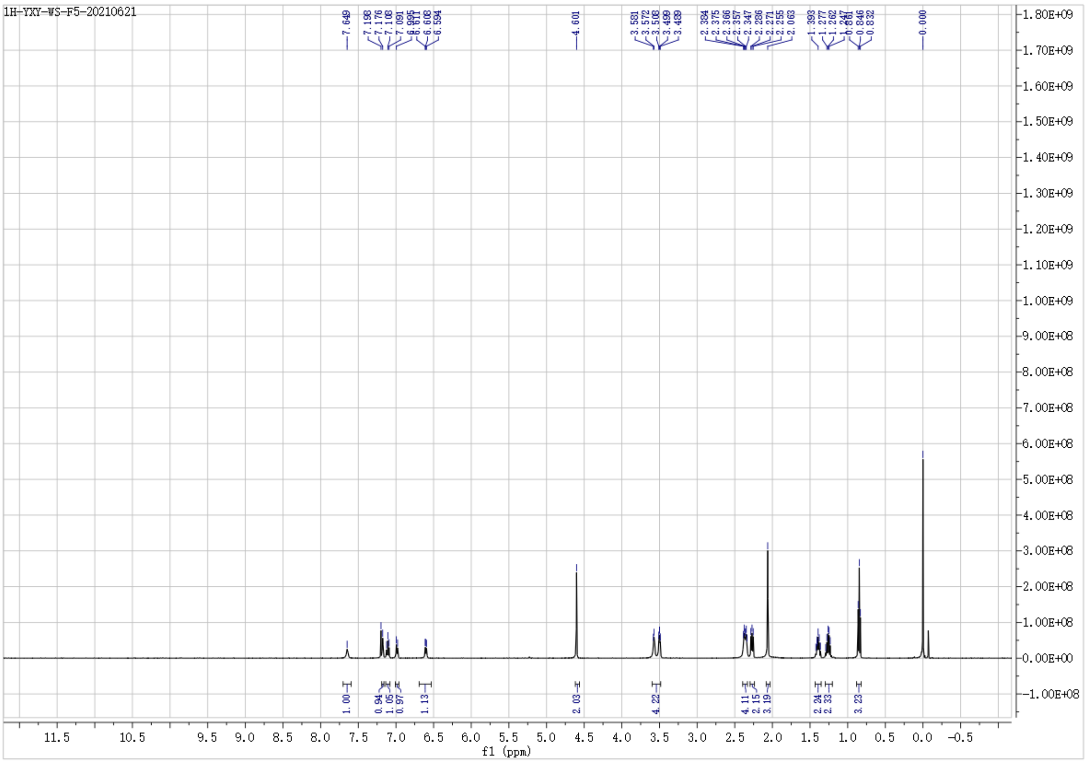

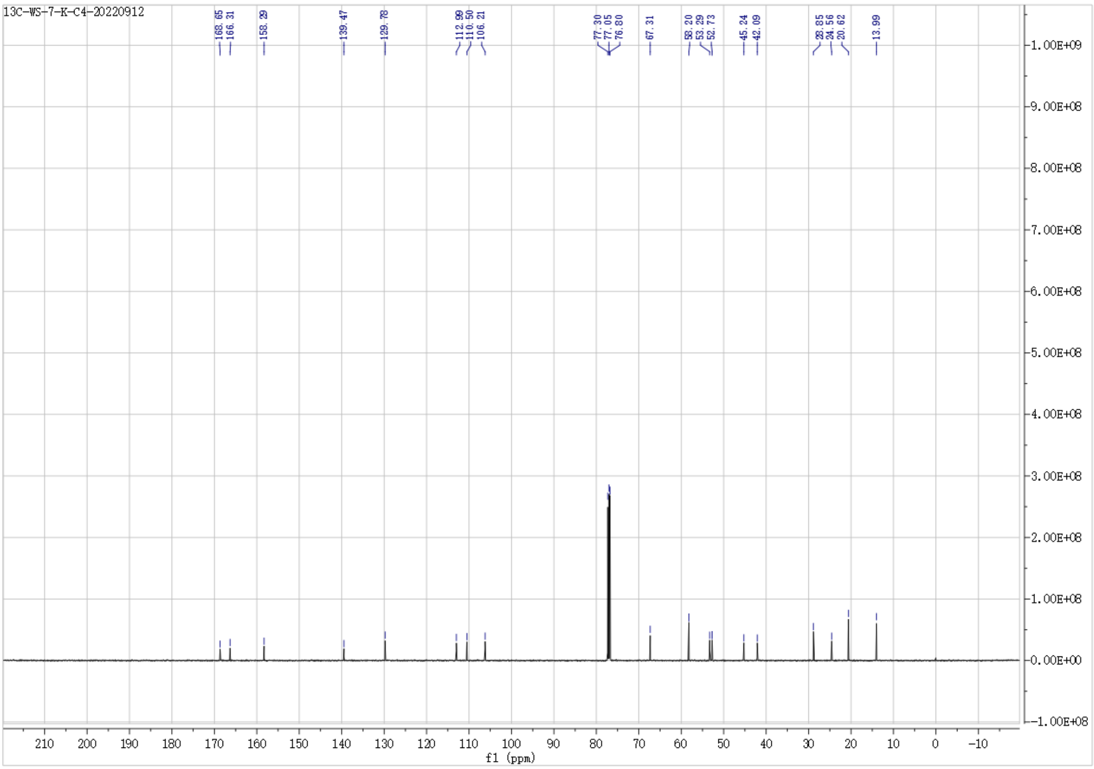

N-(3-(2-oxo-2-(4-pentylpiperazin-1-yl)ethoxy)phenyl)acetamide (18b)

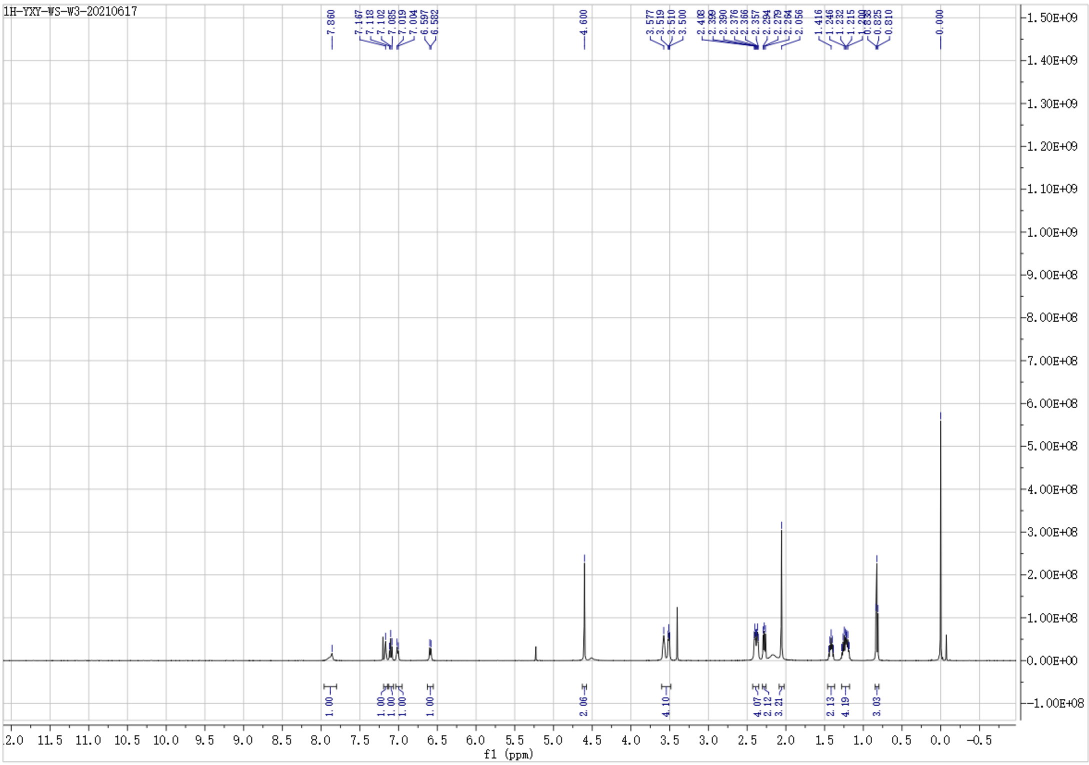

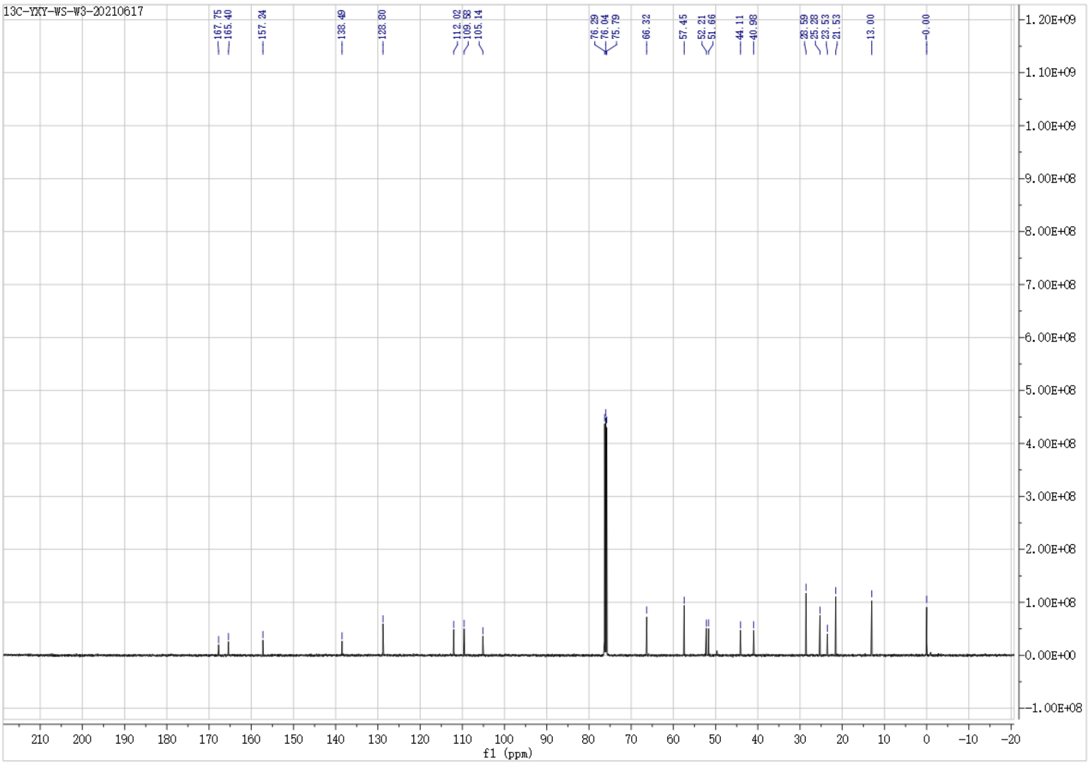

N-(3-(2-(4-hexylpiperazin-1-yl)-2-oxoethoxy)phenyl)acetamide (18c)

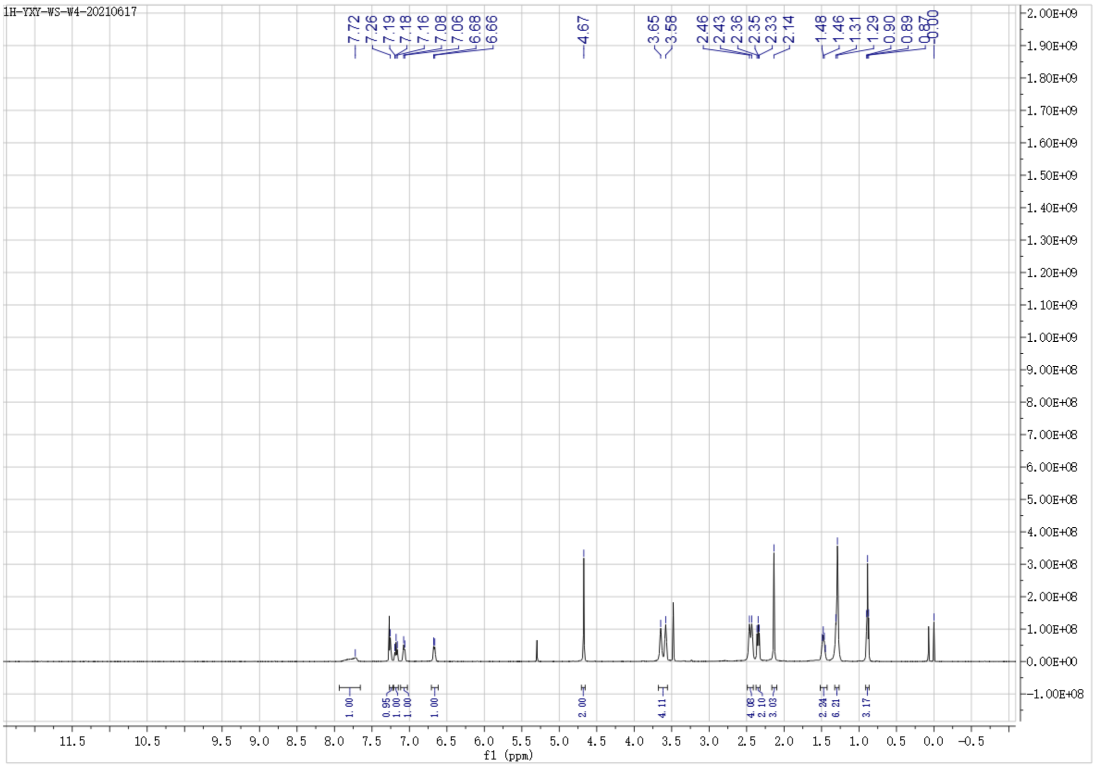

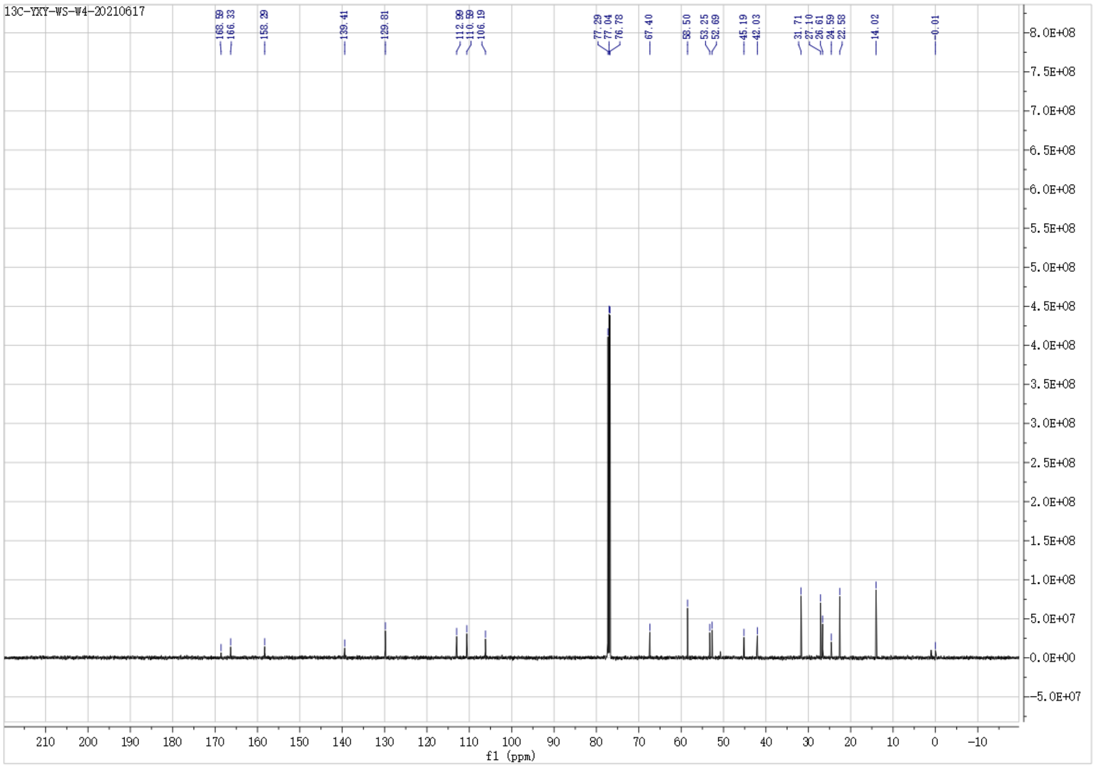

N-(3-(2-(4-heptylpiperazin-1-yl)-2-oxoethoxy)phenyl)acetamide (18d)

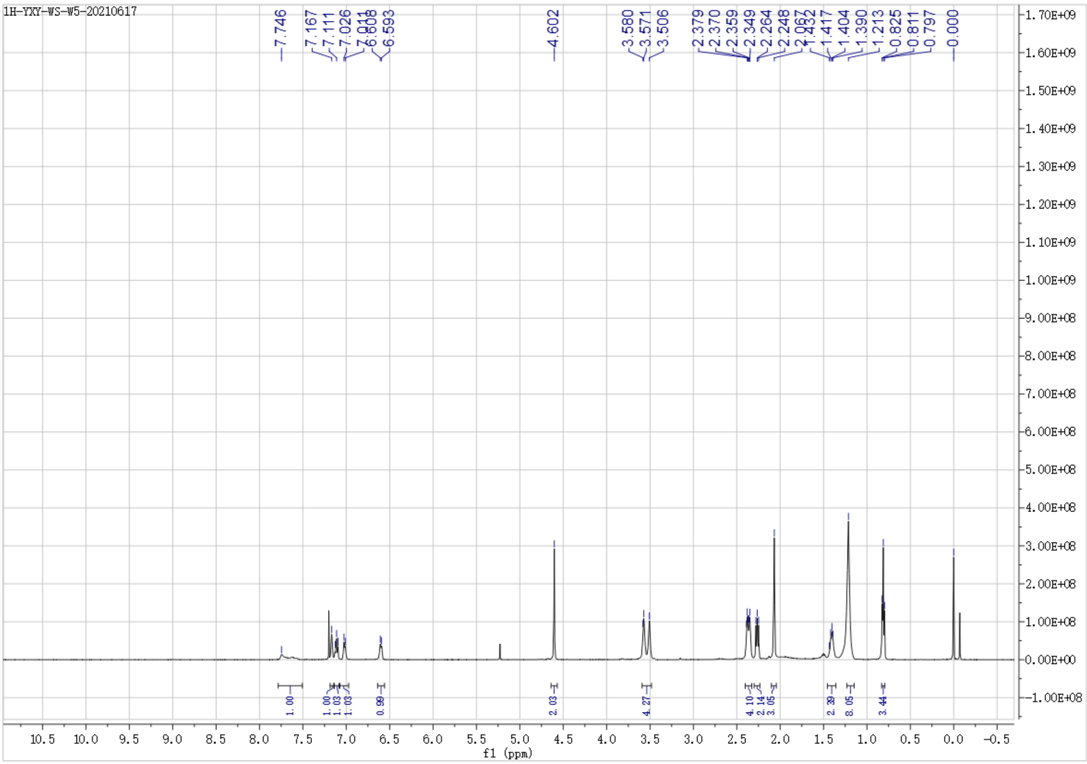

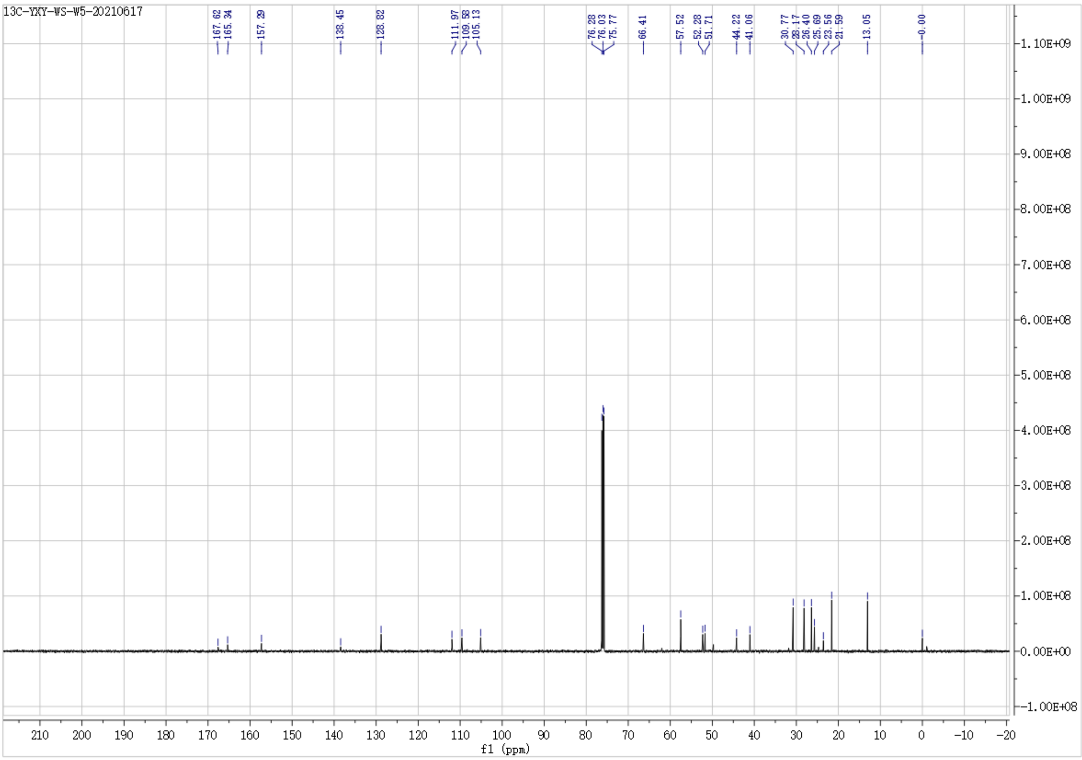

N-(3-(2-(4-octylpiperazin-1-yl)-2-oxoethoxy)phenyl)acetamide (18e)

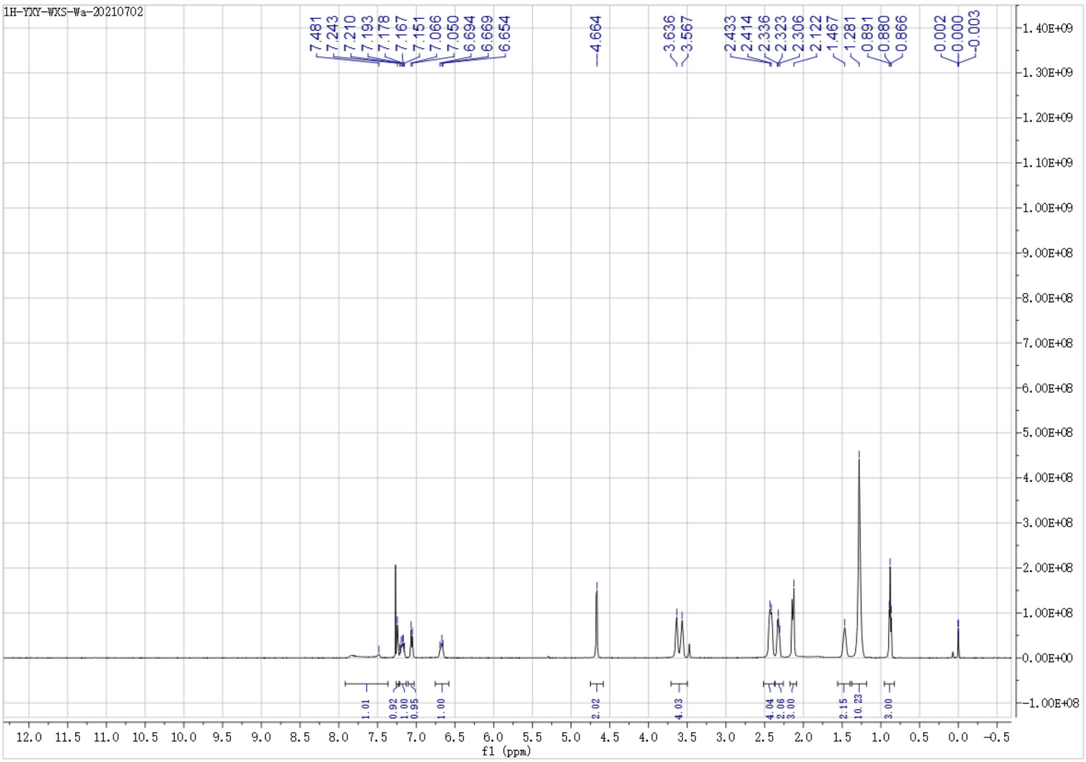

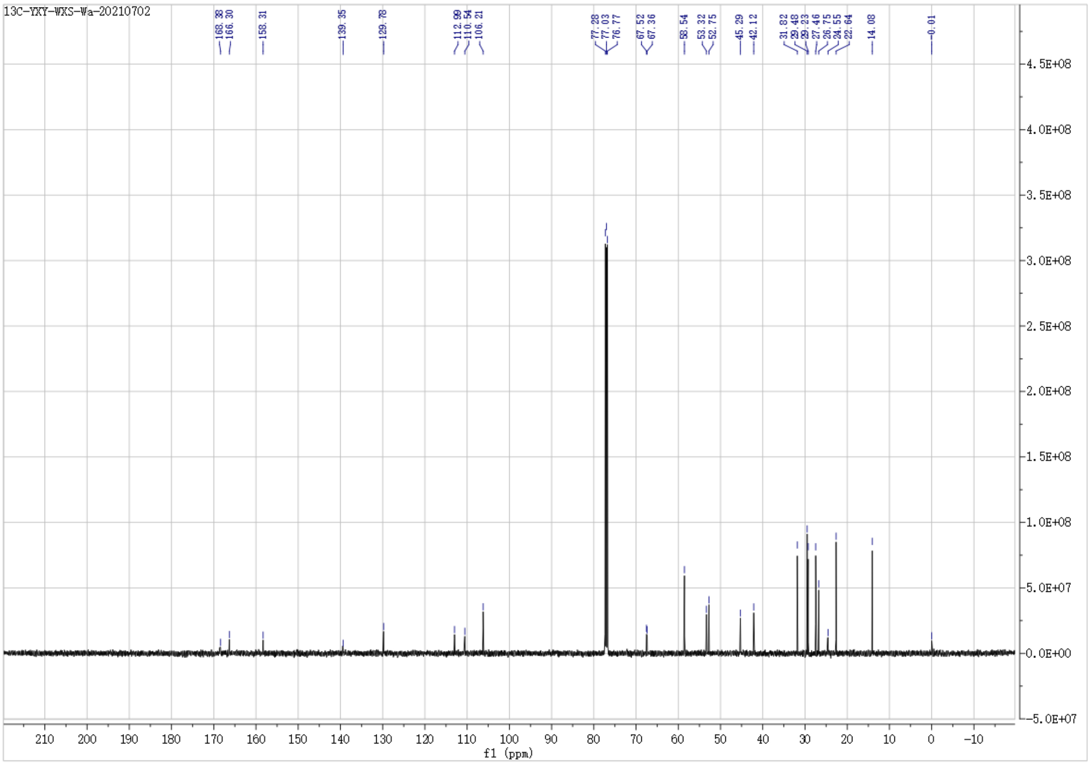

N-(3-(2-(4-nonylpiperazin-1-yl)-2-oxoethoxy)phenyl)acetamide (18f)

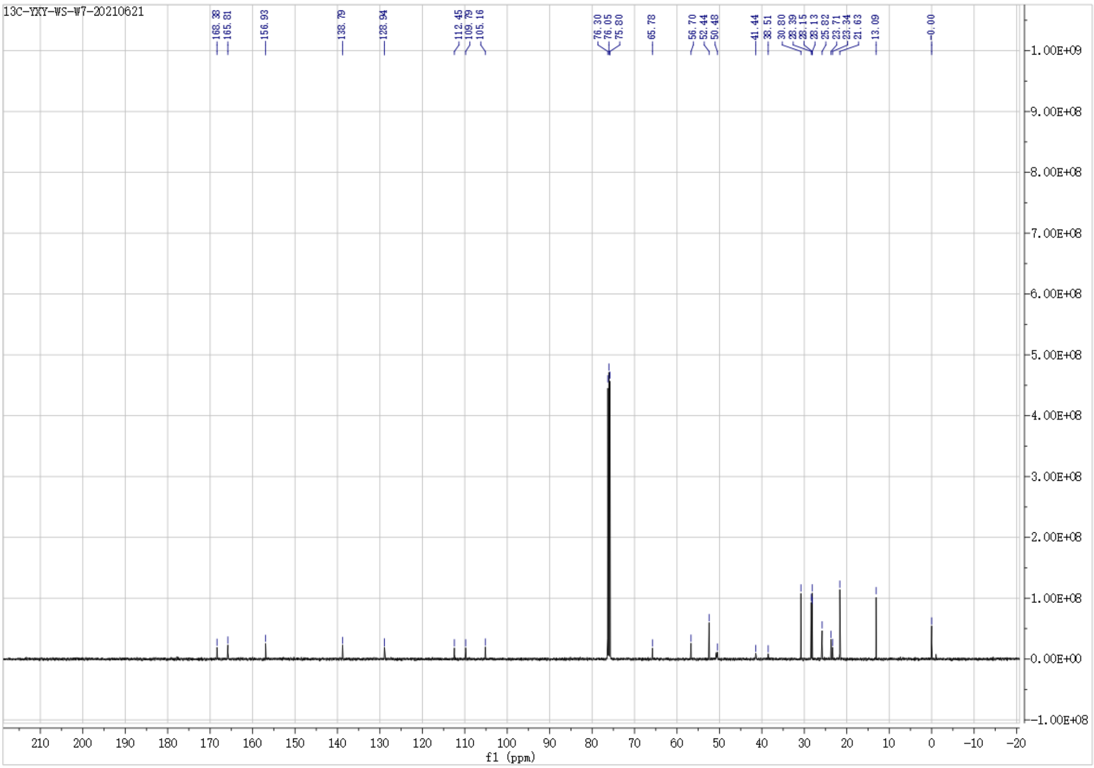

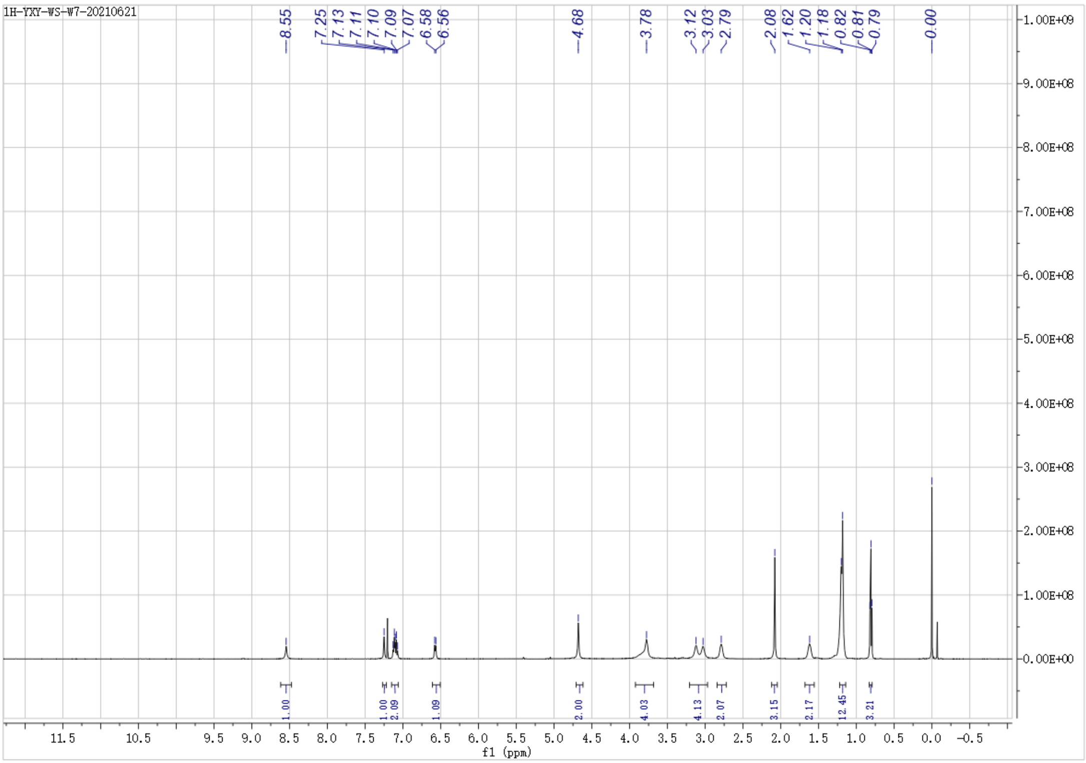

Supplement: Supplemental Material [file IENZ_A_2286183_SM6077.pdf]
